# Large-scale identical-location electron microscopy enables quantifying nanoparticulate electrocatalyst degradation

**DOI:** 10.1038/s41467-026-78329-8

**Published:** 2026-10-10

**Authors:** Ana Rebeka Kamšek, Francisco Ruiz-Zepeda, Domen Tabernik, Jan Vidergar, Anja Logar, Ambrož Kregar, Danijel Skočaj, Goran Dražić, Nejc Hodnik

**Affiliations:** 1 Department of Materials Chemistry, National Institute of Chemistry, Hajdrihova 19, 1000 Ljubljana, Slovenia; 2 Faculty of Chemistry and Chemical Technology, University of Ljubljana, Večna pot 113, 1000 Ljubljana, Slovenia; 3 Institute of Metals and Technology, Lepi pot 11, 1000 Ljubljana, Slovenia; 4 Faculty of Computer and Information Science, University of Ljubljana, Večna pot 113, 1000 Ljubljana, Slovenia; 5 University of Nova Gorica, Vipavska 13, 5000 Nova Gorica, Slovenia; 6 Faculty of Mechanical Engineering, University of Ljubljana, Aškerčeva 6, 1000 Ljubljana, Slovenia

**Keywords:** Electrocatalysis, Nanoparticles, Nanoparticles, Transmission electron microscopy, Electrocatalysis

## Abstract

Fuel cells convert chemical energy into electricity, and their performance relies on electrocatalysts whose durability during operation remains poorly understood. Deep mechanistic insight into electrocatalyst stability is essential to design durable, resource-efficient fuel cells. Nanoparticulate electrocatalysts degrade via diverse nanoscale processes, yet particle-to-particle heterogeneity in structure, support interaction, and local microenvironment make true particle-level quantification and understanding impossible with classical approaches. Here we scale up identical-location scanning transmission electron microscopy, which repeatedly images the very same nanoparticles, to track the structural evolution of hundreds of carbon-supported platinum-cobalt nanoparticles, a prototypical oxygen reduction reaction electrocatalyst. We present a three-step image analysis workflow comprising segmentation, tracking, and degradation-event classification with progressive automation, including machine-learning-assisted detection of overlapping particles. By pairing nanoscale resolution and local history with population-level statistics, the pipeline enables unbiased identification and quantification of degradation pathways across statistically meaningful particle sets. We reveal particle size- and shape-dependent effects, showing that smaller and irregular nanoparticles are more prone to detachment, and provide a data-driven framework for probing electrocatalyst degradation at scale.

## Abstract (web summary)

Fuel cells rely on catalysts made of metal nanoparticles, but tracking how these particles wear down individually has been difficult. Here, the authors build an automated image analysis pipeline to track hundreds of platinum-cobalt nanoparticles and classify the degradation pathways they undergo.

## Introduction

Hydrogen technologies present a promising alternative to fossil fuels, with the potential to decarbonize both transport and stationary power generation. In the clean hydrogen cycle, hydrogen is produced via water electrolysis and then converted to electricity in proton exchange membrane fuel cells (PEMFCs), where hydrogen reacts with oxygen to form water^1^. The inherently sluggish oxygen reduction reaction (ORR) requires a catalyst, and PEMFC operation also depends on external conditions such as humidity and temperature^2^.

High cost remains a major barrier to PEMFC commercialization, largely due to the reliance on platinum, as Pt-based nanoparticles on carbon supports are widely used as ORR electrocatalysts. Optimal catalyst layers must balance platinum utilization, conductivity, and mass transport, while delivering high activity and durability^3^. A similar constraint applies to low-temperature electrolyzers, which also employ Pt. Alloying Pt with a cheaper transition metal (M = Cu, Co, Ni, or Fe) reduces platinum content and can even enhance activity by tuning the electronic structure^4–6^. In practice, real catalyst batches contain nanoparticles with heterogeneous size, shape, and composition^7–11^, making them more complex than model systems used for theoretical predictions^6,12–14^.

Despite progress in activity and cost reduction, durability remains the key challenge for Pt–M electrocatalysts^6^. Stable catalysts must retain their structure and composition under operating conditions, yet several degradation mechanisms (dissolution, detachment, agglomeration, Ostwald ripening, and support corrosion) commonly affect Pt–M/C systems^8,15,16^. These processes are often interlinked. Carbon corrosion can lead to particle detachment or agglomeration, lowering the electrochemically active surface area (ECSA), while dissolution enables Ostwald ripening^15^. Aside from that, all systematic changes to the catalyst structure can be understood as degradation mechanisms – particle migration, surface roughening, amorphization, surface oxidation, and others^17,18^. Because individual nanoparticles differ in atomic structure, composition, support anchoring, and local microenvironment, degradation is intrinsically particle-specific and therefore difficult to resolve with bulk or sparsely sampled measurements^19,20^.

Understanding nanoparticle degradation requires correlating structural features with external stimuli. Scanning transmission electron microscopy (STEM) can provide local and highly precise surface and near-surface structural information down to the atomic scale^21,22^. Random-location ex situ studies provide particle size distributions and statistics across many nanoparticles^23–25^, but yield only averaged insights. In situ liquid-cell TEM offers real-time observation of degradation^26^, yet faces challenges such as beam effects and limited spatial resolution^16^.

Identical-location STEM (IL-STEM) addresses these limitations by imaging the same nanoparticles before and after electrochemical treatment. This enables direct knowledge of local history, providing reliable insights into degradation pathways^6^. IL-(S)TEM was already proven as effective in studying the structure-stability relationship of electrocatalysts^27–32^, including Pt-Co systems, where potential cycling revealed dissolution and redeposition at specific sites^33^. However, IL-STEM studies typically analyze only a handful of particles^30^. While this particle-by-particle bottom-up approach identifies mechanisms, it does not capture the statistical diversity of real electrocatalyst ensembles. Conversely, conventional random-location imaging covers many particles but lacks local history. Furthermore, both approaches miss interparticle interactions, leaving a gap in the quantitative understanding of degradation at scale. Closing this gap requires scaling up IL-STEM to encompass a greater number of nanoparticles and their interactions, together with a reliable automated data analysis pipeline. This would provide statistically meaningful insights while retaining local history.

Algorithms not only accelerate analysis but also minimize human bias and ensure reproducibility^34,35^. Among the various proposed methods for automating (S)TEM image analysis using sophisticated algorithms, a significant number focus on object segmentation^36^. While many have tackled the segmentation of nanoparticles^26,37–42^ and other nanostructures^43–49^, overlapping particles remain difficult to resolve, even with advanced machine learning. Recent advances have addressed the instance segmentation of touching or overlapping nanoparticles in scanning electron microscopy images^50,51^. Addressing this challenge is crucial for reliable large-scale IL-STEM studies of practical electrocatalysts.

Here, we demonstrate how IL-STEM can be scaled up to analyze hundreds of Pt-Co/C nanoparticles subjected to different electrochemical protocols, improving the statistical relevance of degradation analysis and moving the nanoscale IL-STEM field beyond qualitative and small-sample studies. We establish a three-step image analysis workflow comprising segmentation, tracking, and degradation-event classification, progressively automating each step. The pipeline enables robust quantification of degradation mechanisms and reveals which particle types are most susceptible. Experimental observations are complemented with theoretical modeling to contextualize the degradation processes. This study advances IL-STEM from a qualitative, small-sample method to a scalable, quantitative tool, providing statistically grounded, data-driven insights into nanoparticle stability.

## Results

### Scaling up identical-location STEM

A commercial Pt–Co/C electrocatalyst was selected to quantify degradation mechanisms. The catalyst consists of Pt–Co nanoparticles supported on Vulcan XC-72 carbon, with an average particle size of 2–3 nm, a total (Pt + Co) metal loading of ~20 wt%, and a nominal Pt:Co atomic ratio of 1:1. Previous studies showed that, while the overall ratio is 1:1, the sample also contains some pure Co particles, making the Pt-Co nanoparticles richer in Pt^6^.

**Fig. 1:**
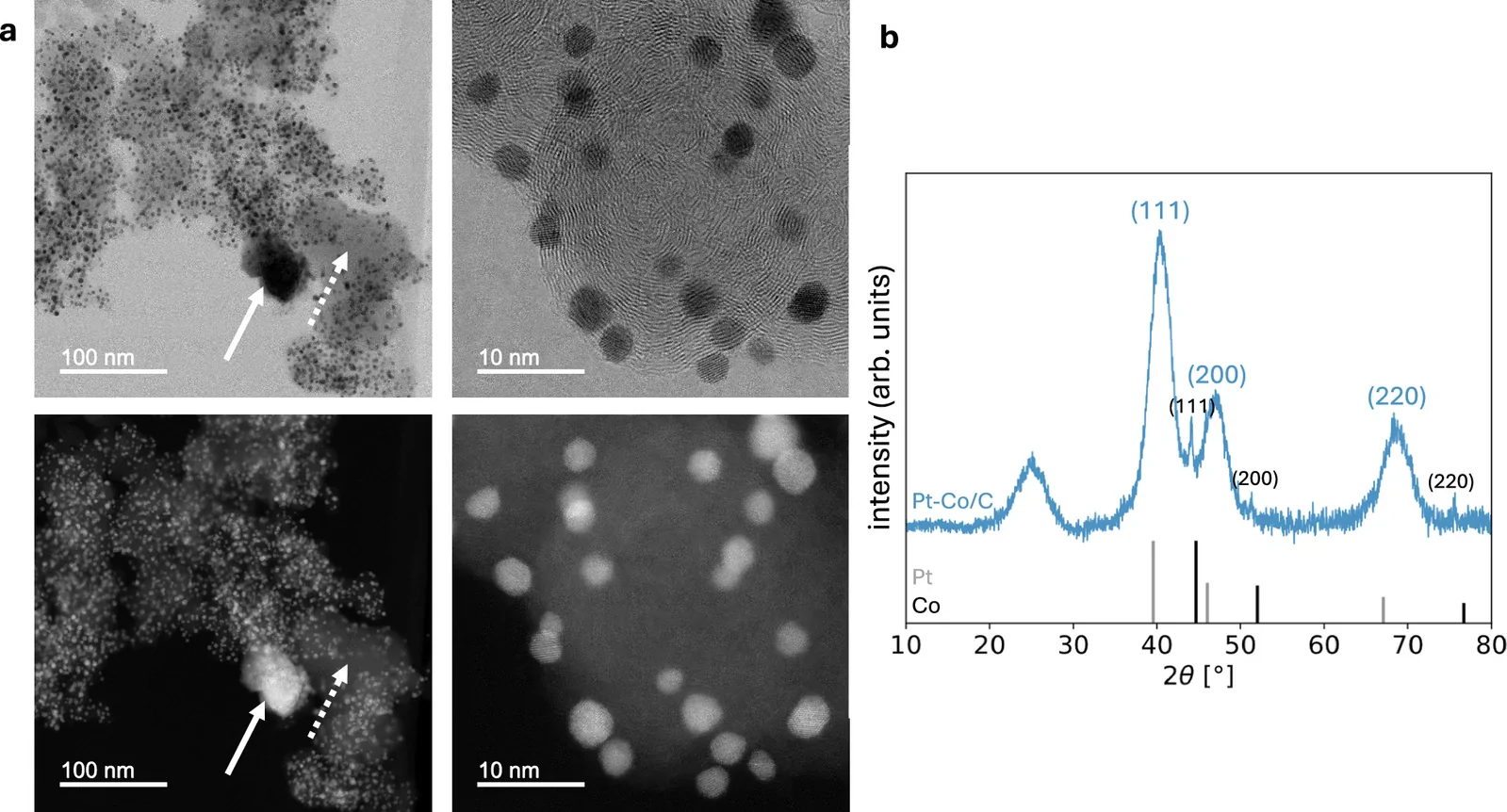
Characterization of the initial state of the Pt-Co/C sample. **a** Bright-field (BF)- (top) and high-angle annular dark-field (HAADF)-STEM (bottom) images at different magnifications. The white full arrow in the left images denotes a less common large particle, and the dashed arrow denotes a more sparsely populated region of the carbon support. **b** X-ray diffraction. Background-subtracted XRD pattern with Miller indices for the Fm-3m Pt-Co and Co crystal phases. Pt and Co reference diffraction patterns are displayed below the Pt-Co/C experimental data. All intensities are displayed with arbitrary units. Source data are provided as a Source Data file.

STEM imaging (Fig. 1a) shows the morphology of the carbon support, populated with a uniform dispersion of Pt-Co nanoparticles, which are typically only a few nanometers in size. However, there are also occasional, order-of-magnitude larger particles, as well as regions where the support is sparsely populated. More STEM images can be found in Supplementary Fig. 1. X-ray diffraction (XRD, Fig. 1b) confirms the Pt-Co Fm-3m disordered alloy phase with small crystallites, as evident from the broad (111), (200), and (220) diffraction maxima, and the carbon support with a broad peak near 25°. Narrow reflections are consistent with pure Co.

For the present study, we have performed energy-dispersive X-ray spectrometry (EDX) on four catalyst regions, spanning hundreds of nanometers in size. HAADF-STEM images of the investigated areas are in Supplementary Fig. 2, and the determined atomic percentages are in Supplementary Table 1. At these lower magnifications, the Pt:Co atomic ratios ranged from 44.7:55.3 to 54.4:45.6. Then, EDX mapping was performed for four catalyst areas at higher magnification, as summarized in Supplementary Fig. 3 and Supplementary Table 2. The Pt:Co atomic ratios were noticeably more diverse, ranging from 48.5:51.5 for an area that exhibited Co-rich sites to 80.4:19.6, underlining the sample’s heterogeneity in chemical composition.

In a previous study, this catalyst was activated using a modified floating electrode (MFE) setup with 200 cycles between 0.05 and 1.2 V versus the reversible hydrogen electrode (vs. RHE) at 300 mV s⁻¹ in an inert atmosphere^6^. IL-STEM imaging at atomic resolution revealed structural variations among individual nanoparticles, but the number of particles that can be studied at this detail is inherently limited, and the impact of the local environment is difficult to assess. To expand the analysis, we revisited lower-magnification IL-STEM image pairs from that dataset (the activation dataset).

**Fig. 2:**
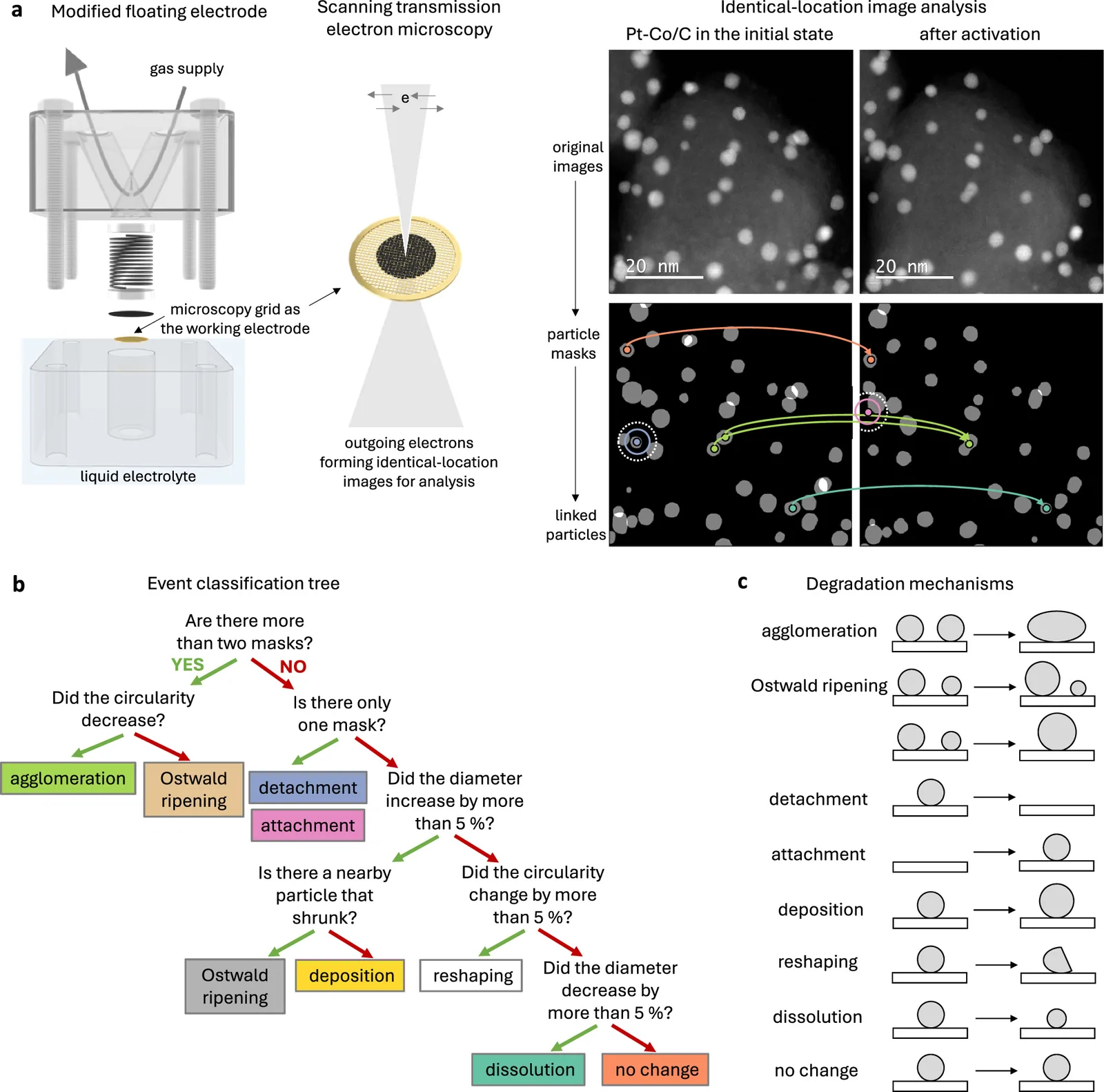
Workflow for identical-location STEM analysis and classification of nanoparticle degradation. **a** Modified floating electrode setup coupled with IL-STEM imaging, yielding paired HAADF-STEM images of Pt-Co/C before and after activation (200 cycles, 0.05–1.2 V_RHE_, 300 mV s^-1^, 4 M HClO_4_). Imaged nanoparticles were converted into particle masks and linked across image pairs to track individual particles. Colored markers denote examples of events with colors corresponding to the ones in (**b**). **b** Decision tree used to classify particle-level events into degradation mechanisms. Color coding was later used for consistent plotting. **c** Schematic depictions of individual degradation mechanisms.

Figure 2a illustrates the workflow: IL-STEM images were acquired before and after catalyst activation in the modified floating electrode. Nanoparticles were annotated in both image sets, linked across the before-and-after states, and grouped into degradation events. Most events involved two particle masks (one per image), cases where a particle appeared in only one image were recorded as single-mask events, while merging of several particles was represented by multiple masks. Here, a mask is a binary image in which pixels corresponding to the particle are marked, providing a clear outline of its shape.

Events were categorized into degradation mechanisms using a decision tree (Fig. 2b). The decision tree incorporates simple geometric descriptors (particle number, diameter, circularity, and local environment) to assign each event to a distinct degradation mechanism or to confirm no significant change (Fig. 2c). This framework reduces complex degradation phenomena to a set of interpretable rules, enabling mechanism-specific classification across large datasets. In doing so, IL-STEM evolves from a primarily descriptive tool into a robust quantitative method for probing electrocatalyst stability.

Most outcomes can be traced to two primary degradation mechanisms: platinum dissolution and carbon corrosion. Under the present conditions, platinum dissolution is known to trigger cobalt dissolution^6^, so the shrinkage observed here (defined as dissolution in Fig. 2b) refers to overall nanoparticle loss rather than to a single element. The decision tree was designed based on prior studies of Pt-based nanoparticulate electrocatalyst degradation^15,27^. A 5% tolerance was included in the selected criteria to account for segmentation uncertainty, typically at least one pixel around a particle, and to avoid labeling tiny variations as degradation.

Here, Ostwald ripening requires a shrinking and a growing particle to be in close spatial proximity within the same image. This approach cannot capture longer-range dissolution-redeposition processes where dissolved species migrate and deposit on distant particles, which would be classified as separate dissolution/detachment and deposition events. It does, however, distinguish between complete Ostwald ripening, where the smaller particle dissolves entirely, and other Ostwald ripening events, where both particles remain but with reduced and increased sizes, respectively. Another inherent limitation is that for detachment events, a particle that disappears from an image could, in principle, be either detached or fully dissolved. For practical reasons, disappearing particles are conservatively classified as detached.

**Fig. 3:**
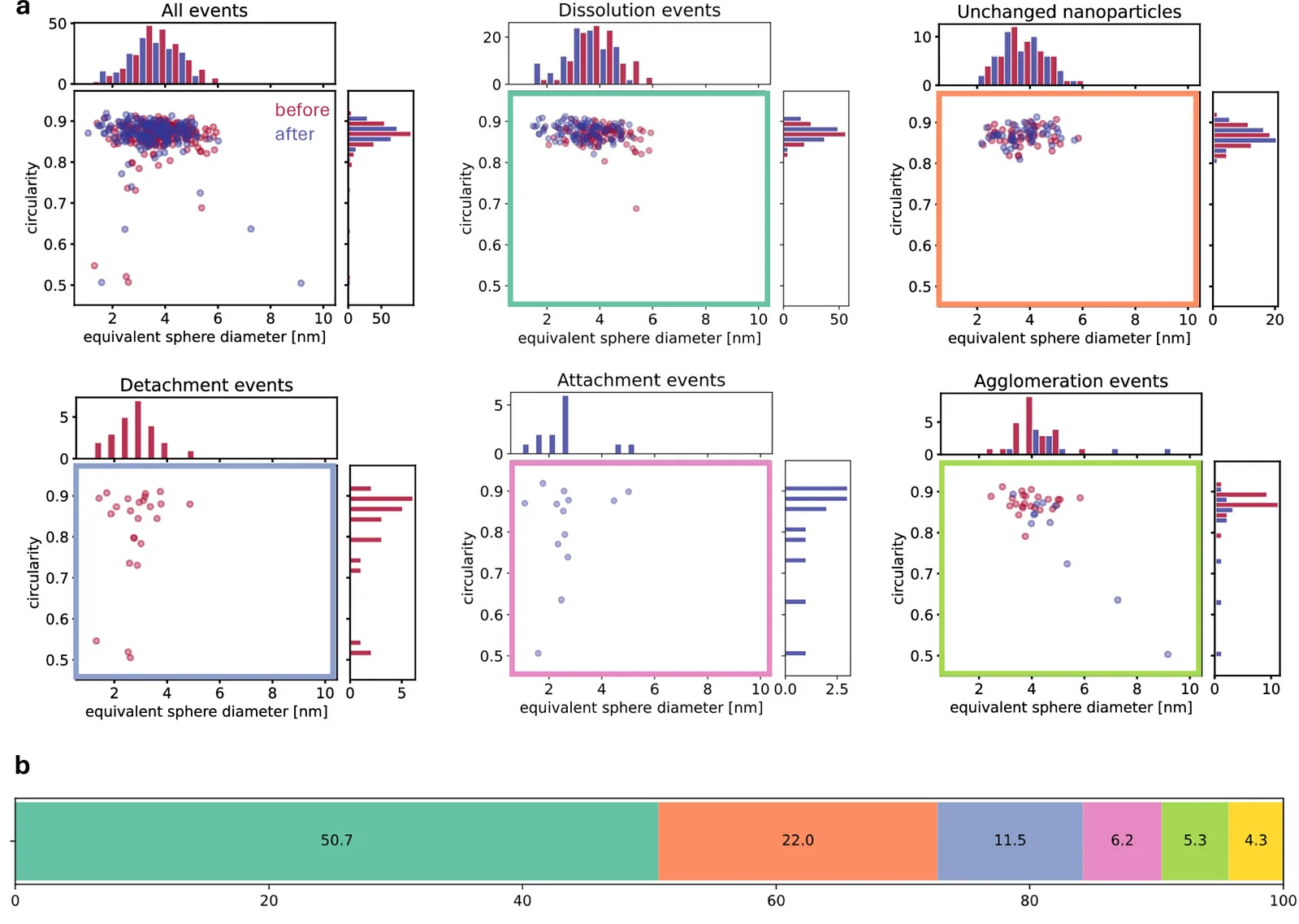
Degradation events for Pt-Co nanoparticles during potential cycling activation. **a** Scatter plots with histograms showing circularities and equivalent sphere diameters before (red) and after (blue) activation for all events and individual degradation mechanisms (200 cycles, 0.05–1.2 V_RHE_, 300 mV s^-1^, 4 M HClO_4_). **b** Percentages of all mechanisms (*n* = 209; turquoise – dissolution, orange – no significant changes, blue – detachment, pink – attachment, green – agglomeration, yellow – deposition). Source data are provided as a Source Data file.

The activation dataset contained 209 events, which were classified using the decision tree. Figure 3a shows the size and circularity distributions before and after activation for mechanisms with at least ten events. Most nanoparticles are near-spherical with diameters of 2–6 nm. Supplementary Table 3 summarizes mean values and standard deviations for each category.

Examining dissolution events, defined as particles getting smaller, shows that they are most prevalent among nearly spherical nanoparticles. Nanoparticles that remain unchanged display a similar circularity range but a slightly smaller average diameter. Figure 3b displays the frequency of each degradation mechanism. These two groups of events account for about one-half and one-fifth of all events, respectively.

Smaller nanoparticles are more prone to complete detachment from the carbon support, and newly attached particles observed only after activation also tend to be smaller. During agglomeration, small and circular nanoparticles combine into larger, less circular agglomerates, whereas deposition events typically yield larger spherical particles. Detachments make up more than 10% of all events, and attachment, agglomeration, and deposition each account for 4–6%. No other mechanisms were identified.

These results highlight correlations between particle characteristics and degradation pathways, but the infrequency of some mechanisms limits firm conclusions. For instance, there are only 13 attachment events, so a few occurrences can significantly influence the trends. While the activation dataset of 209 events from six pairs of IL-STEM images provides a useful proof of concept, larger datasets are needed for more robust conclusions.

To this end, a second dataset, referred to as the degradation dataset, was collected for the same electrocatalyst. The MFE setup was used for an activation protocol of 200 cycles between 0.05 and 1.2 V vs. RHE at 300 mV s^-1^, followed by a degradation protocol of 5000 cycles between 0.4 and 1.2 V at 1 V s^-1^. The degradation protocol was chosen to enable easier comparison with a previous study^6^, and the wide potential window was expected to induce notable enough changes to nanoparticles to fully showcase the capabilities of a scaled-up IL-STEM^52^. All experiments were performed at room temperature (approx. 22 °C).

IL-STEM images were recorded before and after the entire protocol. Supplementary Fig. 4 shows the initial and final cyclic voltammograms for both protocol segments on two TEM grids. Activation yielded a platinum-like signal within the chosen potential window under an inert atmosphere. During degradation, only minor changes occurred: the final voltammogram exhibited slightly lower geometric-area-normalized current densities, which is qualitatively consistent with the loss of Pt surface area. The full-range cyclic voltammogram in Supplementary Fig. 5, recorded after degradation, similarly exhibits lower measured currents compared to the last cycle of activation.

**Fig. 4:**
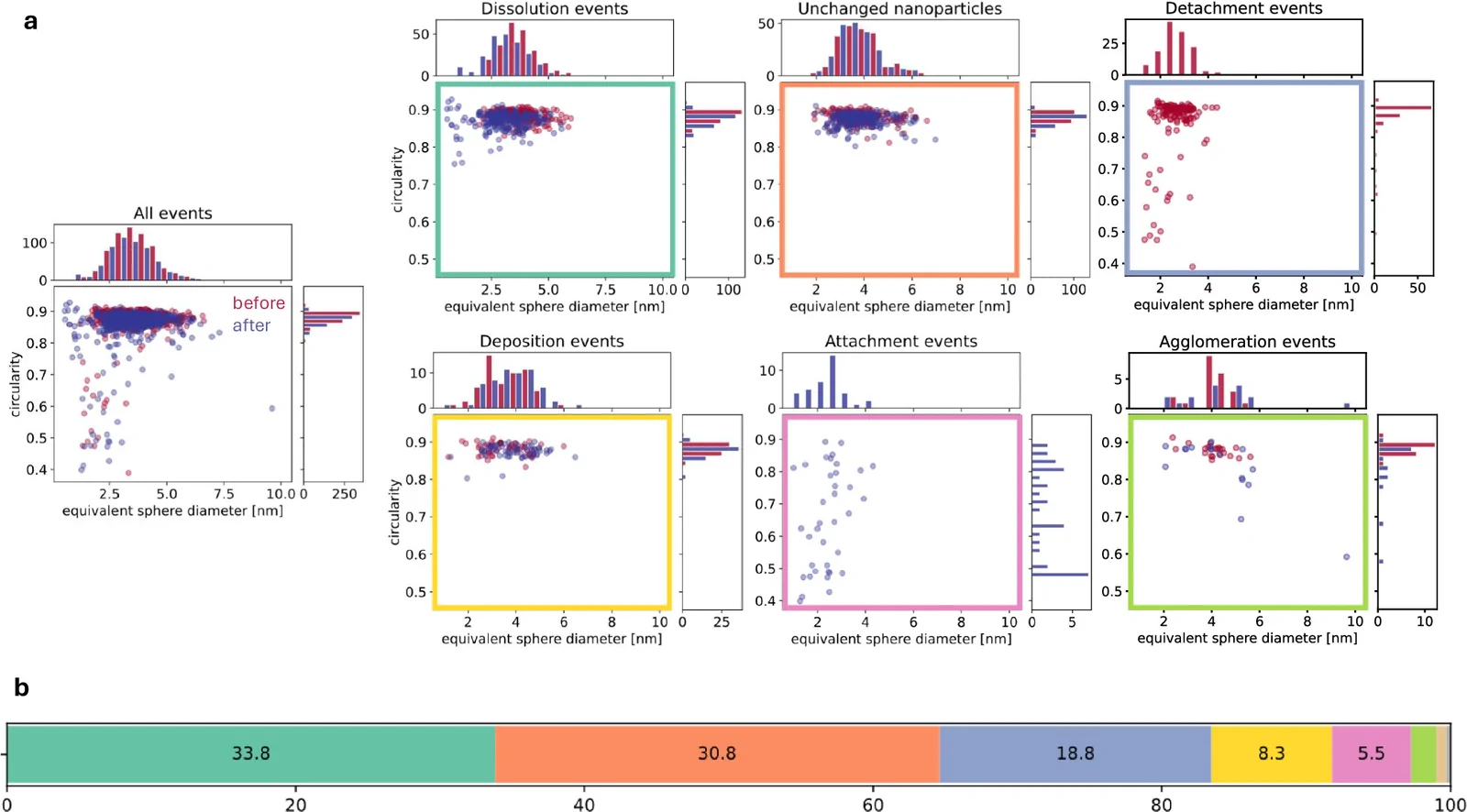
Degradation events for Pt-Co nanoparticles during potential cycling activation, followed by degradation. **a** Scatter plots with histograms showing circularities and equivalent sphere diameters before (red) and after (blue) activation (200 cycles, 0.05–1.2 V_RHE_, 300 mV s^-1^, 0.1 M HClO_4_) and degradation (5000 cycles, 0.4–1.2 V_RHE_, 1 V s^−1^, 0.1 M HClO_4_) for all events and individual degradation mechanisms. **b** Percentages of all mechanisms (*n* = 696; turquoise – dissolution, orange – no significant changes, blue – detachment, yellow – deposition, pink – attachment, green – agglomeration, brown and gray – Ostwald ripening). Source data are provided as a Source Data file.

A larger IL-STEM dataset was collected, resulting in 696 events that were analyzed as previously. Figure 4a includes the size and circularity distributions for all events and individual degradation mechanisms, and Fig. 4b summarizes their frequencies. Supplementary Table 4 lists average sizes and circularities for each mechanism.

The degradation dataset largely followed the same trends as the activation dataset. The activation protocol likely stabilized the catalyst structure, leading to only minor additional changes during subsequent degradation. The particle distribution in the initial state was nearly identical, confirming that we obtained images of representative areas.

Several conclusions are consistent across both datasets. Dissolved and unchanged nanoparticles show similar diameter and circularity distributions, while smaller particles are predominantly involved in attachment and detachment processes. Some findings are more pronounced in the degradation dataset due to improved statistics. With more than three times the number of events, less frequent mechanisms could be examined with greater confidence. For example, attachment events exhibit a broader distribution of circularity. Among the particles in the initial images, the ones with lower circularities are generally recognized as detachment events, although this category also includes a far larger number of spherical particles.

Dissolution remains the dominant mechanism in both datasets, followed by unchanged particles, but the degradation dataset shows a higher proportion of unchanged particles, detachments, and depositions, and fewer dissolutions and agglomerations. While the initial diameter and circularity distributions showed considerable overlap for dissolved and unchanged nanoparticles, it should be stressed that some particles designated as unchanged may be electrochemically inactive due to poor electrical contact.

The degradation dataset also revealed a few Ostwald ripening events, absent in the manually annotated activation dataset. Although they might be interpreted as agglomeration followed by reshaping, the scarcity of reshaping events makes this unlikely. The higher frequency of detachments reduces accessible surface area, consistent with the lower currents observed after degradation, as indicated by the diminished Pt features in Supplementary Fig. 4.

A larger dataset certainly improves the statistical analysis. Nevertheless, even the activation dataset, developed from merely six pairs of IL-STEM images, provided meaningful insights into the behavior of Pt-Co nanoparticles under electrochemical stimuli. To substantiate that our sample size is adequate for this catalyst system, we performed a convergence analysis in which the mean particle diameter and circularity were calculated for randomly drawn subsets of increasing size (Supplementary Note 1, Supplementary Fig. 6). The relative standard error of the mean for circularity remains below 1% across all subset sizes tested, while that of particle diameter falls below 2% beyond 150 particles and below 1% beyond 400 particles, confirming that a randomly selected dataset of several hundred nanoparticles is sufficient to capture population-level degradation trends within this particle ensemble.

Interestingly, unchanged nanoparticles were more frequent in the degradation dataset despite the longer electrochemical treatment. Supplementary Tables 3 and 4 show no clear patterns apart from slight shrinkage during activation. Several factors may account for this apparent stability. A comparison of Figs. 3b and 4b could suggest dissolution followed by redeposition, restoring particle size. A prior atomic-resolution study of Pt-Co nanoparticles during potential cycling likewise observed both dissolution and new atomic columns, consistent with redeposition^6^. Supplementary Fig. 7, which tracks selected particles across all three stages and shows examples of size reduction during activation followed by partial recovery during degradation, supports this possibility. These images are presented qualitatively, as multi-step IL-STEM introduces additional experimental challenges that can compromise quantitative analysis.

Some particles may also have been shielded from the electrolyte by carbon layers or complete embedding, making them inactive. Because most imaged particles overlap with the carbon support, the extent of this effect is hard to judge. Another factor to consider when comparing the unchanged nanoparticles from the activation and degradation datasets is electrolyte pH, previously reported to influence platinum dissolution under cyclic voltammetry conditions^53^. Finally, some nanoparticles may simply possess inherently higher stability.

To connect these insights to changes in the chemical composition, we have performed EDX for the sample in the as-purchased state, after potential cycling activation (200 cycles, 0.05–1.2 V_RHE_, 300 mV s^-1^, 0.1 M HClO_4_) and after degradation (5000 cycles, 0.4–1.2 V_RHE_, 1 V s^-1^, 0.1 M HClO_4_). The determined Co and Pt atomic percentages in Supplementary Table 1, and the corresponding HAADF-STEM images of the investigated areas in Supplementary Fig. 2, confirm that both electrochemical protocols induce changes to the catalyst composition, with activation causing somewhat more pronounced changes to the Co-to-Pt ratio than degradation. The results indicate that, on average, the composition becomes richer in Pt both after activation and degradation. However, different imaged areas exhibit varied outcomes, and not all show an increase in Pt, which may be attributed to the inherent heterogeneity of the sample. Identical-location EDX for four regions of interest at higher magnification, performed before and after potential cycling activation, confirmed notably different ratios between Pt and Co, as evidenced by the elemental maps in Supplementary Fig. 3 and atomic percentages in Supplementary Table 2. Co-rich sites, observed in the initial state, influence the determined atomic percentages, and do not persist after activation.

In platinum alloys, including the presently studied Pt-Co, the dissolution trend of the less noble metal is related to platinum dissolution^6^. During anodic polarization, the formation and restructuring of surface Pt oxides create low-coordination Pt defects that are prone to dissolution. Cathodic dissolution occurs when Pt oxide is reduced, thereby restructuring the surface and inducing the dissolution of Pt defects. This process exposes previously protected, less noble metal atoms within the alloy lattice, triggering their subsequent dissolution. Therefore, stabilizing platinum is key to stabilizing its alloys.

The influence of the potential window extends beyond general dissolution trends. Existing literature confirms that both primary degradation mechanisms, metal dissolution and carbon corrosion, depend strongly on the applied potential window. Higher upper potential limits, encountered for instance during start-up/shut-down conditions in fuel cells, significantly increase both carbon corrosion and platinum dissolution, the latter due to more pronounced nanoparticle surface oxidation. Conversely, a narrower potential window reduces degradation rates. The primary mechanisms also trigger secondary ones, leading to loss of electrochemically active surface area and catalytic activity over time. Nevertheless, even a narrow window will produce noticeable degradation under prolonged operation. Thus, the choice of electrochemical protocol importantly shapes the reported degradation trends^52^. Beyond the potential window, the testing environment itself matters: aqueous environments are not directly comparable to a membrane electrode assembly. One study found that extending the potential window and adding Pt ions to the electrolyte yielded particle size distributions and alloy compositions closer to those measured in a membrane electrode assembly^54^.

Large-scale IL-STEM data provide quantitative insight into which nanoparticle types are more likely to maintain their structure during potential cycling. Typically, spherical nanoparticles of average size are the most stable, although similar particles may also dissolve. In contrast, small or irregularly shaped nanoparticles rarely remain unchanged, suggesting that limiting their number could improve catalyst design.

While potential imaging artifacts arising from minor grid buckling or tilting of the carbon support during cycling could theoretically cause an apparent shape change, we find this unlikely to significantly impact our statistical analysis. The vast majority of particles are roughly isotropic, making their 2D projection less sensitive to rotation, and no large-scale, coherent changes in particle positions or shapes indicative of such artifacts were observed. Significant sample damage due to rinsing the grid with water before imaging was not observed. The limited depth of focus in STEM introduces a margin of error when annotating out-of-focus particles, underscoring the importance of imaging a sufficient number of nanoparticles. The current analysis cannot address the effects of local composition or crystal structure, which would require a multi-modal IL-EM.

Previous studies support these observations. Time- and potential-resolved dissolution experiments showed that 3 nm Pt nanoparticles dissolve more readily than 30 nm ones^55^. Similarly, Pt particles between 2 and 10 nm dissolve faster at smaller sizes under wide potential cycling windows^56^. These trends are also consistent with particle-size-dependent potential–pH diagrams^57^. While shape effects have been explored mostly in the context of deliberately synthesizing defined morphologies, fewer studies compare stability across particle shapes within a single sample^52^. The observed modest degree of Ostwald ripening for both cycling protocols could be attributed to both relatively mild conditions of MFE testing compared to membrane electrode assembly studies. In the MFE geometry, dissolved Pt species can diffuse away from the catalyst layer and become diluted in the larger bulk electrolyte volume, limiting local redeposition onto larger nanoparticles. This effect is likely reinforced by the narrow particle size distribution of our catalyst, which reduces the driving force for Ostwald ripening. Applying scaled-up IL-STEM to broader datasets with diverse degradation protocols would allow predictive correlations between particle size, shape, environment, and electrochemical conditions, ultimately guiding the design of both stable catalysts and optimized operating protocols.

Complementary electrochemical characterization of the same Pt-Co/C catalyst by rotating disk electrode qualitatively supports these findings. As shown in Supplementary Fig. 8, the ECSA decreased modestly after degradation relative to activation. Ex-situ EDX analysis also indicates a more pronounced shift in the Pt:Co ratio following activation than after degradation, although the absolute compositions are influenced by sample inhomogeneity, and the comparison to the present study is not straightforward because of a different way to carry out electrochemical protocols^6^.

There are potential risks and limitations to this type of analysis. The main trade-off is resolution: while atomic-resolution IL-STEM can provide detailed insights into individual nanoparticles, extending such analysis to hundreds of particles is not yet feasible. Achieving this throughput would require fully automated atomic-resolution STEM imaging, a technical frontier still to be realized. In addition, systematic errors such as grid wrinkling, imperfect annotation, and limited statistics on rare degradation mechanisms may also influence the results. On the image analysis side, manually annotating hundreds of nanoparticles is time-intensive and limits the practicality of this approach, motivating the development of automated analysis tools.

### Step-by-step automation of large-scale IL-STEM analysis

In the long run, the process of preparing the dataset should be automated, especially particle segmentation. This task remains challenging even for advanced algorithms because images often contain overlapping nanoparticles. Although the Pt-Co particles were generally well dispersed on carbon due to the relatively low metal loading, several instances of overlap were observed.

In HAADF-STEM images, the overlapping region appears significantly lighter than the rest of the nanoparticle. A classical algorithm could, in principle, correctly assign the overlapping region to both neighboring nanoparticles, but the process becomes more complex when there are multiple overlapping nanoparticles or when one particle is out of focus. Watershed segmentation is sometimes used, but it typically underestimates particle areas by splitting the overlap between neighbors. More advanced approaches are therefore needed.

**Fig. 5:**
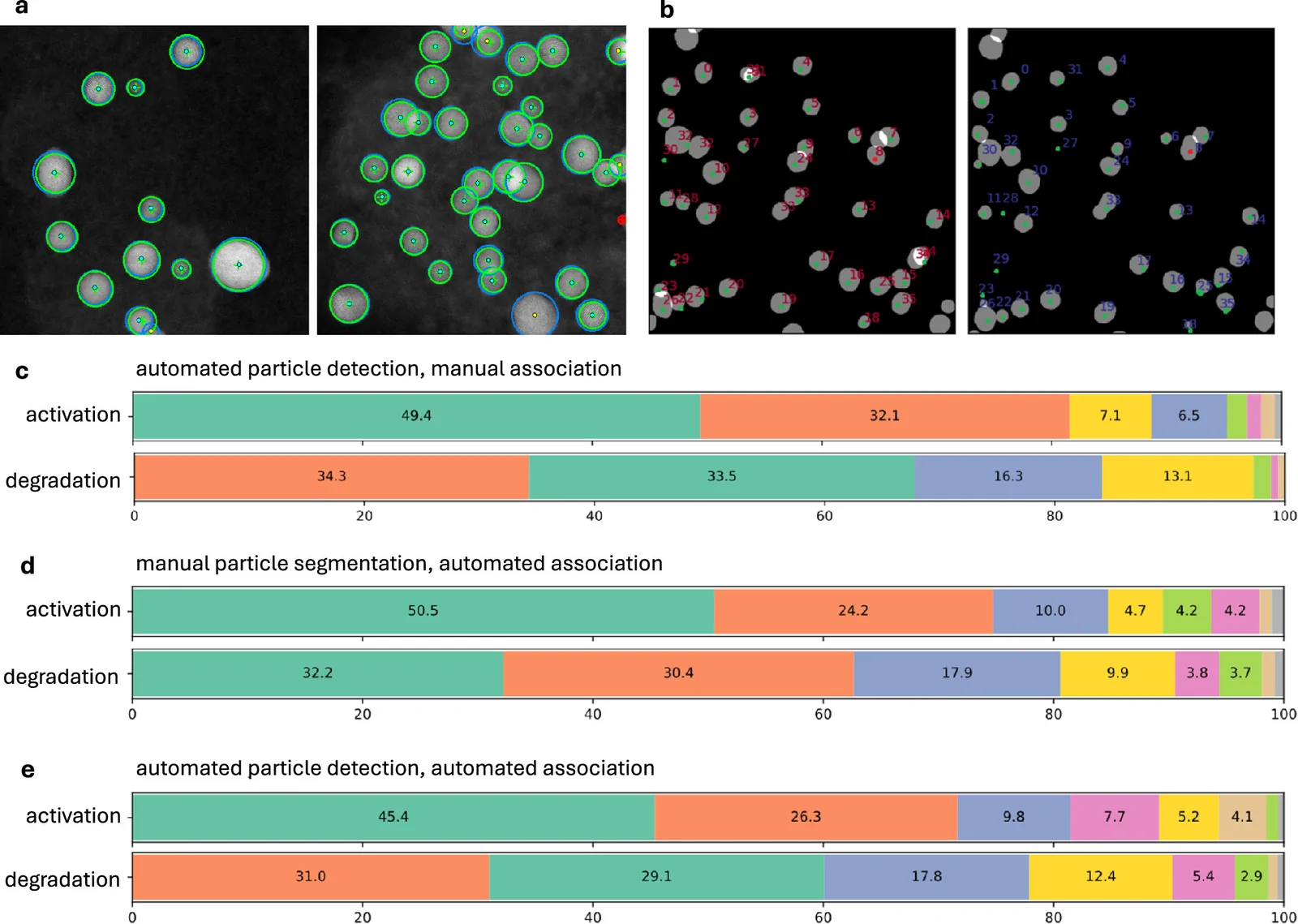
Step-by-step automation of large-scale IL-STEM data analysis. **a** Examples of two test images with the detected Pt-Co nanoparticles shown in green and the ground truth in blue circles. Yellow dots denote disregarded edge particles, and the red marker denotes a false result. **b** Connecting particles in IL-STEM with a heuristic algorithm for one set of IL-STEM images with manually segmented particles, before (left) and after (right) potential cycling activation. The panels include event numbers and green and red markers for correctly and incorrectly connected nanoparticles. **c–e** Event analysis results in terms of percentages of all mechanisms after automated dataset preparation. Turquoise – dissolution, orange – no significant changes, blue – detachment, yellow – deposition, pink – attachment, green – agglomeration, brown and gray – Ostwald ripening. Source data are provided as a Source Data file.

Here, we implemented a neural-network-based particle detection to tackle the challenge of partially overlapping supported nanoparticles. The technical details about this step are available in Supplementary Note 2. Once trained, the network identified nanoparticles in new images and reported their radii and circularities. Because some annotated images were reserved for training, the total number of analyzed particles was smaller. Two representative test images with successful detection of overlapping nanoparticles are shown in Fig. 5a. The neural network correctly detects instances of nanoparticles, even when there is significant overlap among them.

Model performance was evaluated against manual annotations. Although the model predicts circularity with good numerical accuracy, achieving a relative error of just 2.19%, particles with low circularity remain challenging, likely due to their rarity in the training set. Particle size predictions had a relative error of 6.35%, but deviations were not biased toward particular sizes.

The particle detections were used for analysis as previously. Fig. 5c shows mechanism frequencies for the activation and degradation datasets, obtained with automated particle detection and manual particle association. Supplementary Tables 5 and 6 summarize particle sizes and circularities by mechanism, and Supplementary Figs. 9,10 contain the scatter plots with histograms.

Compared to the ground truth values in Fig. 3b for the activation dataset, dissolution is again recognized as the most frequent degradation mechanism, followed by unchanged nanoparticles. When comparing the degradation results to the ground truth in Fig. 4b, these two outcomes are swapped while their percentages remain similar, and detachment and deposition events correctly follow them.

However, there are discrepancies in the less frequently occurring mechanisms, especially in the smaller activation dataset. Trends such as smaller diameters for attached and detached particles were still reproduced. By contrast, circularity-based distinctions were less clear, partly due to difficulties in recognizing agglomerated particles, which likely contributed to missed agglomeration events and the higher fraction of depositions. Similarly, this workflow does not capture the full morphological diversity of attached particles. In the degradation dataset, these issues were less pronounced, indicating more robust performance. While particle detection is robust to moderate particle overlap, its potential application to catalysts with higher metal loadings requires strategic imaging of thinner regions to avoid extreme crowding.

Nonetheless, the neural-network-based particle detection combined with manual particle association still returns reliable insights into the dominant degradation mechanisms for Pt-Co nanoparticles undergoing potential cycling. Crucially, the network handled overlapping nanoparticles well, a prerequisite for scaling up IL-STEM analysis.

In addition to segmentation, connecting masks between IL-STEM images can also be automated. Here, we implemented a heuristic algorithm to form events based on potential degradation mechanisms. The process involved several steps: aligning the two images, matching particles with overlapping masks, identifying particles missing in one image or merged into larger features, and resolving any remaining candidates. This workflow was developed on the activation dataset without further tuning before applying it to the degradation dataset. Results for a representative image pair are shown in Fig. 5b.

Figure 5d summarizes the outcomes of automated particle association applied to manually segmented particles. The dominant mechanisms of dissolution, unchanged particles, and detachment were correctly identified in both datasets. Discrepancies arose mainly in less frequent categories, such as attachment and deposition during activation, and some activation events were misclassified as Ostwald ripening. In the degradation dataset, the mechanism order was consistent, although attachment increased and agglomeration decreased. These findings confirm that the algorithm was not overfit to the activation dataset and can be used for other images.

Supplementary Tables 7 and 8 compare particle sizes and circularities obtained with automated association of manually annotated particles (scatter plots with histograms in Supplementary Figs. 11,12). While the results generally agree, 10% fewer events were analyzed due to missed matches, especially in crowded regions with overlapping nanoparticles. Some overlooked and mismatched particles lead to deviations in determined particle sizes and circularities, especially with less common mechanisms, where a few outliers can significantly affect results. For instance, in the degradation dataset, agglomerated nanoparticles and particles post-complete Ostwald ripening are not larger than the initial particles. Additionally, particles exhibit a narrowed circularity range after attachment, with non-circular particles often being excluded from analysis.

Despite these limitations, the approach provides useful insights into the prevalence of different degradation mechanisms, particularly in images of well-dispersed nanoparticles. Like the decision tree, the heuristic process can be tailored to expected physical processes between two IL-STEM images. Once implemented, it can be applied repeatedly, saving analysis time.

Finally, we consider the fully automated pipeline, which integrates both particle detection and association across IL-STEM image pairs. Results are shown in Fig. 5e, Supplementary Figs. 13,14, and summarized in Supplementary Tables 9,10. Despite accumulated uncertainty, the core findings are preserved. Dissolution and unchanged nanoparticles remain the two most frequent outcomes with similar percentages to the ground truth, albeit swapped in the degradation dataset, while detached particles are consistently smaller. For attached particles, the trend of reduced size is also present in most cases, though it becomes less clear when both detection and linking are fully automated, reflecting increased size variance.

Dataset size influences reliability. In the smaller activation dataset, detection errors disproportionately affect the classification of rare mechanisms. By contrast, the degradation dataset shows fewer discrepancies, and random errors tend to be statistically diluted. This highlights a central consideration: automation is more reliable and meaningful when applied to large datasets. As data volume increases, statistical robustness improves, making manual processing impractical at scale. While automated methods effectively reveal dominant trends despite minor errors, manual annotation is still best for analyzing rare or subtle phenomena, even if it means fewer particles are examined.

The most notable is the confusion between agglomeration and deposition, especially in the activation dataset, where agglomeration is underrepresented and deposition overestimated. This distinction is not trivial, as it can directly affect the scientific understanding of material behavior. That said, these two mechanisms are not among the most frequent, which somewhat limits the broader impact of this issue.

Each layer of automation reduces accuracy but offers substantial efficiency gains. The trained detection model and association algorithm can now be deployed rapidly across large datasets that would be impractical to process manually. The fully automated pipeline reliably captures dominant mechanisms and size trends. We recommend full automation primarily for large-scale studies, and only once the robustness of the procedure has been adequately validated.

### Theoretical modeling of Pt-Co particle evolution

The experimental findings were further contextualized with theoretical models. Eleven nanoparticles from one IL-STEM image set in the activation dataset were selected, and two primary degradation mechanisms (metal dissolution and carbon corrosion) were modeled to describe common pathways such as dissolution, detachment, and agglomeration. While applied to a limited subset of particles, the modeling is designed to provide mechanistic context for the statistical trends, not to replicate the full dataset. Detailed mathematical formulations and parameters are provided in Supplementary Note 3 and Supplementary Table 11.

**Fig. 6:**
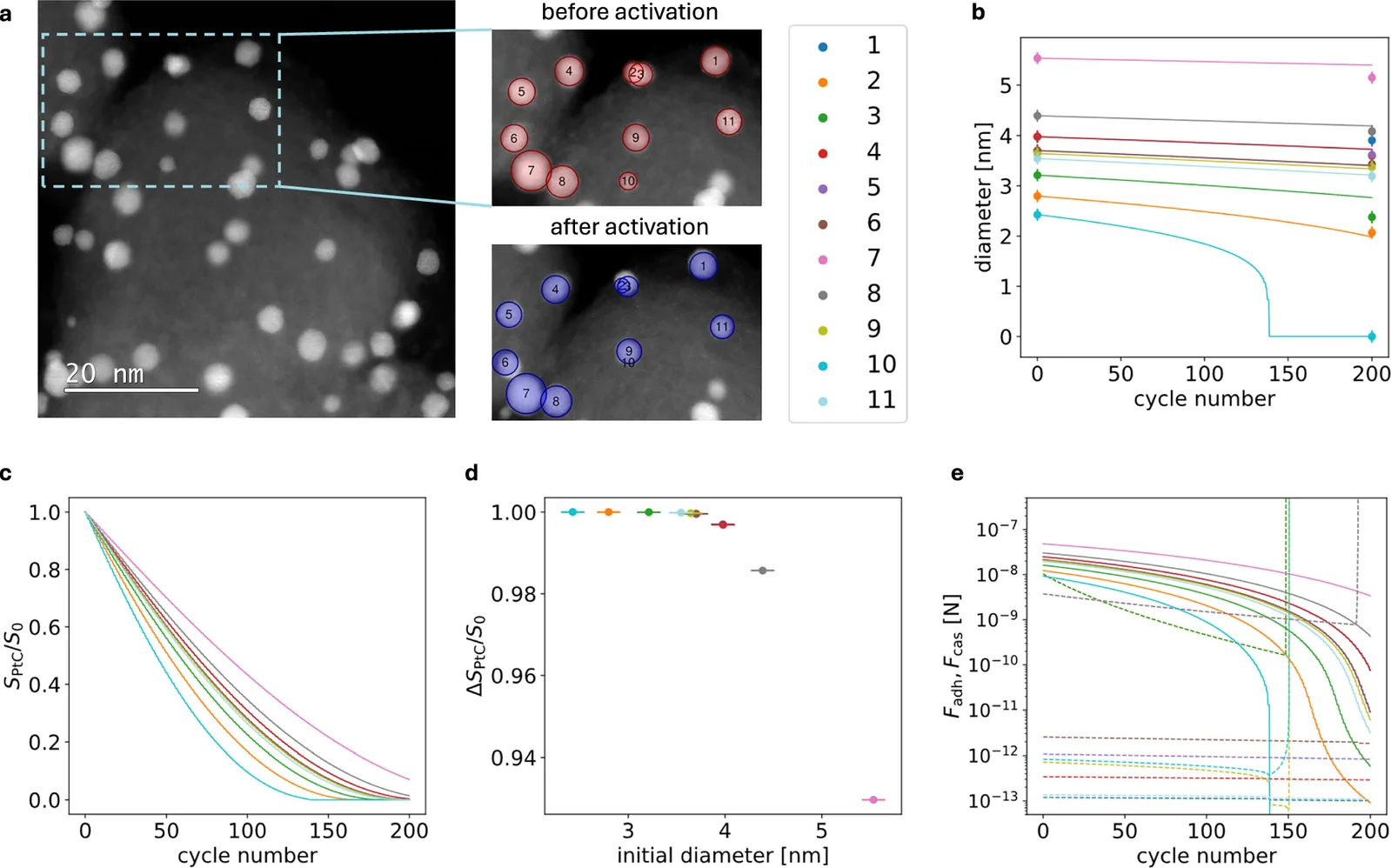
Theoretical modeling of particle evolution during potential cycling activation. **a** A depiction of particles that were used for modeling with numerical indices and the color legend. **b** Tracking particle diameters. Lines denote the modeling result, and circular markers with error bars denote experimental values from STEM images. **c** Tracking the contact surface between PtCo nanoparticles and the carbon support. Values are normalized to the initial contact surfaces for each nanoparticle. **d** Changes in the normalized contact surface, plotted against the initial nanoparticle diameter. Error bars in **b** and **d** denote the estimated experimental uncertainty in determining the particle diameters. **e** Tracking the adhesion (full lines) and attractive Casimir forces (dashed lines). Source data are provided as a Source Data file.

Fig. 6 summarizes how particle size and embedment in the carbon support evolve during the activation protocol. Geometric parameters before and after simulation are listed in Supplementary Table 12, while Fig. 6a shows the selected Pt-Co nanoparticles and their color assignments used in the plots.

The simulations (Fig. 6b) indicate that dissolution proceeds more rapidly for smaller particles, accelerating as size decreases. This is coupled with enhanced carbon corrosion, reflected in large changes in particle–support contact area (Fig. 6c, d). Size reduction can directly cause dissolution or promote detachment, either through complete loss of the particle or through weakened adhesion from interfacial corrosion. This insight helps address an inherent ambiguity in IL-STEM: a particle that disappears from the image could have either physically detached or dissolved completely while remaining on the support. The simulations confirm that complete dissolution is a viable pathway for small particles under the studied conditions.

Overall, corrosion appears substantial, with interfacial area changes comparable to initial values under the simplifying assumption of a π/2 contact angle. It should be noted that this is the result for a simplified model that does not consider all aspects of the inherently complex catalyst structure. Assuming a uniform contact angle may be questionable, and a lower initial value for detached particles could lead to the same pattern of agglomeration at a much slower carbon corrosion rate. In any case, once a particle shrinks below a critical size, it is more likely to be lost through dissolution or detachment.

While nanoparticle degradation was investigated in detail using particle masks, IL-BF-STEM images (Supplementary Fig. 15) confirm localized changes to the carbon support after activation, consistent with the onset of corrosion. However, a direct quantitative correlation between support changes and specific nanoparticle degradation pathways is challenging from IL-STEM images due to the weaker signal and complex contrast of carbon. For the same reason, automated analysis of carbon support changes in these images remains difficult.

Both dissolution and corrosion thus play central roles in detachment. The process of agglomeration, as explained using adhesion and attractive forces in Fig. 6e, provides additional insight. As particles shrink or corrode, adhesion to carbon decreases. At the same time, attractive forces between nearby particles, which depend on their size and distance, can become more significant. For some particles (e.g., 9 and 10 in Fig. 6a), attraction eventually surpasses adhesion, leading to detachment and reattachment to a neighbor.

These models provide mechanistic insight inaccessible to IL-STEM alone. Although simplified relative to experimental reality, they help explain how nanoscale processes such as corrosion-driven detachment and agglomeration govern the long-term structural evolution of catalyst layers. The experimentally informed modeling, though applied to a limited set of particles, offers a continuous perspective on degradation mechanics, thereby complementing the discrete snapshots provided by IL-STEM.

## Discussion

We developed a method to quantify the contributions of different degradation mechanisms in a commercial Pt-Co/C oxygen reduction reaction electrocatalyst subjected to activation and degradation protocols. By scaling up identical-location STEM to hundreds of nanoparticles, we move beyond the small sample sizes of previous studies and achieve statistically meaningful insights. Dissolution was the dominant process, followed by unchanged particles and detachments, while agglomeration, attachment, and deposition were far less frequent. The analysis also revealed clear size- and shape-dependent trends, with smaller and irregular particles being more susceptible to degradation.

To enable large-scale IL-STEM, we established a three-step image analysis workflow comprising segmentation, tracking, and degradation-event classification. Automation, including the use of machine learning, reduced manual effort while preserving accuracy, though manual inspection remains useful for small datasets or rare events. Despite not perfectly reproducing the manually annotated ground truth data, the functional automated pipeline provides adequate insights into the frequency of individual degradation mechanisms without a prohibitive time investment. The microscopy results were consistent with electrochemical measurements, while theoretical models provided further mechanistic context.

This work demonstrates that scaled-up IL-STEM, combined with automated analysis, enables quantitative and unbiased mapping of nanoparticle degradation pathways. Such statistically grounded insights cannot be obtained from random-location imaging or small-scale IL-STEM. This work establishes a transferable workflow for statistically robust degradation tracking, a methodology that can be adapted to other catalyst systems, including those with higher metal loadings relevant to heavy-duty applications. While this study focuses on individual nanoparticle events, an even deeper understanding of collective degradation will require 3D information and tracking of support changes. In the long term, extending automation to the imaging process itself could realize fully autonomous, closed-loop microscopy, accelerating the discovery of durable fuel cell electrocatalysts.

## Methods

### Material

A Pt-Co/C reference electrocatalyst was purchased from FuelCellStore^58^. The catalyst was described as Pt-Co alloy nanoparticles with an average size of 2-3 nanometers on Vulcan XC-72 carbon support. The total metal loading was specified as 20 wt.% with a 1:1 Pt: Co molar ratio.

### Powder X-ray diffraction

XRD patterns were collected using a PANalytical X’Pert PRO MPD diffractometer with Cu Kα_1_ radiation (*λ* = 1.5406 Å) in the 2*θ* range from 10° to 80° with a step size of 0.034° and a holding time of 850 s. Four scans were collected under the same instrumental conditions and summed up. Samples were prepared on a Fe holder.

### Scanning transmission electron microscopy

STEM imaging was performed in a probe Cs-corrected scanning transmission electron microscope Jeol ARM 200 CF. The accelerating voltage was 80 kV, and the convergence semi-angle was set to ~18 mrad. The collection semi-angles for the bright-field and high-angle annular dark-field images were 0-45 and 68-185 mrad, respectively.

Energy-dispersive X-ray spectrometry was performed using an SDD Jeol Centurio spectrometer. Identical location EDX maps were acquired using a resolution of 128×128 pixels with a 0.1 ms dwell time and limiting them to 250 sweeps each in areas of ~38 nm^2^ and ~48 nm^2^. The combination of spot size and condenser aperture gave a STEM probe current of 48.9 pA. These parameters were chosen as a compromise to avoid large modifications in the material due to long beam exposure, but at the same time retrieve enough X-ray counts for reliable maps.

A 1 mg mL^-1^ suspension of the Pt-Co/C electrocatalyst was prepared with Milli-Q water (18.2 MΩ cm). 1.5 µL of the suspension was dropcasted on a gold holey-carbon-coated TEM grid (Agar Scientific) and dried at room temperature (RT; approx. 22 °C). The geometric area of the grid was 0.031425 cm^2^. A total catalyst amount of 1.5 µg was deposited in the center of the grid, forming a spot approximately 1 mm in diameter (0.785 mm^2^).

Identical-location imaging involved collecting STEM data from the same locations both before and after sample treatment. Several spots were identified and imaged at various magnifications to assist in tracking them. All images were captured under identical instrumental conditions.

### Sample treatment

Sample treatment, aimed at introducing structural changes to the Pt-Co/C electrocatalyst, was carried out using a modified floating electrode setup. The MFE setup was chosen to bridge the gap between model and realistic systems, as it mitigates the mass transport limitations and allows for higher current densities compared to a rotating disk electrode, while maintaining the sample geometry required for IL-STEM analysis. The setup featured a two-piece Teflon housing with a metallic spring positioned between two metallic cones. A gas diffusion layer (thickness 280 μm) with 40% Teflon weight wet proofing (Toray Carbon Paper 090, FuelCellStore), was used as a separator between the metallic cones and the TEM grid. The TEM grid assumed the role of the working electrode.

MFE experiments were done in a three-electrode setup with 0.1 M HClO_4_ (70 % Rotipuran Supra, Carl Roth, diluted by Milli-Q water, 18.2 MΩ cm, resulting in a nominal pH of approx. 1) as the electrolyte. After preparation, the electrolyte was stored in FEP bottles for no more than a week. The volume of the electrolyte in the cell was approx. 20 mL. A reversible hydrogen electrode (HydroFlex, Gaskatel) was used as the reference electrode, and a Pt mesh (99.99%, Goodfellow) as the counter electrode. The reference electrode was calibrated against a reversible hydrogen electrode, consisting of a Pt wire in the electrolyte solution used for the measurements, purged thoroughly with hydrogen gas (Messer, 5.0). A gas tube was inserted in the holes in the Teflon housing to purge the system with the chosen gas.

The electrochemical protocol was carried out with a BioLogic SP-200 potentiostat. First, the system was purged with argon (Messer, 6.0). The catalyst was activated with 200 cyclic voltammograms between 0.05 and 1.2 V vs. RHE at a scan rate of 300 mV s^-1^, followed by degradation with 5000 cycles between 0.4 and 1.2 V vs. RHE at 1 V s^-1^. Finally, the grid was washed with Milli-Q water and dried at RT. The uncompensated resistance for this measurement system is typically between 100 and 130 Ω. The voltage was not iR-corrected.

The same protocol was also carried out with a PalmSens EmStat4X potentiostat and a Pt wire (99.99%, Goodfellow) as a counter electrode on an identically prepared sample to investigate the reproducibility of the measurements and mitigate external effects on the signal.

Data were collected using EC-Lab software for experiments conducted with the BioLogic potentiostat, and PSTrace software for experiments conducted with the PalmSens potentiostat. Origin software was used to inspect the raw data.

### Image analysis

For a detailed analysis, images at specific magnifications were selected based on the relationship between pixel size and the average nanoparticle size. In the activation dataset, pixel sizes from 0.022 to 0.061 nm were considered. In the degradation dataset, all analyzed images were collected with a pixel size of 0.036 nm. Nanoparticles in IL-STEM images were annotated manually to produce particle masks using the Supervisely platform^59^. Each mask was defined by two geometric parameters: equivalent sphere diameter and circularity, both calculated from the measured areas and perimeters. The equivalent sphere diameter was determined as:

1 $${{{\rm{diameter}}}}=\frac{{{{\rm{perimeter}}}}}{\pi }$$

Circularity was defined as:

2 $${{{\rm{circularity}}}}=4\pi \left(\tfrac{{{{\rm{area}}}}}{{{{{\rm{perimeter}}}}}^{2}}\right)$$

Particle masks in IL-STEM images were connected to form events, allowing us to track each annotated nanoparticle. Only particles that did not touch the edges of the images were considered. To automate particle association, a heuristic algorithm was developed to align two sets of masks and then connect them based on their proximity and similarity.

These events were then classified into distinct degradation mechanisms using a decision tree. Equivalent sphere diameters of particle masks were converted to real-space quantities using image calibrations. The code for analyzing particle masks was written in Python using open-source libraries.

To automate particle detection in raw images, a neural network was considered, which received BF- and HAADF-STEM images and returned regressed values of particle radii and circularities. A Center-Direction Regression Network (CeDiRNet) for point-based object detection was utilized^60^. In this framework, particle centers are identified by regressing, at each pixel, a vector pointing to the nearest center rather than classifying pixels as foreground or background. Combining these vectors enables robust localization by leveraging spatial context across the image and reduces the class imbalance inherent in pixel-wise classification. The network was further extended for regressing particle radius and circularity as a dense map prediction following a similar extension for orientations in CeDiRNet-3DoF^61^. Model performance was quantified using standard localization metrics and error measures for particle radius and circularity. A detailed description of the methodology, training, evaluation, and validation metrics can be found in the Supplementary Note 2.

Pairs of manually annotated BF- and HAADF-STEM images of the same regions of interest were used as channels to construct two-channel images. These images served for training and testing the neural network. We utilized 12 pairs of images depicting carbon-supported platinum-nickel nanoparticles, which were taken of a material used in a previous study^62^. We also included several pairs of images of the commercial Pt-Co/C electrocatalyst. However, these were excluded from the final analysis to avoid using the same nanoparticles for both training and analysis. In total, 23 pairs of images were used.

Whenever the resulting detections were used for subsequent analysis, particle binary masks were constructed with a size matching the original size of STEM images, and results were calibrated in real space.

### Modeling

A mathematical model of catalyst particle degradation was used to explain the processes involved in the changes observed during activation. The model is based on previously developed models, describing the changes in the size distribution of catalyst particles^63–65^, modified to explain the evolution of individual particles. Activation was described using the same parameters as were used experimentally.

The model describes two primary degradation mechanisms: particle growth or shrinkage due to dissolution and redeposition, and particle detachment, migration, and agglomeration due to corrosion of the carbon support.

Electrochemical dissolution and redeposition of the catalyst from the particles were modeled using the Butler-Volmer equation, with the effect of particle size incorporated as a Kelvin term in the equilibrium potential^66^. The reaction rate is primarily determined by the electrical potential applied to the catalyst. Diffusion of dissolved catalyst ions between particles was modeled using a finite volume method, with regions of space for each particle defined by a Voronoi diagram^67^.

Corrosion of carbon support results from electrochemical reactions between surface oxides on the catalyst and carbon support, which were also modeled using Butler-Volmer equations. The corrosion rate is used to determine the change in contact surface and, consequently, the adhesion force between catalyst particles and carbon support, which counteracts the attractive forces between particles. Once the contact surface is sufficiently reduced, the particle is free to move in the direction of the net force and potentially agglomerate with another particle.

Degradation processes were described as a set of differential equations, describing the size and 3 spatial coordinates of each particle, as well as oxidation of its surface (surface coverage of hydroxide and oxide groups), oxidation of carbon surface in its vicinity (surface coverage of hydroxide and oxide groups), concentration of dissolved catalyst ion in its vicinity, and contact surface between particle and carbon support, resulting in total of 10 differential equations for each modeled particle.

Equations were solved in the Python programming language using the routine solve_ivp from the Scipy package^68^. Details of the modeling procedure can be found in the Supplementary Note 3.

## Supplementary information

Supplementary Information

Transparent Peer Review file

## Source data

Source Data

## Data Availability

The XRD pattern, MFE measurement data, ex-situ and identical-location STEM images generated in this study have been deposited in the Zenodo repository at https://doi.org/10.5281/zenodo.17158368. Source data are provided with this paper.

## Code availability

The code to analyze IL-STEM data is available at https://doi.org/10.5281/zenodo.17158368.
