# Supplementary material for: Large-scale identical-location electron microscopy enables quantifying nanoparticulate electrocatalyst degradation
Source: Nat Commun. 2026 Oct 10;17:11512. doi: 10.1038/s41467-026-78329-8 (PMC13745521; doi:10.1038/s41467-026-78329-8)
Supplement: Supplementary file 1 [file 41467_2026_78329_MOESM1_ESM.pdf]

Large-scale identical-location electron microscopy enables quantifying nanoparticulate electrocatalyst degradation

SUPPLEMENTARY INFORMATION

Ana Rebeka Kamšek[1,2,3,*], Francisco Ruiz-Zepeda[1,3], Domen Tabernik[4], Jan Vidergar[1,2], Anja Logar[1,5], Ambrož Kregar[6], Danijel Skočaj[4], Goran Dražić[1], Nejc Hodnik[1,3,5,*]

[1] Department of Materials Chemistry, National Institute of Chemistry, Hajdrihova 19, 1000 Ljubljana, Slovenia

[2] Faculty of Chemistry and Chemical Technology, University of Ljubljana, Večna pot 113, 1000 Ljubljana, Slovenia

[3] Institute of Metals and Technology, Lepi pot 11, 1000 Ljubljana, Slovenia

[4] Faculty of Computer and Information Science, University of Ljubljana, Večna pot 113, 1000 Ljubljana, Slovenia

[5] University of Nova Gorica, Vipavska 13, 5000 Nova Gorica, Slovenia

[6] Faculty of Mechanical Engineering, University of Ljubljana, Aškerčeva 6, 1000 Ljubljana, Slovenia

Corresponding authors: ana.rebeka.kamsek@imt.si, nejc.hodnik@ki.si

SI 1. Structural characterization of the investigated Pt-Co/C sample

SI 2. Analyzing the activation and degradation datasets by mechanism

SI 3. Cyclic voltammograms for activation and degradation

SI 4. Dataset size effect

SI 5. Three-step identical-location imaging

SI 6. Thin-film rotating disk electrode and energy-dispersive X-ray spectrometry investigations of the Pt-Co/C sample

SI 7. Analyzing datasets with automated particle detection and association

SI 8. Modeling metal dissolution and carbon corrosion on model nanoparticles

SI 1. Structural characterization of the investigated Pt-Co/C sample

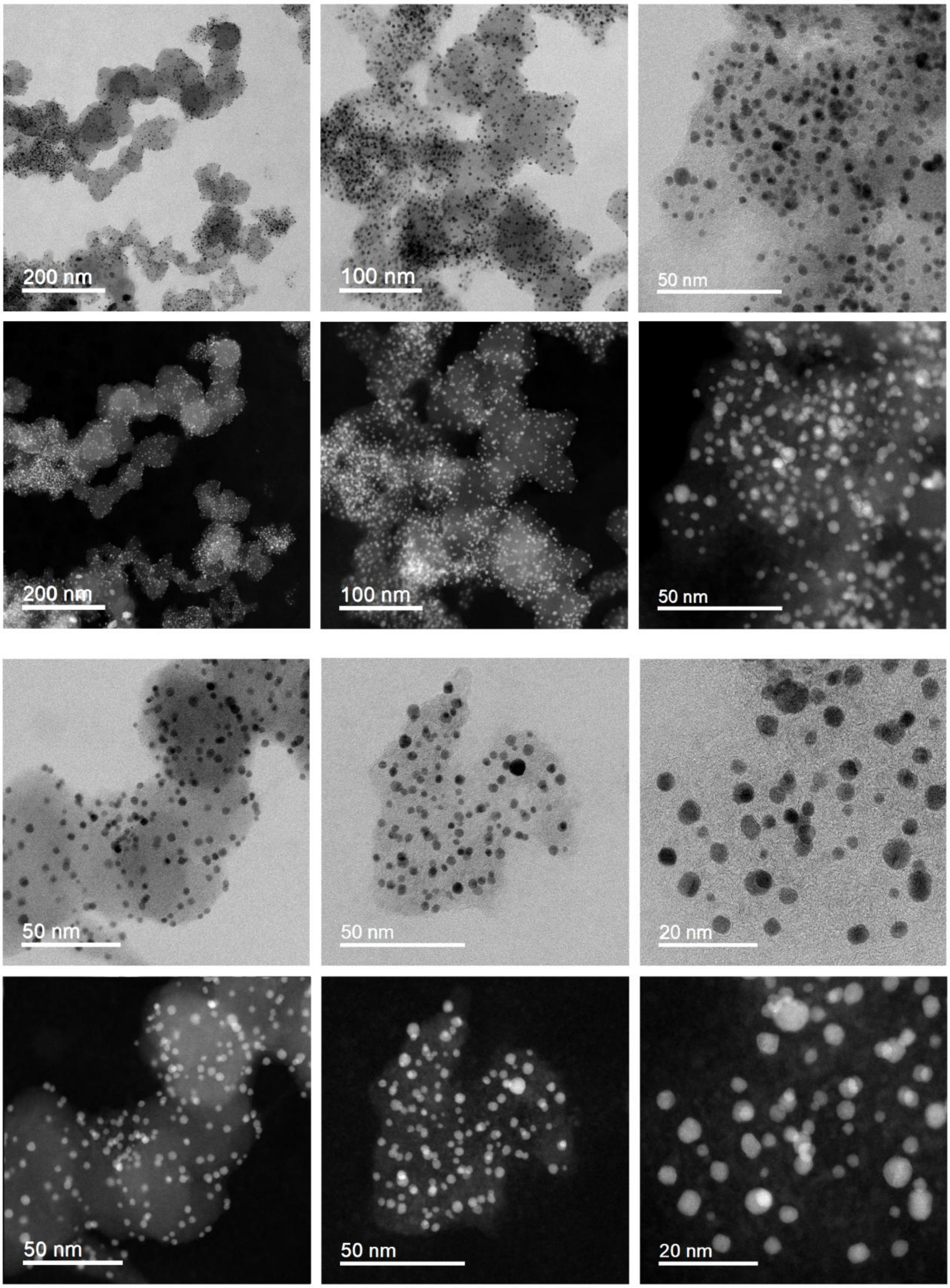

Supplementary Fig. 1. Scanning transmission electron microscopy (STEM) images. Bright-field (BF)-(top) and high-angle annular dark-field (HAADF)-STEM (bottom) images of the Pt-Co/C electrocatalyst in its initial state at various magnifications.

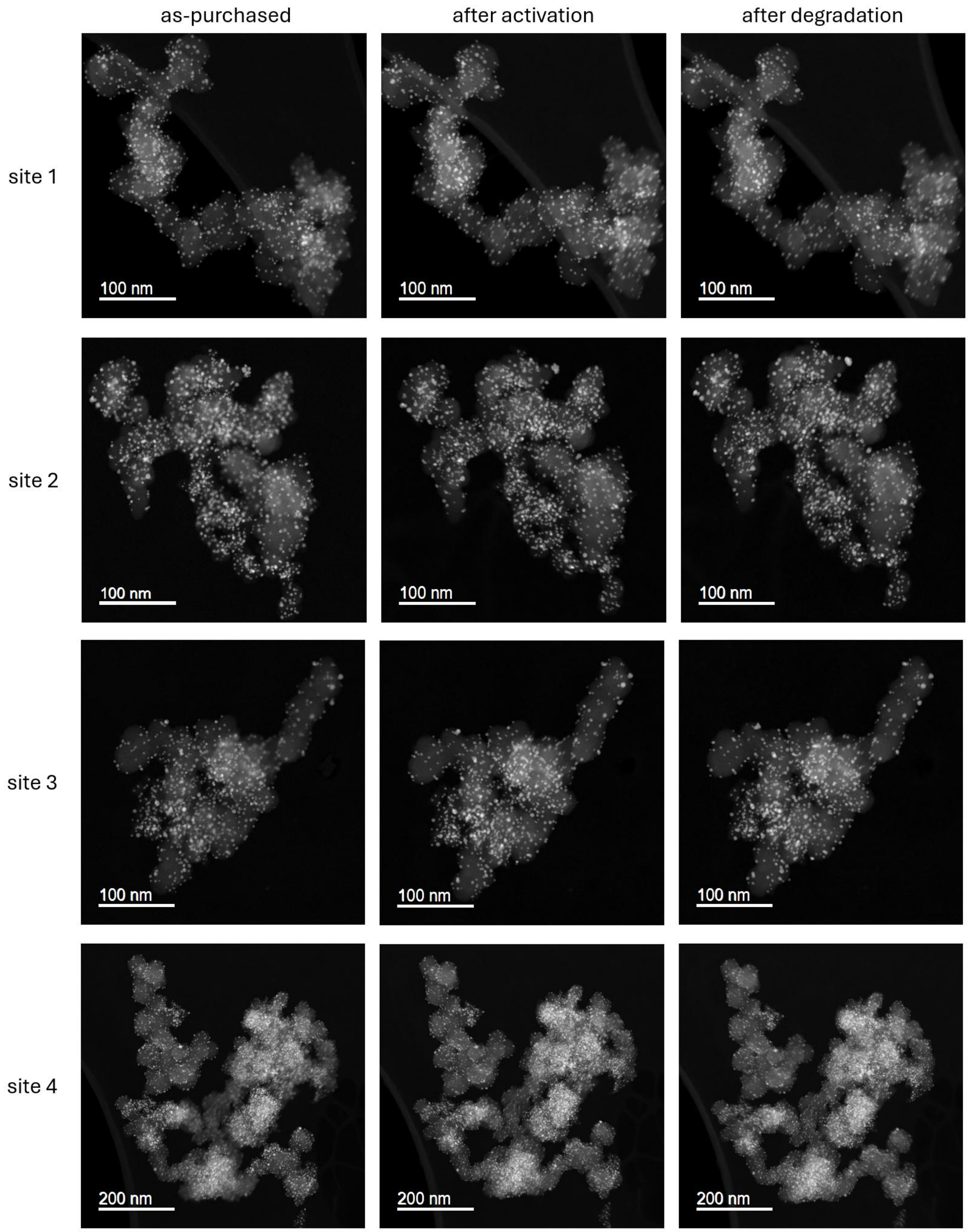

Supplementary Fig. 2. HAADF-STEM images at a lower magnification. The Pt-Co/C sample is imaged in the as-purchased state, after potential cycling activation (200 cycles, 0.05–1.2 V_{RHE} (versus the reversible hydrogen electrode, RHE), 300 mV s^{-1}, 0.1 M $HClO_4$), and after degradation (5000 cycles, 0.4–1.2 V_{RHE}, 1 V s^{-1}, 0.1 M $HClO_4$).

Supplementary Table 1. Energy-dispersive X-ray spectrometry (EDX)-STEM investigation of four catalyst regions from Supplementary Fig. 2 in the as-purchased state, after potential cycling activation (200 cycles, 0.05–1.2 V_{RHE}, 300 mV s^{-1}, 0.1 M $HClO_4$), and after degradation (5000 cycles, 0.4–1.2 V_{RHE}, 1 V s^{-1}, 0.1 M $HClO_4$).

site no.	element	as-purchased [at.%]	after activation [at.%]	after degradation [at.%]
**1**	Co K	47.3	63.7	58.3
	Pt L	52.3	36.3	41.7
**2**	Co K	55.3	39.9	32.0
	Pt L	44.7	60.0	68.0
**3**	Co K	54.9	54.8	54.2
	Pt L	45.1	45.2	45.8
**4**	Co K	45.6	34.7	42.5
	Pt L	54.4	65.4	57.5
**average**	Co K	50.8	48.3	46.7
	Pt L	49.2	51.7	53.3

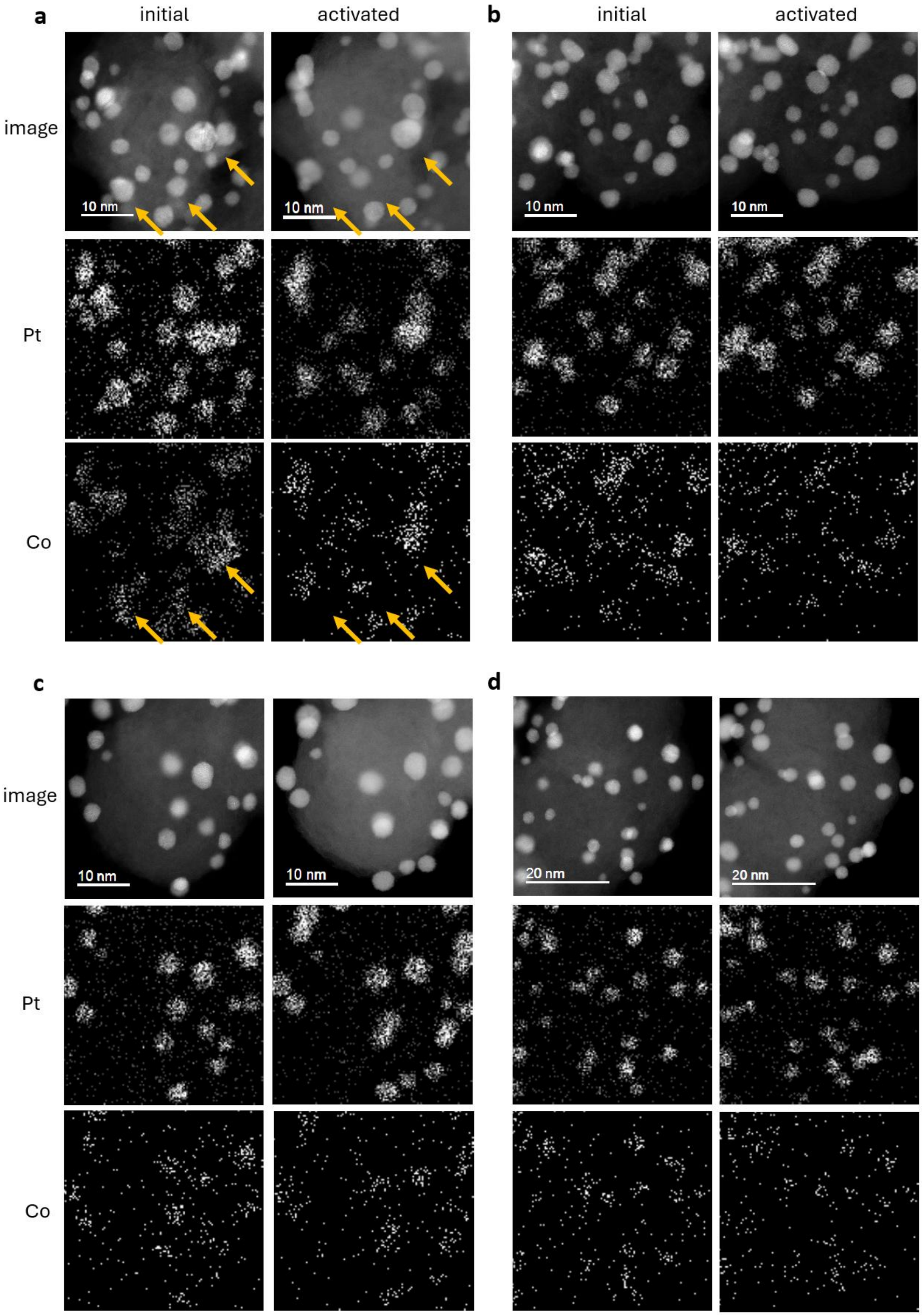

Supplementary Fig. 3. Identical-location STEM and EDX mapping of Pt-Co/C. The panels **a-d** include four regions of interest with HAADF-STEM images and Pt and Co elemental maps in the as-purchased state and after potential cycling activation (200 cycles, 0.05–1.2 V_{RHE}, 300 mV s^{-1}, 0.1 M $HClO_4$). Yellow arrows in **a** denote Co-rich sites in the initial state that are not apparent after activation.

Supplementary Table 2. IL-EDX-STEM investigation of four catalyst regions from Supplementary Fig. 3 in the as-purchased state and after potential cycling activation (200 cycles, 0.05–1.2 V_{RHE}, 300 mV s^{-1}, 0.1 M $HClO_4$).

site no.	element	as-purchased [at.%]	after activation [at.%]
**1**	Co K	51.5	23.9
	Pt M	48.5	76.1
**2**	Co K	30.5	15.8
	Pt M	69.5	84.2
**3**	Co K	20.6	14.1
	Pt M	79.4	85.9
**4**	Co K	19.6	14.0
	Pt M	80.4	86.0

SI 2. Analyzing the activation and degradation datasets by mechanism

Supplementary Table 3. Average values and standard deviations of equivalent sphere diameters and circularities of Pt-Co nanoparticles, determined for each mechanism during potential cycling activation (200 cycles, 0.05–1.2 V_{RHE}, 300 mV s^{-1}, 4 M $HClO_4$).

	**diameter, before [nm]**	**circularity, before []**	**diameter, after [nm]**	**circularity, after []**	**number of events**
**dissolution**	3.99 ± 0.84	0.87 ± 0.03	3.40 ± 0.89	0.88 ± 0.02	106
**no changes**	3.75 ± 0.82	0.87 ± 0.02	3.71 ± 0.82	0.87 ± 0.02	46
**detachment**	2.80 ± 0.77	0.81 ± 0.12			24
**attachment**			2.63 ± 1.02	0.81 ± 0.12	13
**agglomeration**	3.96 ± 0.76	0.87 ± 0.02	5.02 ± 1.63	0.79 ± 0.12	11
**deposition**	3.24 ± 1.00	0.87 ± 0.02	3.60 ± 1.01	0.88 ± 0.02	9

Supplementary Table 4. Average values and standard deviations of equivalent sphere diameters and circularities of Pt-Co nanoparticles, determined for each mechanism during potential cycling activation (200 cycles, 0.05–1.2 V_{RHE}, 300 mV s^{-1}, 0.1 M $HClO_4$), followed by degradation (5000 cycles, 0.4–1.2 V_{RHE}, 1 V s^{-1}, 0.1 M $HClO_4$).

	**diameter, before [nm]**	**circularity, before []**	**diameter, after [nm]**	**circularity, after []**	**number of events**
**dissolution**	3.67 ± 0.79	0.88 ± 0.02	3.02 ± 1.03	0.87 ± 0.02	235
**no changes**	3.82 ± 0.87	0.88 ± 0.01	3.81 ± 0.87	0.87 ± 0.02	214
**detachment**	2.59 ± 0.59	0.84 ± 0.11			131
**deposition**	3.51 ± 0.92	0.88 ± 0.02	3.91 ± 0.96	0.87 ± 0.02	58
**attachment**			2.41 ± 0.75	0.65 ± 0.15	38
**agglomeration**	4.07 ± 0.75	0.88 ± 0.01	4.43 ± 1.78	0.83 ± 0.08	12
**Ostwald ripening**	3.62 ± 0.43	0.89 ± 0.01	3.96 ± 0.49	0.88 ± 0.01	5
**complete Ostwald ripening**	3.29 ± 2.35	0.89 ± 0.01	3.81 ± 2.03	0.84 ± 0.05	3

SI 3. Cyclic voltammograms for activation and degradation

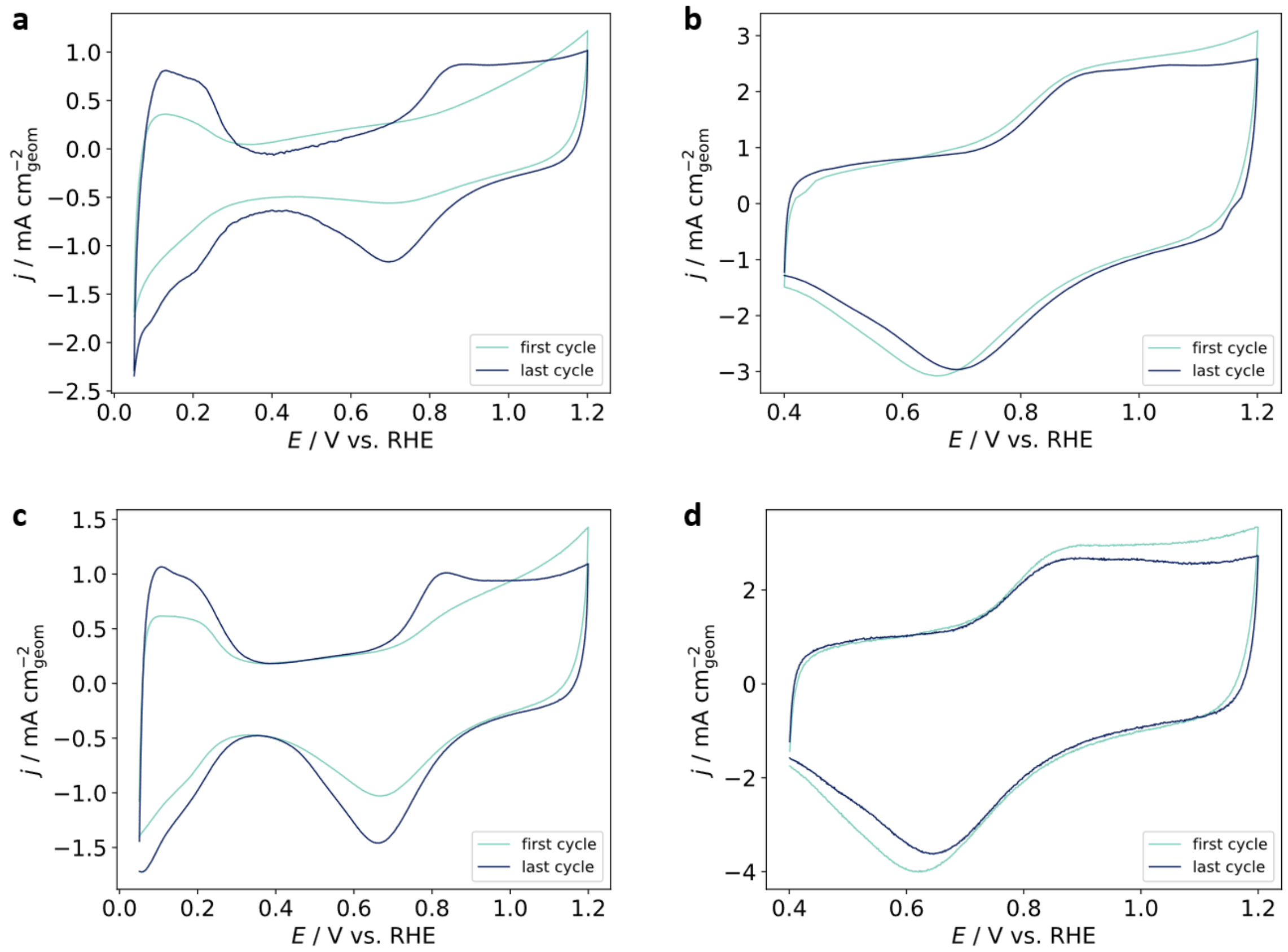

Supplementary Fig. 4. Cyclic voltammetry results, collected with a modified floating electrode setup. The first and last cycles of the **(a, c)** potential cycling activation (200 cycles, 0.05–1.2 V_{RHE}, 300 mV s^{-1}, 0.1 M $HClO_4$) and **(b, d)** degradation (5000 cycles, 0.4–1.2 V_{RHE}, 1 V s^{-1}, 0.1 M $HClO_4$) of the Pt-Co/C. Currents were normalized to the geometric area of the electrode (0.031425 cm^2). The voltage was not iR-corrected. Source data are provided as a Source Data file.

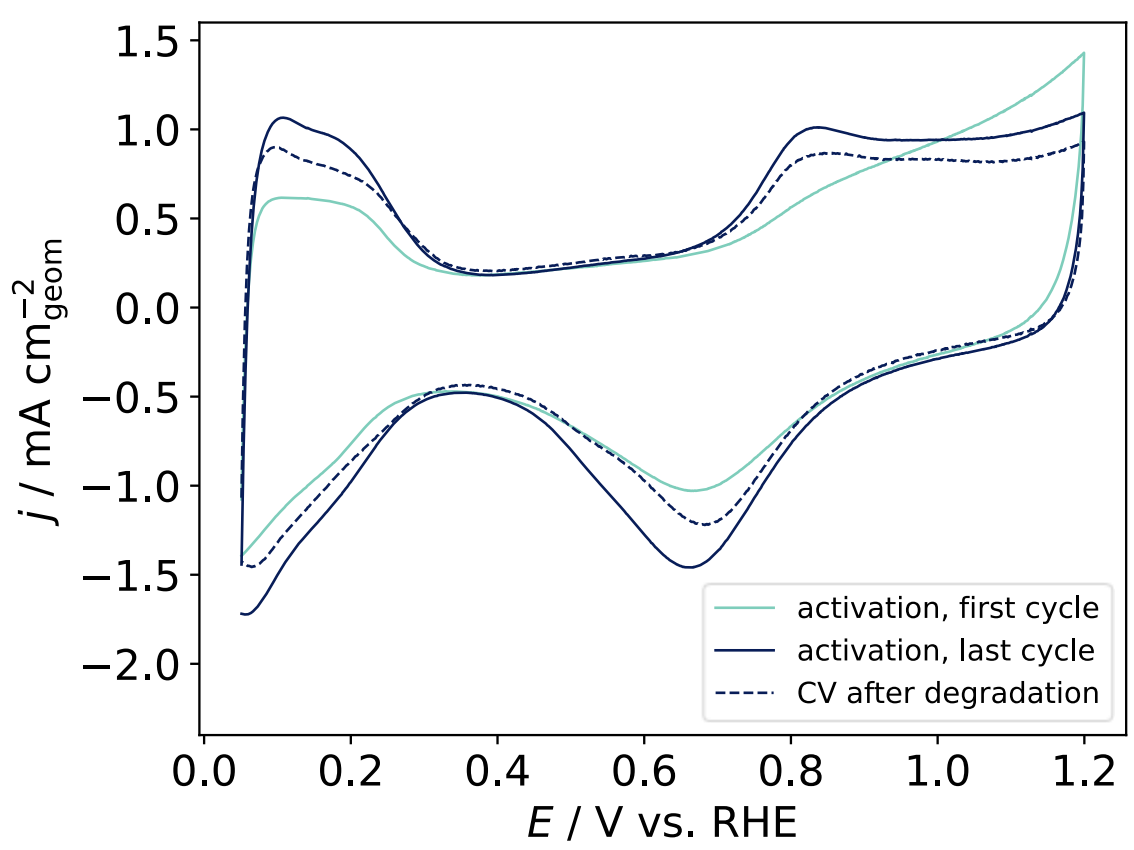

Supplementary Fig. 5. Cyclic voltammetry comparison. A full-range cyclic voltammogram, recorded after degradation, compared to the first and last cycles of the potential cycling activation (all 0.05–1.2 V_{RHE}, 300 mV s^{-1}, 0.1 M $HClO_4$) of the Pt-Co/C. Currents were normalized to the geometric area of the electrode (0.031425 cm^2). The voltage was not iR-corrected. Source data are provided as a Source Data file.

SI 4. Dataset size effect

Supplementary Note 1: Analyzing the effect of sample size on particle size and circularity

To investigate how the reliability of the determined particle sizes and circularities changes with sample size, random subsets of increasing size (25 to 600 particles) were repeatedly drawn without replacement from the manually annotated degradation dataset for the initial state of the Pt-Co/C electrocatalyst. 100 subsets were drawn at each subset size, and the mean particle diameter and circularity were computed. At a given subset size, the standard deviation of the resulting distribution of subset means represents the standard error of the mean, which serves as an estimate of how much the reported mean would be expected to vary had data collection been stopped at that number of particles. To compare this effect on diameters and circularities, which differ in scale and units, the standard error of the mean was also expressed as a percentage of the corresponding mean. The results are summarized in Supplementary Fig. 6. This relative error for circularity remains below 1% across the entire range of subset sizes. The relative error for particle diameter is initially higher, reflecting the broader spread of particle sizes in the ensemble, but falls below 2% for subset sizes greater than 150 particles and below 1% for subset sizes greater than 400 particles. Both metrics therefore converge to stable values well within the range of dataset sizes used in this and related studies, including the ones that similarly investigated the effect of subset size on a desired metric.[1,2] This confirms that a dataset of several hundred particles is statistically sufficient to characterize the distribution of this catalyst system.

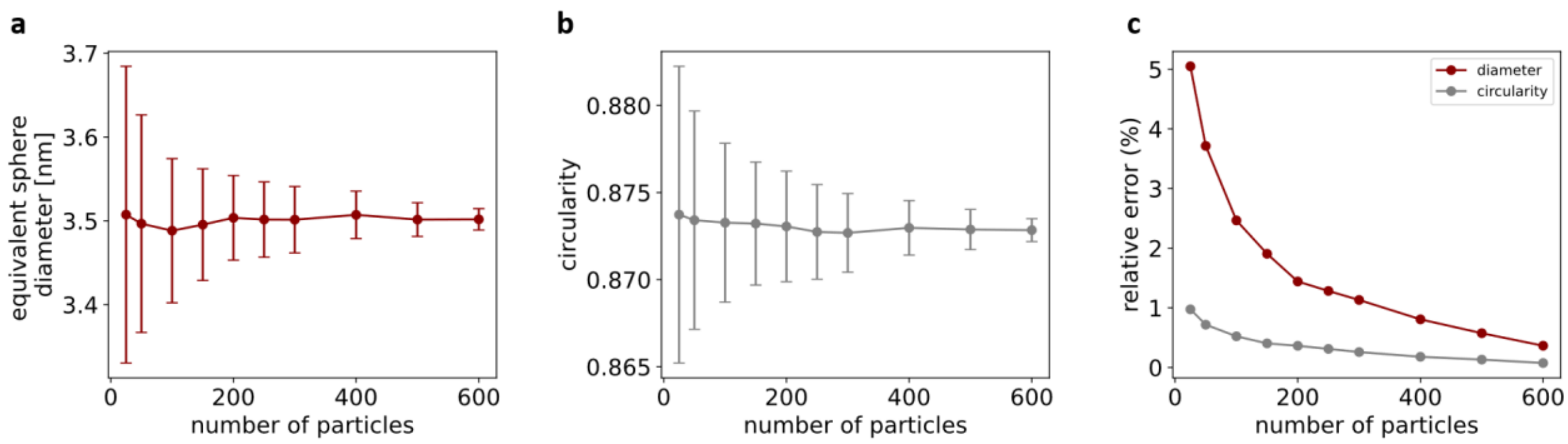

Supplementary Fig. 6. Convergence of mean particle diameter and circularity with sample size. Mean equivalent sphere diameter (**a**) and circularity (**b**) with error bars denoting the standard error of the mean, and the relative standard error of the mean for both metrics (**c**), as a function of the number of randomly sampled particles (25-600, 100 subsets per size, sampled without replacement). Source data are provided as a Source Data file.

SI 5. Three-step identical-location imaging

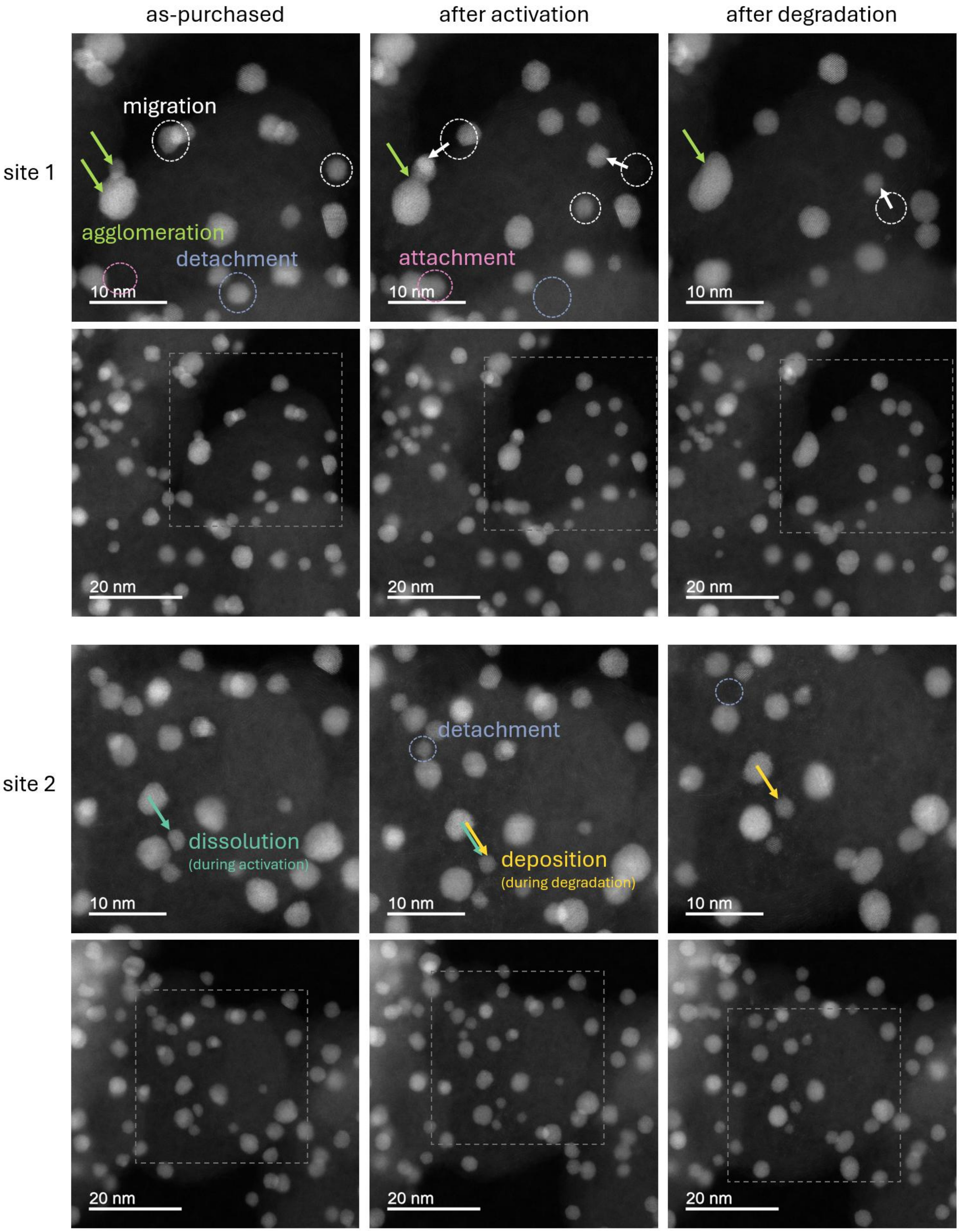

Supplementary Fig. 7. Three-step IL-HAADF-STEM of Pt-Co/C. Two regions of interest were imaged in their as-purchased state, after potential cycling activation (200 cycles, 0.05–1.2 V_{RHE}, 300 mV s^{-1}, 0.1 M $HClO_4$), and after degradation (5000 cycles, 0.4–1.2 V_{RHE}, 1 V s^{-1}, 0.1 M $HClO_4$). The top images show a close-up of the region with annotated specific degradation phenomena, and the bottom images are shown at lower magnification for context.

SI 6. Thin-film rotating disk electrode and energy-dispersive X-ray spectrometry investigations of the Pt-Co/C sample

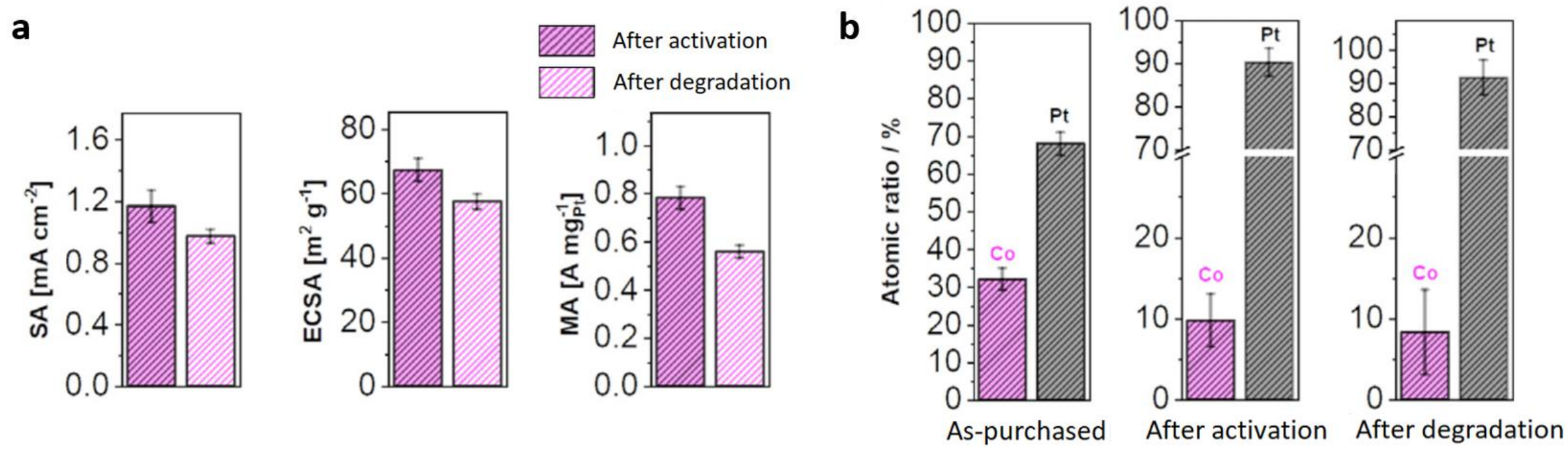

Supplementary Fig. 8. RDE and EDX investigation of the Pt-Co/C electrocatalyst. a Specific activity (SA), electrochemically active surface area (ECSA), and mass activity (MA) of the Pt-Co/C after activation (200 cycles, 0.05–1.2 V_{RHE}, 300 mV s^{-1}, 600 rpm, 0.1 M $HClO_4$) and degradation (5000 cycles, 0.4–1.2 V_{RHE}, 1 V s^{-1}, 600 rpm, 0.1 M $HClO_4$), measured with a rotating disk electrode. **b** Ex situ EDX-STEM elemental analysis of the Pt-Co/C in the initial state, after activation, and after degradation. Adapted from [3]. Attribution 4.0 International (CC BY 4.0).

SI 7. Analyzing datasets with automated particle detection and association

Supplementary Note 2: Details on automated particle detection using a neural network

Automatic particle detection was implemented using a Center-Direction Regression Network (CeDiRNet) designed for point-based object detection.[4] The network consists of a two-stage architecture, regressing dense center-directions with a ConvNeXt backbone and a Feature Pyramid Network in the first stage, and a point localization from dense center-direction maps in the second stage.

CeDiRNet performs dense, per-pixel regression of vectors pointing to the nearest object center, enabling robust localization by leveraging a broad spatial context around each center rather than relying on a single-pixel prediction. This avoids learning features from highly unbalanced per-pixel binary classification, where loss is dominated by negative background pixels and only a small set of foreground pixels provides positive signals. Translating the problem into a regression of vectors pointing towards the closest object evenly spreads the learning signal to all pixels, forcing even pixels further away from the target to provide useful information for the detection. The mathematically well-defined appearance of the regressed center-directions renders the localization stage independent of domain-specific image features, allowing the use of a generic pre-trained model trained entirely on synthetic data.

The architecture was implemented in PyTorch 1.9, with CUDA 11.1, using Python 3.8. Code and datasets will be made publicly available at https://github.com/vicoslab/CeDiRNet-STEM.

a) Attribute extension for particle radius and circularity

The first stage of CeDiRNet was further extended to regress particle radius and circularity as a dense map prediction following a similar extension for orientations in CeDiRNet-3DoF.[5] Both attributes were associated with the corresponding center location of the particle, regressing the associated values within 15 pixels of the center during training, while reading the values at the detected particle center during inference.

Circularity value was regressed directly, with values ranging from 0 to 1, while radius was transformed into a logarithmic value to reduce the range of values for regression. Both radius and circularity were trained using L1 together with center-directions, weighing the radius with a factor of 0.25, circularity loss with a factor of 0.5, and applying an additional uncertainty weighting between them and the center-directions loss.[6]

b) Input and learning details

For input, pairs of BF- and HAADF-STEM images were resized to 512x512 pixels, merged into two-channel images, and normalized according to the ImageNet standard. The dense regression network was trained on a single NVIDIA A100 40GB graphics processing unit (GPU) using 23 pairs of images for 1000 epochs using the Adam optimizer with a batch size of 4 and a learning rate of 10^{-4} with polynomial decay using an exponent of 2. During training, images were augmented using random horizontal and vertical flips and color jittering with a factor of 0.3, each applied with a probability of 0.5. During training and evaluation, we excluded partially visible particles near the image borders, as their center positions, sizes, and shapes could not be determined accurately.

The regression stage was initialized with weights from an ImageNet-pretrained ConvNeXt-Base model. The localization stage used a generic network from ref. [4], trained on 16x NVIDIA A100 (40 GB) GPUs for 200 epochs using 512 × 512-pixel synthetic images, with a batch size of 786 and 5,000 unique synthetic samples per epoch, and without any additional training on STEM images.

The model was evaluated using standard localization metrics and absolute and relative error measures for diameter and circularity. Localization performance was assessed using the F1 score, which accounts for both precision and recall. Detections within 20 pixels of the ground-truth location were considered true positives; all others were classified as false positives. The F1 score was computed at the optimal score threshold on the test set. Diameter and circularity errors were evaluated only for true-positive detections. The F1 score was determined to be 96.32%, precision 94.47%, and recall 98.45%.

Supplementary Table 5. Average values and standard deviations of equivalent sphere diameters and circularities of Pt-Co nanoparticles (automated detection, manual association) were determined for each mechanism during potential cycling activation (200 cycles, 0.05–1.2 V_{RHE}, 300 mV s^{-1}, 4 M $HClO_4$).

	diameter, before [nm]	circularity, before []	diameter, after [nm]	circularity, after []	number of events
**dissolution**	3.92 ± 0.83	0.88 ± 0.02	3.40 ± 0.83	0.88 ± 0.02	83
**no changes**	3.88 ± 0.84	0.88 ± 0.02	3.84 ± 0.86	0.88 ± 0.02	54
**deposition**	3.47 ± 0.67	0.86 ± 0.02	3.87 ± 0.76	0.87 ± 0.02	12
**detachment**	2.52 ± 0.70	0.89 ± 0.02			11
**agglomeration**	3.79 ± 0.34	0.89 ± 0.02	4.20 ± 0.22	0.87 ± 0.00	3
**attachment**			1.95 ± 0.47	0.90 ± 0.04	2
**Ostwald ripening**	2.67 ± 0.69	0.87 ± 0.00	3.30 ± 0.23	0.87 ± 0.00	2
**complete Ostwald ripening**	3.70 ± 0.05	0.83 ± 0.00	3.94 ± 0.00	0.84 ± 0.00	1

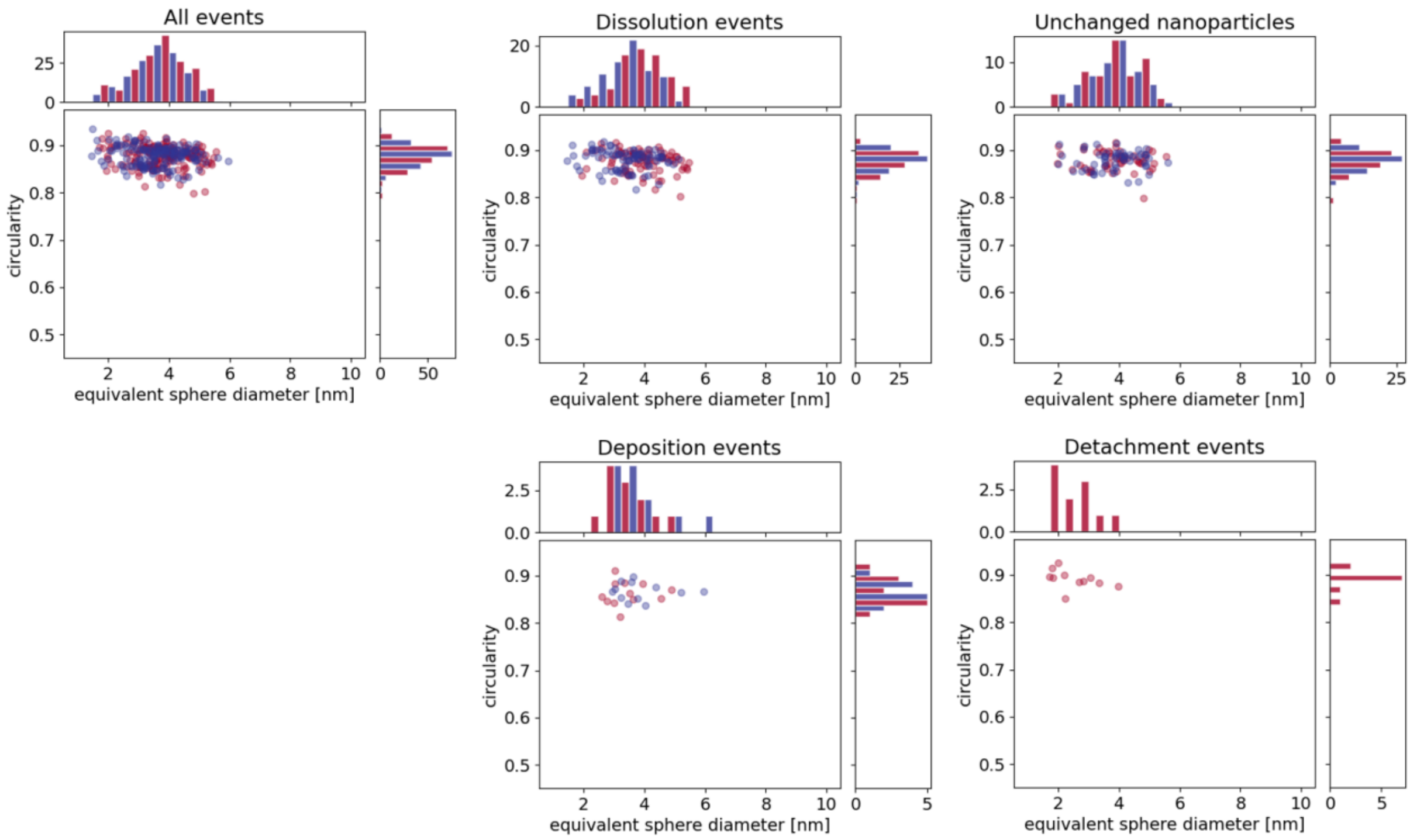

Supplementary Fig. 9. Degradation events for Pt-Co nanoparticles during activation (automated detection, manual association). Scatter plots with histograms showing circularities and equivalent sphere diameters before (red) and after (blue) activation for all events and individual degradation mechanisms (200 cycles, 0.05–1.2 V_{RHE}, 300 mV s^{-1}, 4 M $HClO_4$). Source data are provided as a Source Data file.

Supplementary Table 6. Average values and standard deviations of equivalent sphere diameters and circularities of Pt-Co nanoparticles (automated detection, manual association) determined for each mechanism during potential cycling activation (200 cycles, 0.05–1.2 V_{RHE}, 300 mV s^{-1}, 0.1 M $HClO_4$), followed by degradation (5000 cycles, 0.4–1.2 V_{RHE}, 1 V s^{-1}, 0.1 M $HClO_4$).

	**diameter, before [nm]**	**circularity, before []**	**diameter, after [nm]**	**circularity, after []**	**number of events**
**no changes**	3.64 ± 0.81	0.89 ± 0.01	3.62 ± 0.80	0.90 ± 0.01	175
**dissolution**	3.49 ± 0.75	0.89 ± 0.01	2.94 ± 0.83	0.90 ± 0.01	171
**detachment**	2.47 ± 0.50	0.91 ± 0.01			83
**deposition**	3.24 ± 0.61	0.90 ± 0.01	3.65 ± 0.64	0.89 ± 0.01	67
**agglomeration**	3.92 ± 0.97	0.88 ± 0.01	4.58 ± 0.72	0.86 ± 0.02	8
**attachment**			2.67 ± 0.35	0.91 ± 0.00	3
**Ostwald ripening**	3.99 ± 0.82	0.89 ± 0.01	4.40 ± 0.81	0.89 ± 0.02	3

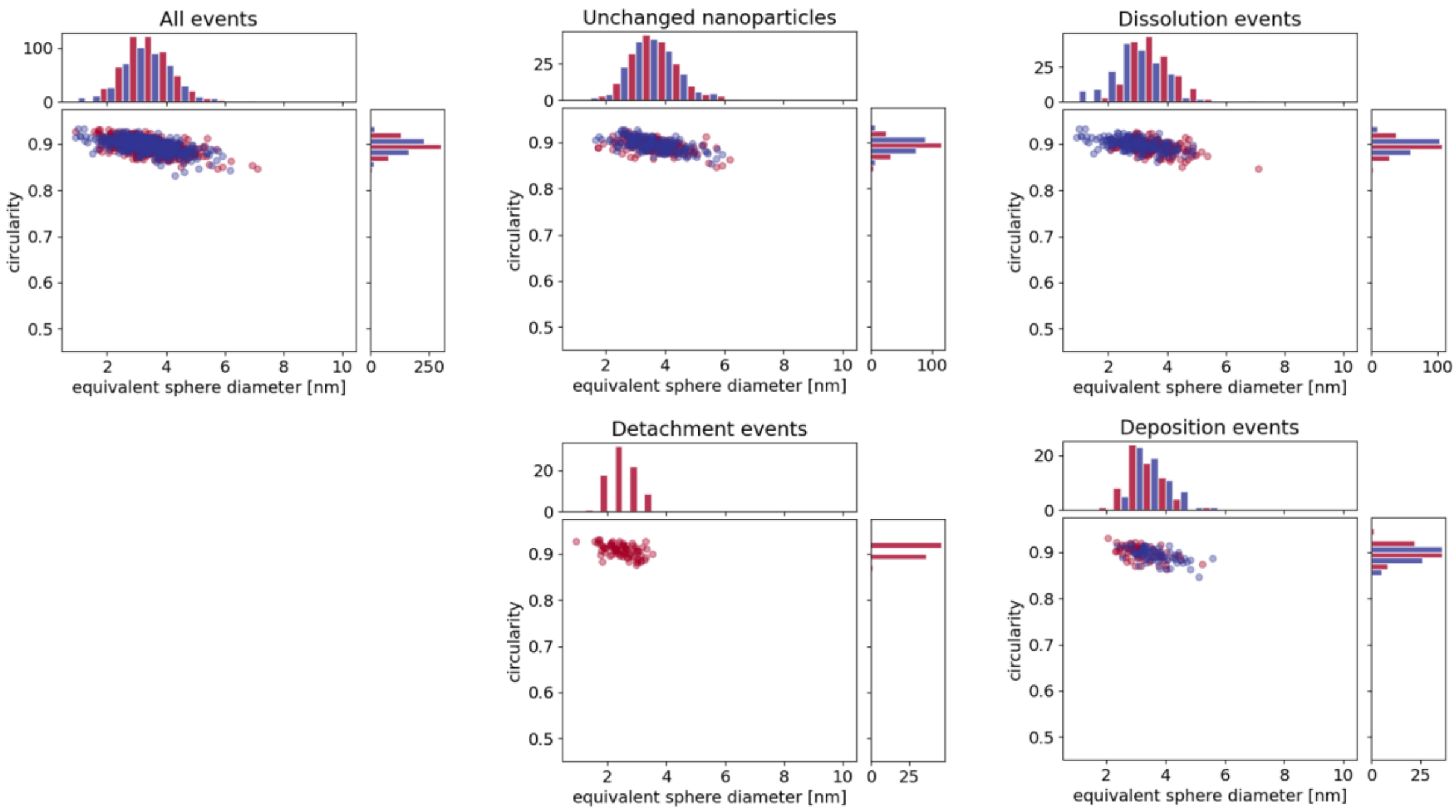

Supplementary Fig. 10. Degradation events for Pt-Co nanoparticles during potential cycling activation, followed by degradation (automated detection, manual association). Scatter plots with histograms showing circularities and equivalent sphere diameters before (red) and after (blue) activation (200 cycles, 0.05–1.2 V_{RHE}, 300 mV s^{-1}, 0.1 M $HClO_4$) and degradation (5000 cycles, 0.4–1.2 V_{RHE}, 1 V s^{-1}, 0.1 M $HClO_4$) for all events and individual degradation mechanisms. Source data are provided as a Source Data file.

Supplementary Table 7. Average values and standard deviations of equivalent sphere diameters and circularities of Pt-Co nanoparticles (manual segmentation, automated association) were determined for each mechanism during potential cycling activation (200 cycles, 0.05–1.2 V_{RHE}, 300 mV s^{-1}, 4 M $HClO_4$).

	**diameter, before [nm]**	**circularity, before []**	**diameter, after [nm]**	**circularity, after []**	**number of events**
**dissolution**	3.98 ± 0.84	0.87 ± 0.03	3.38 ± 0.89	0.87 ± 0.02	96
**no changes**	3.81 ± 0.82	0.87 ± 0.02	3.77 ± 0.81	0.87 ± 0.02	46
**detachment**	2.90 ± 0.87	0.88 ± 0.02			19
**deposition**	3.20 ± 0.81	0.86 ± 0.02	3.85 ± 1.29	0.85 ± 0.08	9
**agglomeration**	3.86 ± 0.81	0.88 ± 0.02	4.52 ± 1.97	0.80 ± 0.12	8
**attachment**			2.95 ± 0.94	0.89 ± 0.02	8
**complete Ostwald ripening**	3.83 ± 0.95	0.86 ± 0.02	3.95 ± 0.72	0.89 ± 0.01	2
**Ostwald ripening**	4.74 ± 0.78	0.88 ± 0.02	5.35 ± 0.67	0.84 ± 0.01	2

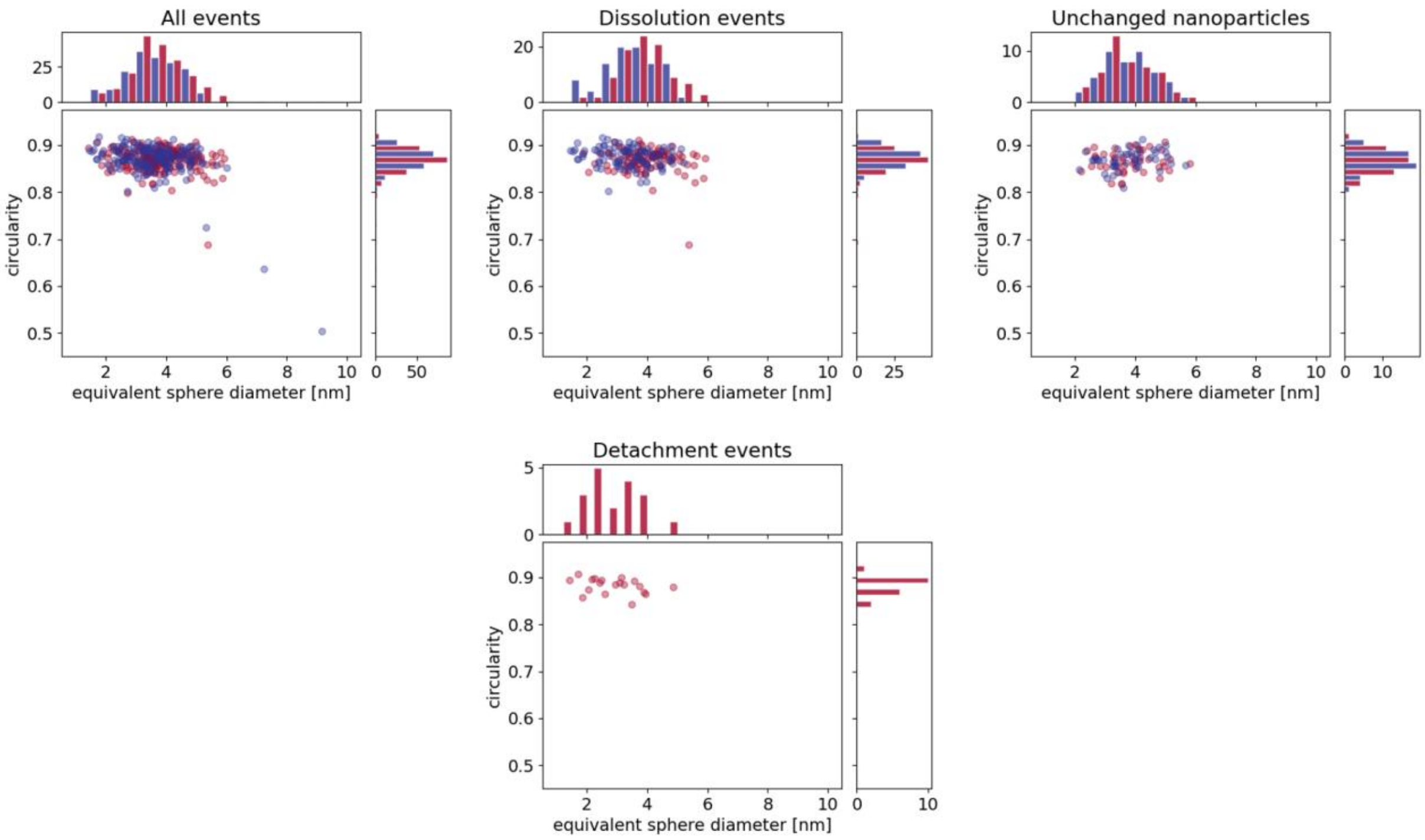

Supplementary Fig. 11. Degradation events for Pt-Co nanoparticles during activation (manual segmentation, automated association). Scatter plots with histograms showing circularities and equivalent sphere diameters before (red) and after (blue) activation for all events and individual degradation mechanisms (200 cycles, 0.05–1.2 V_{RHE}, 300 mV s^{-1}, 4 M $HClO_4$). Source data are provided as a Source Data file.

Supplementary Table 8. Average values and standard deviations of equivalent sphere diameters and circularities of Pt-Co nanoparticles (manual segmentation, automated association) determined for each mechanism during potential cycling activation (200 cycles, 0.05–1.2 V_{RHE}, 300 mV s^{-1}, 0.1 M $HClO_4$), followed by degradation (5000 cycles, 0.4–1.2 V_{RHE}, 1 V s^{-1}, 0.1 M $HClO_4$).

	**diameter, before [nm]**	**circularity, before []**	**diameter, after [nm]**	**circularity, after []**	**number of events**
**dissolution**	3.70 ± 0.79	0.88 ± 0.02	3.03 ± 1.00	0.87 ± 0.02	201
**no changes**	3.75 ± 0.87	0.88 ± 0.01	3.76 ± 0.87	0.87 ± 0.02	190
**detachment**	2.73 ± 0.67	0.88 ± 0.03			112
**deposition**	3.48 ± 0.93	0.88 ± 0.02	4.12 ± 1.18	0.87 ± 0.04	62
**attachment**			2.52 ± 1.06	0.87 ± 0.03	24
**agglomeration**	3.88 ± 0.94	0.88 ± 0.01	3.86 ± 0.92	0.86 ± 0.04	23
**Ostwald ripening**	4.32 ± 1.18	0.89 ± 0.01	4.82 ± 1.41	0.85 ± 0.05	7
**complete Ostwald ripening**	3.86 ± 0.81	0.81 ± 0.02	3.68 ± 0.73	0.88 ± 0.01	5

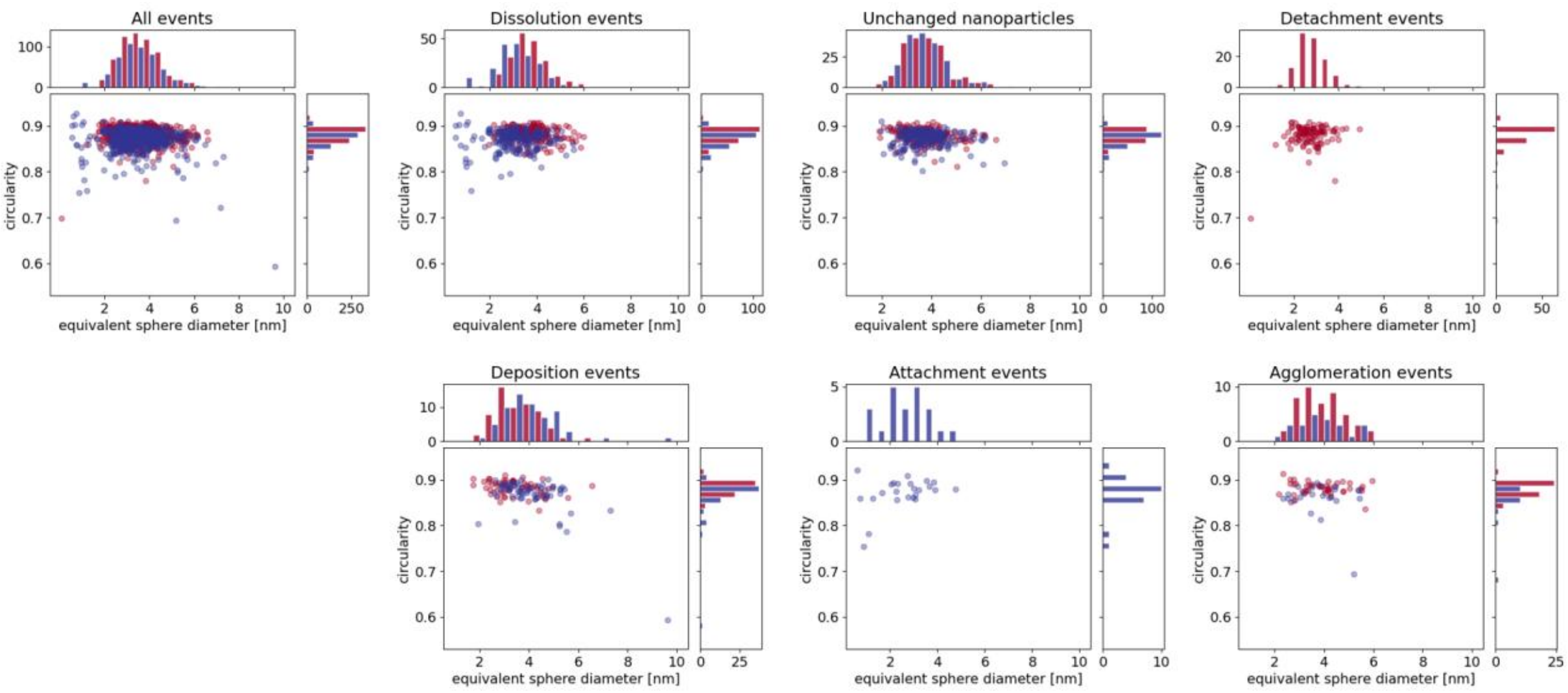

Supplementary Fig. 12. Degradation events for Pt-Co nanoparticles during potential cycling activation, followed by degradation (manual segmentation, automated association). Scatter plots with histograms showing circularities and equivalent sphere diameters before (red) and after (blue) activation (200 cycles, 0.05–1.2 V_{RHE}, 300 mV s^{-1}, 0.1 M $HClO_4$) and degradation (5000 cycles, 0.4–1.2 V_{RHE}, 1 V s^{-1}, 0.1 M $HClO_4$) for all events and individual degradation mechanisms. Source data are provided as a Source Data file.

Supplementary Table 9. Average values and standard deviations of equivalent sphere diameters and circularities of Pt-Co nanoparticles (automated detection, automated association) were determined for each mechanism during potential cycling activation (200 cycles, 0.05–1.2 V_{RHE}, 300 mV s^{-1}, 4 M $HClO_4$).

	**diameter, before [nm]**	**circularity, before []**	**diameter, after [nm]**	**circularity, after []**	**number of events**
**dissolution**	3.95 ± 0.84	0.87 ± 0.02	3.37 ± 0.85	0.88 ± 0.02	88
**no changes**	3.81 ± 0.82	0.88 ± 0.02	3.77 ± 0.81	0.88 ± 0.02	51
**detachment**	2.77 ± 0.72	0.89 ± 0.02			19
**attachment**			3.02 ± 0.94	0.88 ± 0.02	15
**deposition**	2.95 ± 0.63	0.87 ± 0.02	3.50 ± 0.80	0.87 ± 0.01	10
**Ostwald ripening**	3.66 ± 0.55	0.86 ± 0.03	4.08 ± 0.77	0.86 ± 0.02	8
**agglomeration**	3.93 ± 0.18	0.88 ± 0.01	4.30 ± 0.11	0.88 ± 0.01	2
**complete Ostwald ripening**	4.54 ± 0.52	0.86 ± 0.01	2.77 ± 0.00	0.89 ± 0.00	1

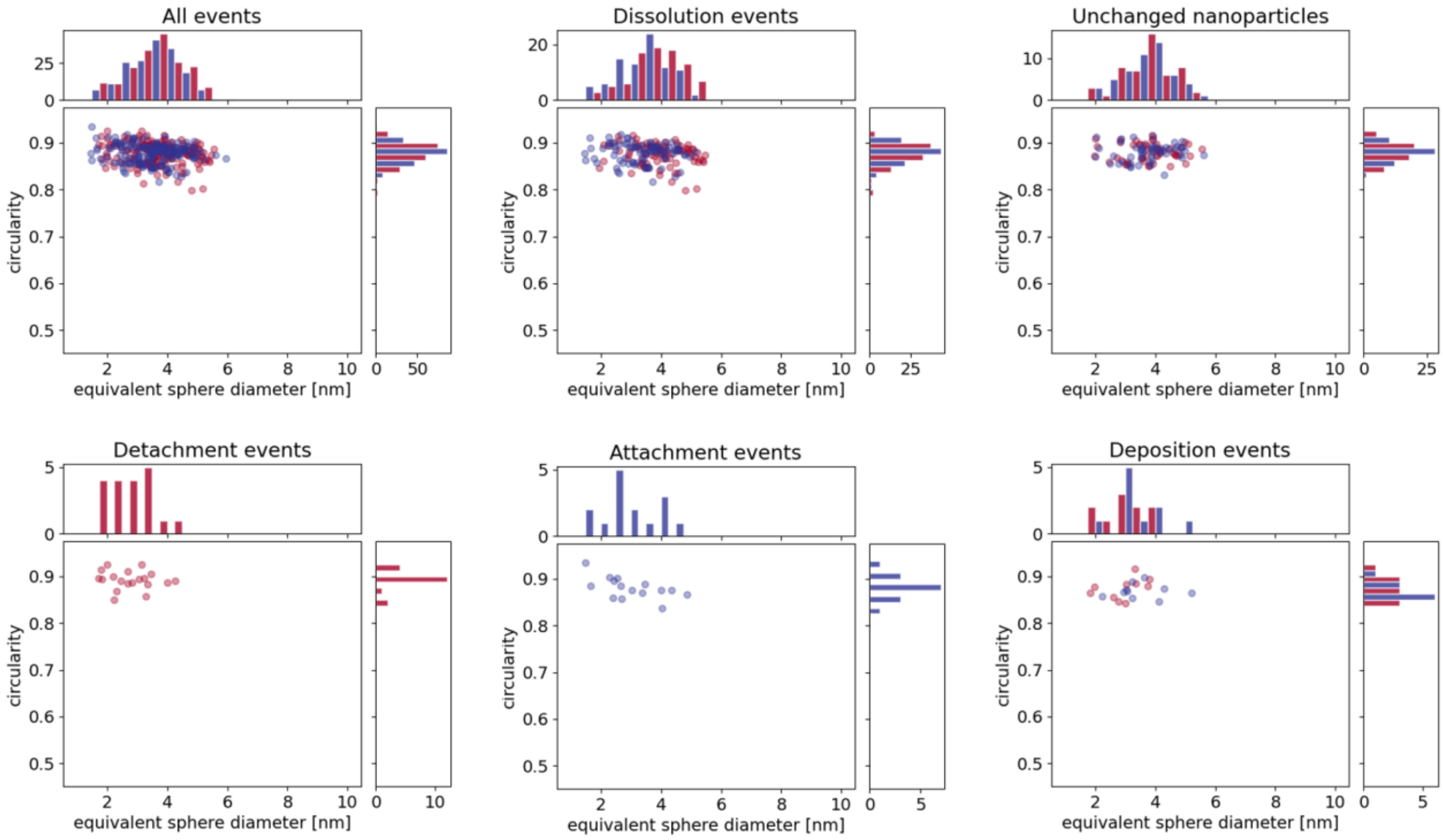

Supplementary Fig. 13. Degradation events for Pt-Co nanoparticles during activation (automated detection, automated association). Scatter plots with histograms showing circularities and equivalent sphere diameters before (red) and after (blue) activation for all events and individual degradation mechanisms (200 cycles, 0.05–1.2 V_{RHE}, 300 mV s^{-1}, 4 M $HClO_4$). Source data are provided as a Source Data file.

Supplementary Table 10. Average values and standard deviations of equivalent sphere diameters and circularities of Pt-Co nanoparticles (automated detection, automated association) determined for each mechanism during potential cycling activation (200 cycles, 0.05–1.2 V_{RHE}, 300 mV s^{-1}, 0.1 M $HClO_4$), followed by degradation (5000 cycles, 0.4–1.2 V_{RHE}, 1 V s^{-1}, 0.1 M $HClO_4$).

	diameter, before [nm]	circularity, before []	diameter, after [nm]	circularity, after []	number of events
**no changes**	3.59 ± 0.78	0.89 ± 0.02	3.58 ± 0.79	0.90 ± 0.01	160
**dissolution**	3.47 ± 0.74	0.89 ± 0.01	2.92 ± 0.84	0.90 ± 0.01	150
**detachment**	2.59 ± 0.74	0.91 ± 0.01			92
**deposition**	3.20 ± 0.62	0.90 ± 0.01	3.64 ± 0.63	0.89 ± 0.02	64
**attachment**			2.90 ± 1.02	0.90 ± 0.02	28
**agglomeration**	3.62 ± 0.96	0.90 ± 0.02	3.99 ± 0.68	0.88 ± 0.01	15
**complete Ostwald ripening**	4.50 ± 0.95	0.87 ± 0.01	4.34 ± 1.00	0.89 ± 0.01	4
**Ostwald ripening**	3.25 ± 0.42	0.91 ± 0.01	3.45 ± 0.46	0.90 ± 0.01	3

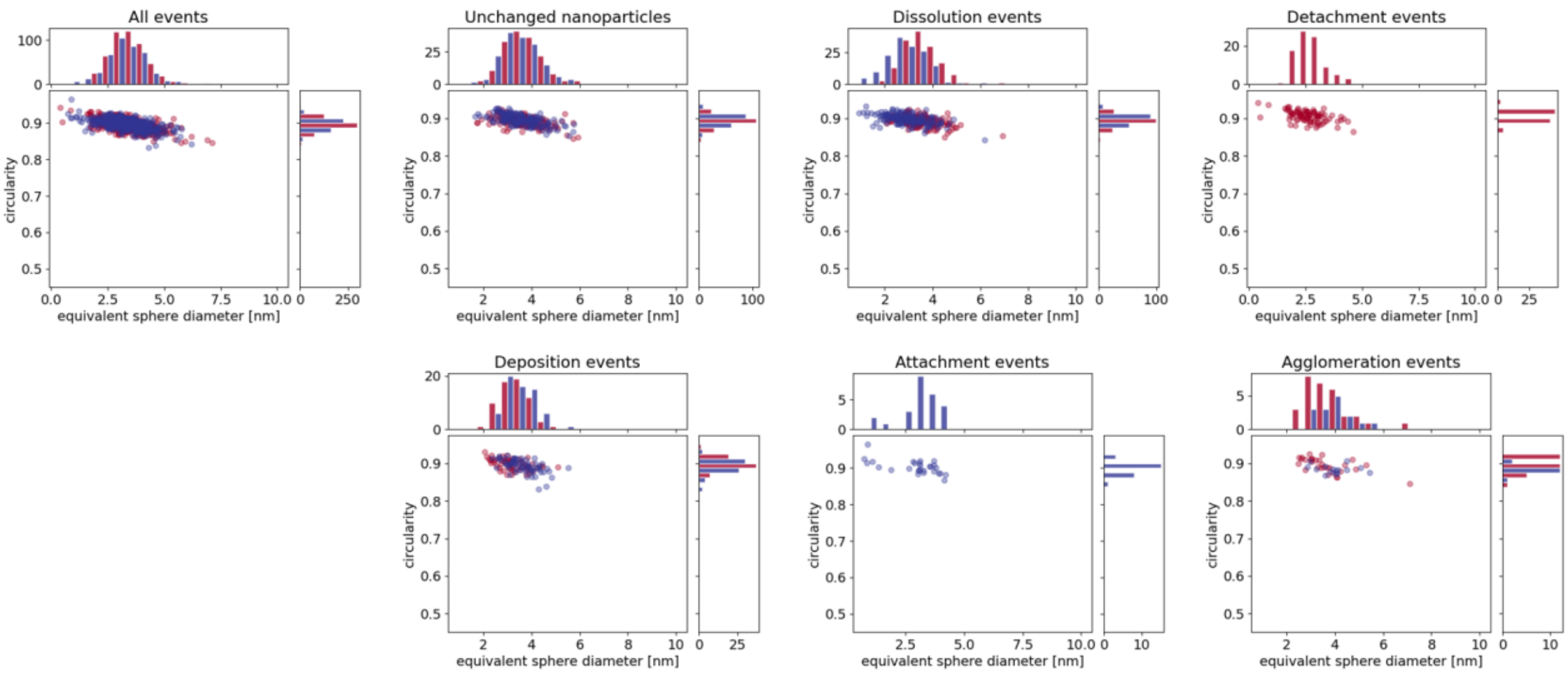

Supplementary Fig. 14. Degradation events for Pt-Co nanoparticles during potential cycling activation, followed by degradation (automated detection, automated association). Scatter plots with histograms showing circularities and equivalent sphere diameters before (red) and after (blue) activation (200 cycles, 0.05–1.2 V_{RHE}, 300 mV s^{-1}, 0.1 M $HClO_4$) and degradation (5000 cycles, 0.4–1.2 V_{RHE}, 1 V s^{-1}, 0.1 M $HClO_4$) for all events and individual degradation mechanisms. Source data are provided as a Source Data file.

SI 8. Modeling metal dissolution and carbon corrosion on model nanoparticles

Supplementary Note 3: Details on theoretical modeling of particle evolution during activation

The mathematical model of metal dissolution and carbon corrosion describes morphological development of the catalyst as a consequence of electrochemical reactions taking place on the surface of catalyst particles and the adjacent carbon surface. The model aims to describe the changes in sizes (r_i) and positions ($\mathbf{x}_i$) of a limited set of particles, observed with STEM.

a) Particle size

The change of particle sizes was modelled by describing the electrochemical processes of catalyst dissolution and redeposition. To simplify the modelling, it was assumed that the catalyst particle surface is always formed of Pt, a reasonable first approximation given that the small analyzed nanoparticles contain approximately 65 at.% of Pt.[3] This allows for the use of well-established electrochemical processes on the Pt surface. Pt dissolution and redeposition lead to a direct change in particle size.

PtDD: $\mathrm{Pt} \rightleftharpoons \mathrm{Pt}^{2+} + 2\mathrm{e}^-;$ $\quad E_{\mathrm{DD}} = 1.155\ \mathrm{V_{RHE}}$ (1)

Crucially, the equilibrium electric potential of a catalyst particle is affected by its size via the Kelvin term: $E_{\mathrm{DD}}(r) = E_{\mathrm{DD}} - \frac{1}{2F}\frac{\sigma_{\mathrm{Pt}} M_{\mathrm{Pt}}}{\rho_{\mathrm{Pt}} r}$, where *r* is the particle radius, σ_{Pt} surface tension, M_{Pt} molar mass, ρ_{Pt} density, and F Faraday constant.

The Kelvin term lowers the equilibrium potential of smaller particles more than larger particles, leading to faster dissolution and potential transfer of Pt from smaller towards larger particles.[7] Dissolution and redeposition of Pt were modelled as a Butler-Volmer reaction, describing the time derivative of particles:[8]

$$\frac{dr_i}{dt} = -\frac{M_{\mathrm{Pt}}}{\rho_{\mathrm{Pt}}}\left(k_{\mathrm{Dis}} e^{\frac{2\alpha_{\mathrm{DD}} F\left(U(t)-E_{\mathrm{DD}}(r_i)\right)}{RT}} - k_{\mathrm{Dep}} \frac{c_{\mathrm{Pt}^{2+},i}}{c_{\mathrm{Pt}^{2+},\mathrm{ref}}} e^{\frac{-2(1-\alpha_{\mathrm{DD}}) F\left(U(t)-E_{\mathrm{DD}}(r_i)\right)}{RT}}\right), \quad (2)$$

where k_{Dis} and k_{Dep} are reaction rate constants of dissolution and deposition, α_{DD} is the charge transfer coefficient, and R is the gas constant. The dynamics is primarily driven by the changes in electric potential $U(t)$, but is also significantly affected by temperature T and the concentrations of Pt^{2+} ions in the vicinity of the i-th particle $c_{\mathrm{Pt}^{2+},i}$.

To describe the dynamics of these concentrations, we assigned each particle a control volume V_i, defined by a Voronoi diagram, attributed to the particle positions $\mathbf{x}_i$.[9] The change of Pt^{2+} ions concentration due to dissolution and redeposition is thus calculated as

$$\left(\frac{dc_{\mathrm{Pt}^{2+},i}}{dt}\right)_{\mathrm{DD}} = \frac{1}{V_i}\frac{\rho_{\mathrm{Pt}}}{M_{\mathrm{Pt}}}\frac{dr_i}{dt}. \tag{3}$$

The transfer of Pt^{2+} ions between neighboring Voronoi volumes is controlled by diffusion

$$\left(\frac{dc_{\mathrm{Pt}^{2+},i}}{dt}\right)_{\mathrm{dif}} = \frac{1}{V_i}\sum_{j,\mathrm{ngh}}\frac{D_{\mathrm{Pt}^{2+}}S_{ij}}{d_{ij}}\left(c_{\mathrm{Pt}^{2+},j} - c_{\mathrm{Pt}^{2+},i}\right) \tag{4}$$

where $D_{\mathrm{Pt}^{2+}}$ is Pt^{2+} ion diffusivity, S_{ij} the contact surface between i-th and j-th Voronoi volumes, and d_{ij} the distance between i-th and j-th particle, with summation taken over neighbouring Voronoi volumes. The loss of Pt due to diffusion into the electrolyte is described similarly:

$$\left(\frac{dc_{\mathrm{Pt}^{2+},i}}{dt}\right)_{\mathrm{loss}} = \frac{1}{V_i}\sum_{j,\mathrm{ext}}\frac{D_{\mathrm{Pt}^{2+}}S_{ij}}{d_{ij}}\left(c_{\mathrm{Pt}^{2+},\mathrm{el}} - c_{\mathrm{Pt}^{2+},i}\right) \tag{5}$$

with S_{ij} and d_{ij} describing the contact surface and distance to the exterior of the Voronoi diagram.

b) Detachment and agglomeration

Detachment and agglomeration of catalyst particles are described in the model as a result of corrosion of carbon support around the particles, which is in turn caused by oxidation processes on Pt and the carbon surface.[10] The corrosion is initiated by the formation of hydroxide and oxide groups at different equilibrium potentials:

$$\begin{aligned}
&\text{C1:}\quad \mathrm{C^* + H_2O \rightleftharpoons C-OH + H^+ + e^-}; && E_{\mathrm{C1}} = 0.29\ \mathrm{V_{RHE}}\\
&\text{C2:}\quad \mathrm{C-OH \rightleftharpoons C=O + H^+ + e^-}; && E_{\mathrm{C2}} = 0.80\ \mathrm{V_{RHE}}\\
&\text{Pt1:}\quad \mathrm{Pt^* + H_2O \rightleftharpoons Pt-OH + H^+ + e^-}, && E_{\mathrm{Pt1}} = 0.79\ \mathrm{V_{RHE}}\\
&\text{Pt2:}\quad \mathrm{Pt-OH \rightleftharpoons Pt=O + H^+ + e^-}; && E_{\mathrm{Pt2}} = 0.80\ \mathrm{V_{RHE}}
\end{aligned} \tag{6}$$

The dynamics of these processes on the i-th particle are described by Butler-Volmer equations.[8]

$$\begin{aligned}
r_{\mathrm{C1},i} &= k_{\mathrm{C1}}\left(a_{\mathrm{H_2O}}\Theta_{\mathrm{C},i}e^{\alpha_{\mathrm{C1}}F(U(t)-E_{\mathrm{C1}})/RT} - a_{\mathrm{H}^+}\Theta_{\mathrm{COH},i}e^{-(1-\alpha_{\mathrm{C1}})F(U(t)-E_{\mathrm{C1}})/RT}\right),\\
r_{\mathrm{C2},i} &= k_{\mathrm{C2}}\left(\Theta_{\mathrm{COH},i}e^{\alpha_{\mathrm{C2}}F(U(t)-E_{\mathrm{C2}})/RT} - a_{\mathrm{H}^+}\Theta_{\mathrm{CO},i}e^{-(1-\alpha_{\mathrm{C2}})F(U(t)-E_{\mathrm{C2}})/RT}\right),\\
r_{\mathrm{Pt1},i} &= k_{\mathrm{Pt1}}\left(a_{\mathrm{H_2O}}\Theta_{Pt}e^{\alpha_{\mathrm{Pt1}}F(U(t)-E_{\mathrm{Pt1}}(r_i)-r_{\mathrm{Ox}}\Theta_{\mathrm{Ox}})/RT} - k_{\mathrm{rev}}a_{\mathrm{H}^+}\Theta_{\mathrm{PtOH},i}e^{-(1-\alpha_{\mathrm{Pt1}})F(U(t)-E_{\mathrm{Pt1}}(r_i))/RT}\right),\\
r_{\mathrm{Pt2},i} &= k_{\mathrm{Pt2}}\left(\Theta_{\mathrm{PtOH},i}e^{\alpha_{\mathrm{Pt2}}F(U(t)-E_{\mathrm{Pt2}}(r_i)-r_{\mathrm{Ox}}\Theta_{\mathrm{Ox}})/RT} - k_{\mathrm{rev}}a_{\mathrm{H}^+}\Theta_{\mathrm{PtO},i}e^{-(1-\alpha_{\mathrm{Pt2}})F(U(t)-E_{\mathrm{Pt2}}(r_i))/RT}\right).
\end{aligned} \tag{7}$$

Reaction rates are determined by reaction rate constants k_j, equilibrium potentials E_j, and charge transfer coefficients α_j for each of the reactions (j = C1, C2, Pt1, Pt2), as well as by relative surface coverages of i-th particle with PtOH groups ($\Theta_{\mathrm{PtOH},i}$), PtO groups ($\Theta_{\mathrm{PtO},i}$), and unoxidised surface Pt* ($\Theta_{\mathrm{Pt},i}$), and relative coverage of its surrounding carbon support with COH groups ($\Theta_{\mathrm{COH},i}$), CO groups ($\Theta_{\mathrm{CO},i}$), and unoxidised surface C* ($\Theta_{\mathrm{C},i}$). $a_{\mathrm{H_2O}}$ and a_{H^+} denote water and proton activity and r_{Ox} describes the interaction between Pt surface oxides, with $\Theta_{\mathrm{Ox}} = \Theta_{\mathrm{PtOH}} +$

Θ_{PtO}. As for the case of Pt dissolution, the equilibrium potential of Pt oxidation is affected by particle size via the Kelvin term

$$E_{\mathrm{Pt1}}(r) = E_{\mathrm{Pt1}} + \frac{1}{2Fr}\left(\frac{\sigma_{\mathrm{PtOH}}M_{\mathrm{PtOH}}}{\rho_{\mathrm{PtOH}}} - \frac{\sigma_{\mathrm{Pt}}M_{\mathrm{Pt}}}{\rho_{\mathrm{Pt}}}\right), \quad (8)$$

$$E_{\mathrm{Pt2}}(r) = E_{\mathrm{Pt2}} + \frac{1}{2Fr}\left(\frac{\sigma_{\mathrm{PtO}}M_{\mathrm{PtO}}}{\rho_{\mathrm{PtO}}} - \frac{\sigma_{\mathrm{PtOH}}M_{\mathrm{PtOH}}}{\rho_{\mathrm{PtOH}}}\right).$$

with σ, M and ρ referring to surface tension, molar mass, and density of the species.

Surface coverages of oxide groups $\Theta_{\mathrm{COH},i}$, $\Theta_{\mathrm{CO},i}$, $\Theta_{\mathrm{PtOH},i}$ and $\Theta_{\mathrm{PtO},i}$ determine the rates of carbon corrosion via two electrochemical reactions. Carbon hydroxide groups COH can react either with water or with Pt hydroxide PtOH, via reactions

$$\text{C3:}\quad \mathrm{C-OH + H_2O \rightarrow C^* + CO_2 + 3H^+ + 3e^-}; \qquad E_{\mathrm{C2}} = 0.96\ \mathrm{V_{RHE}}$$

$$\text{PtC:}\ \mathrm{Pt-OH + C-OH \rightarrow C^* + Pt^* + CO_2 + 2H^+ + 2e^-}; \qquad E_{\mathrm{PtC}} = 0.62\ \mathrm{V_{RHE}} \quad (9)$$

with reaction rates

$$r_{\mathrm{PtC},i} = k_{\mathrm{PtC}}\Theta_{\mathrm{COH},i}\Theta_{\mathrm{PtOH},i}e^{2\alpha_{\mathrm{PtC}}F(U(t)-E_{\mathrm{PtC}})/RT}, \quad (10)$$

$$r_{\mathrm{C3},i} = k_{\mathrm{C3}}a_{\mathrm{H_2O}}\Theta_{\mathrm{COH},i}e^{3\alpha_{\mathrm{C3}}F(U(t)-E_{\mathrm{C3}})/RT}.$$

The dynamics of oxide surface growth is determined by reaction rates $r_{\mathrm{Pt1},i}$, $r_{\mathrm{Pt2},i}$, $r_{\mathrm{C1},i}$, $r_{\mathrm{C2},i}$, and surface density of defect sites Γ_{Pt} and Γ_{C} on Pt and carbon surface, respectively.[8,11]

$$\frac{d\Theta_{\mathrm{PtOH},i}}{dt} = \frac{F}{\Gamma_{\mathrm{Pt}}}\left[r_{\mathrm{Pt1},i} - r_{\mathrm{Pt2},i} - \frac{d}{r_i}r_{\mathrm{PtC},i}\right],$$

$$\frac{d\Theta_{\mathrm{PtO}}}{dt} = \frac{F}{\Gamma_{\mathrm{Pt}}}r_{\mathrm{Pt2},i}, \quad (11)$$

$$\frac{d\Theta_{\mathrm{COH},i}}{dt} = \frac{F}{\Gamma_{\mathrm{C}}}\left[r_{\mathrm{C1},i} - r_{\mathrm{C2},i} - r_{\mathrm{C3},i} - r_{\mathrm{PtC},i}\right],$$

$$\frac{d\Theta_{\mathrm{CO},i}}{dt} = \frac{F}{\Gamma_{\mathrm{C}}}r_{\mathrm{C2},i}.$$

Furthermore, carbon corrosion rates $r_{\mathrm{PtC},i}$ and $r_{\mathrm{C3},i}$ determine how the contact surface between the catalyst particle and carbon support changes over time. In the model, the contact surface of the i-th particle was described by the contact angle $\varphi_{\mathrm{C},i}$ resulting in the contact surface

$$S_{\mathrm{C},i} = 2\pi r_i^2(1 - \cos\varphi_{\mathrm{C},i}). \quad (12)$$

Assuming that carbon corrosion takes place up to the distance d from the particle, the rate of change of contact angle can be written as

$$\frac{d\varphi_{\mathrm{C},i}}{dt} = -\frac{3(d + 2r_i)}{2(d^2 + 3\,d\,r_i + 3\,r_i^2)}\frac{\rho_{\mathrm{C}}}{M_{\mathrm{C}}}\left(r_{\mathrm{PtC},i} + r_{\mathrm{C3},i}\right) \quad (13)$$

where ρ_C and M_C are the density and molar mass of carbon.

Carbon corrosion can lead to the detachment and agglomeration of particles even before the contact angle $S_{C,i}$ reduces to zero, provided that the attractive forces between particles are sufficiently strong. The agglomeration process was modelled by comparing the attraction forces between particles with the adhesion force between particles and the carbon support.

It was assumed in the model that the i-th and j-th particles are attracted by the Casimir force[12]

$$F_{\mathrm{cas},ij} = \frac{\alpha_{\mathrm{cas}}\pi^3 r_i r_j}{720 L_{ij}^3 (r_i + r_j)}\left(1 - \frac{L_{ij}}{2(r_i + r_j)} + \frac{L_{ij}}{6}\left(\frac{1}{r_i} + \frac{1}{r_j}\right)\right) \quad (14)$$

which depends on the particle radii r_i and r_j, and the distance between particle surfaces L_{ij}.

The total attraction force on the i-th particle is therefore

$$\mathbf{F}_{\mathrm{attr},i} = \sum_j F_{\mathrm{cas},ij} \frac{\mathbf{r}_j - \mathbf{r}_i}{|\mathbf{r}_j - \mathbf{r}_i|}. \quad (15)$$

The adhesion force $\mathbf{F}_{\mathrm{adh},i}$ on the particle is exactly opposite the attraction force, as long as attraction is not too strong. The limiting force $F_{\mathrm{adh,max},i}$that can be produced by adhesion is assumed to be directly proportional to the contact surface, $F_{\mathrm{adh,max},i} = \alpha_{\mathrm{adh}} S_{C,i}$. The total force on the i-th particle is therefore

$$\mathbf{F}_i = \mathbf{F}_{\mathrm{attr},i}\left(1 - \frac{\min(|\mathbf{F}_{\mathrm{attr},i}|, F_{\mathrm{adh},L,i})}{|\mathbf{F}_{\mathrm{attr},i}|}\right). \quad (16)$$

The movement of particles due to force $\mathbf{F}_i$ was modelled under the assumption of strong drag, resulting in velocity being proportional to the force, changing the particle position $\mathbf{x}_i$ as

$$\frac{d\mathbf{x}_i}{dt} = \mathbf{v}_i = \beta \mathbf{F}_i. \quad (17)$$

In total, 10 variables are modelled for each particle: particle size r_i, 3 coordinates of position $\mathbf{x}_i$, Pt^{2+} ion concentration $c_{\mathrm{Pt}^{2+},i}$, oxide coverages $\Theta_{\mathrm{PtOH},i}$, $\Theta_{\mathrm{PtO},i}$, $\Theta_{\mathrm{COH},i}$, $\Theta_{\mathrm{CO},i}$ and contact angle $\varphi_{C,i}$.

Calibration of the model, aimed at achieving the best fit between experimental data and model results, was performed by adjusting the values of the following model parameters: reaction rate constants k_{C1}, $k_{C2}, k_{C3}, k_{\mathrm{Pt1}}, k_{\mathrm{Pt2}}, k_{\mathrm{PtC}}, k_{\mathrm{PtDD}}$, Pt^{2+} ion diffusivity $D_{\mathrm{Pt}^{2+}}$, Pt^{2+} ion concentration in the electrolyte $c_{\mathrm{Pt}^{2+},\mathrm{el}}$, and relative strenghts of Casimir (α_{cas}) and adhesion force (α_{adh}).

The values of other parameters were adopted from the literature. The values of all model parameters are listed in Supplementary Table 11.

Supplementary Table 11. List of parameters used in the model. The values of parameters are accompanied by a short description and the source.

parameter	value	units	description	source
E_{C1}	0.2	V	Equilibrium potential for reaction C1	Ref. [13]
E_{Pt1}	0.7	V	Equilibrium potential for reaction Pt1	Ref. [13]
E_{C2}	0.8	V	Equilibrium potential for reaction C2	Ref. [13]
E_{Pt2}	0.8	V	Equilibrium potential for reaction Pt2	Ref. [13]
E_{C3}	0.95	V	Equilibrium potential for reaction C3	Ref. [13]
E_{PtC}	0.65	V	Equilibrium potential for reaction PtC	Ref. [13]
E_{PtDis}	1.188	V	Equilibrium potential for Pt dissolution	Ref. [14]
α_{C1}	0.5	–	Charge transfer coefficient for reaction C1	assumed
α_{Pt1}	0.5	–	Charge transfer coefficient for reaction Pt1	assumed
α_{C2}	0.5	–	Charge transfer coefficient for reaction C2	assumed
α_{Pt2}	0.5	–	Charge transfer coefficient for reaction Pt2	assumed
α_{C3}	0.5	–	Charge transfer coefficient for reaction C3	assumed
α_{PtC}	0.5	–	Charge transfer coefficient for reaction PtC	assumed
α_{PtDis}	0.5	–	Charge transfer coefficient for Pt dissolution	assumed
k_{C1}	3.01×10^{-9}	$\mathrm{mol\ m^{-2} s^{-1}}$	Reaction rate constant for reaction C1	fitted
k_{Pt1}	4.77×10^{-5}	$\mathrm{mol\ m^{-2} s^{-1}}$	Reaction rate constant for reaction Pt1	fitted
k_{C2}	8.79×10^{-7}	$\mathrm{mol\ m^{-2} s^{-1}}$	Reaction rate constant for reaction C2	fitted
k_{Pt2}	1.45×10^{-5}	$\mathrm{mol\ m^{-2} s^{-1}}$	Reaction rate constant for reaction Pt2	fitted
k_{C3}	1.43×10^{-13}	$\mathrm{mol\ m^{-2} s^{-1}}$	Reaction rate constant for reaction C3	fitted
k_{PtC}	2.19×10^{-11}	$\mathrm{mol\ m^{-2} s^{-1}}$	Reaction rate constant for reaction PtC	fitted
k_{Dis}	5.92×10^{-9}	$\mathrm{mol\ m^{-2} s^{-1}}$	Reaction rate constant for Pt dissolution	fitted
k_{Dep}	2.08×10^{-9}	$\mathrm{mol\ m^{-2} s^{-1}}$	Reaction rate constant for Pt deposition	fitted
Γ_{Pt}	2.15	$\mathrm{As\ m^{-2}}$	Pt oxidation surface area	Ref. [10]
Γ_{C}	4.6	$\mathrm{As\ m^{-2}}$	Carbon oxidation surface area	Ref. [10]
d	1	nm	Carbon corrosion depth	Ref. [10]
$D_{\mathrm{Pt^{2+}}}$	2.11×10^{-14}	$\mathrm{m^2 s^{-1}}$	Diffusivity of $\mathrm{Pt^{2+}}$	fitted
$c_{\mathrm{Pt^{2+},ref}}$	1	$\mathrm{mol\ l^{-1}}$	Reference $\mathrm{Pt^{2+}}$concentration	Ref. [14]
$c_{\mathrm{Pt^{2+},el}}$	4.93×10^{-3}	$\mathrm{mol\ l^{-1}}$	$\mathrm{Pt^{2+}}$concentration in electrolyte	fitted
r_{Ox}	30	$\mathrm{kJ\ mol^{-1}}$	Pt oxide interaction	Ref. [14]
k_{rev}	0.3	–	Pt oxidation reversibility	Ref. [15]
α_{Cas}	10^{-27}	$\mathrm{Nm^2}$	Casimir force strength	assumed
α_{Adh}	10^{9}	$\mathrm{N\ m^{-2}}$	Adhesion force strength	fitted
β	0.05	$\mathrm{m\ N^{-1} s^{-1}}$	Drag coefficient	assumed
ρ_{Pt}	21090	$\mathrm{kg\ m^{-3}}$	Pt density	Ref. [14]
ρ_{PtOH}	14170	$\mathrm{kg\ m^{-3}}$	Pt-OH density	Ref. [10]
ρ_{PtO}	14100	$\mathrm{kg\ m^{-3}}$	Pt=O density	Ref. [14]
ρ_{C}	2000	$\mathrm{kg\ m^{-3}}$	Carbon density	-
σ_{Pt}	2.73	$\mathrm{J\ m^{-2}}$	Pt surface tension	Ref. [14]
σ_{PtOH}	1.34	$\mathrm{J\ m^{-2}}$	Pt-OH surface tension	Ref. [10]
σ_{PtO}	1	$\mathrm{J\ m^{-2}}$	Pt=O surface tension	Ref. [14]
M_{Pt}	195	$\mathrm{g\ mol^{-1}}$	Pt molar mass	Ref. [14]
M_{PtOH}	212	$\mathrm{g\ mol^{-1}}$	Pt-OH molar mass	Ref. [10]
M_{PtO}	211	$\mathrm{g\ mol^{-1}}$	Pt=O molar mass	Ref. [14]
M_{C}	12	$\mathrm{g\ mol^{-1}}$	Carbon molar mass	-
$M_{\mathrm{H_2O}}$	18	$\mathrm{g\ mol^{-1}}$	Water molar mass	-

The geometric parameters of the 11 model nanoparticles before and after simulated potential cycling conditions are listed in Supplementary Table 12.

Supplementary Table 12. Radii, contact angles, and contact surfaces for all analyzed model nanoparticles.

particle id	radius, before [nm]	angle, before [rad]	surface, before [nm^2]	radius, after [nm]	angle, after [rad]	surface, after [nm^2]	surface change [nm^2]
**1**	1.99	1.57	24.83	1.86	0.08	0.08	24.75
**2**	1.40	1.57	12.29	0.99	0.01	0.00	12.29
**3**	1.61	1.57	16.21	1.38	0.01	0.00	16.21
**4**	1.99	1.57	24.90	1.86	0.08	0.08	24.82
**5**	1.85	1.57	21.49	1.70	0.03	0.01	21.48
**6**	1.85	1.57	21.55	1.70	0.03	0.01	21.55
**7**	2.77	1.57	48.11	2.70	0.39	3.38	44.72
**8**	2.19	1.57	30.25	2.09	0.18	0.43	29.81
**9**	1.82	1.57	20.87	1.67	0.03	0.01	20.86
**10**	1.21	1.57	9.25	0.00	0.13	0.00	9.25
**11**	1.77	1.57	19.73	1.61	0.02	0.00	19.73

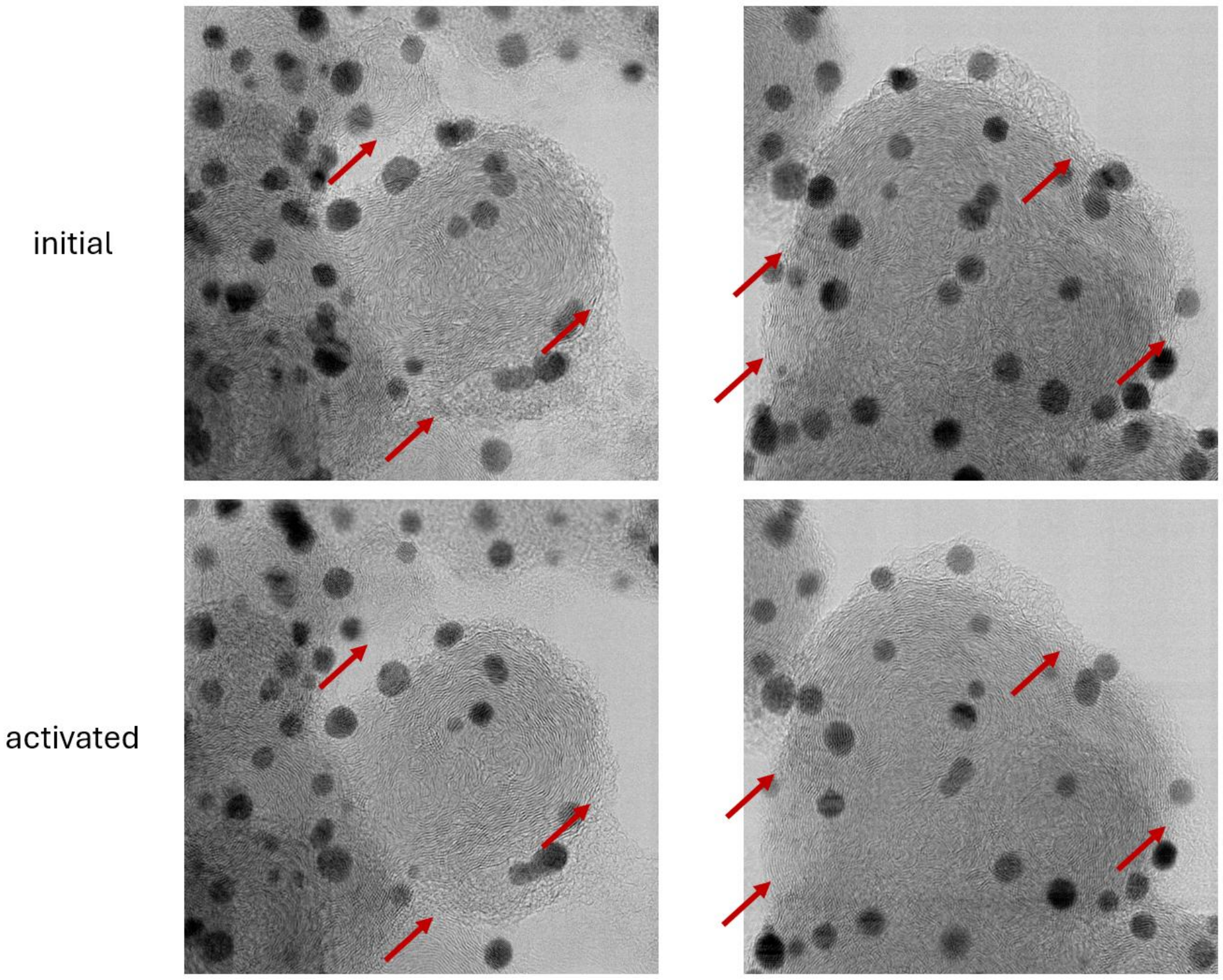

Supplementary Fig. 15. Instances of carbon corrosion in IL-BF-STEM images. Images of the Pt-Co/C electrocatalyst before (top) and after (bottom) activation (200 cycles, 0.05–1.2 V_{RHE}, 300 mV s^{-1}, 4 M $HClO_4$) with red arrows pointing to carbon support changes.

Supplementary References

1. Wouters, C., Kestens, V., Verleysen, E. & Mast, J. Assessing particle count in electron microscopy measurements of nanomaterials to support regulatory guidance. *Sci. Rep.* **15**, 11803 (2025).
2. Yu, H. *et al.* Tracking Nanoparticle Degradation across Fuel Cell Electrodes by Automated Analytical Electron Microscopy. *ACS Nano* **16**, 12083–12094 (2022).
3. Moriau, L. J. *et al.* Resolving the nanoparticles' structure-property relationships at the atomic level: a study of Pt-based electrocatalysts. *iScience* **24**, 102102 (2021).
4. Tabernik, D., Muhovič, J. & Skočaj, D. Dense center-direction regression for object counting and localization with point supervision. *Pattern Recognit.* **153**, 110540 (2024).
5. Tabernik, D., Muhovič, J., Urbas, M. & Skočaj, D. Center Direction Network for Grasping Point Localization on Cloths. *IEEE Robot. Autom. Lett.* **9**, 8913–8920 (2024).
6. Kendall, A., Gal, Y. & Cipolla, R. Multi-Task Learning Using Uncertainty to Weigh Losses for Scene Geometry and Semantics. in *Proceedings of the IEEE Conference on Computer Vision and Pattern Recognition (CVPR)* 7482–7491 (2018).
7. Plieth, W. J. Electrochemical Properties of Small Clusters of Metal Atoms and Their Role in Surface Enhanced Raman Scattering. *J. Phys. Chem.* **86**, 3166–3170 (1982).
8. Kregar, A., Gatalo, M., Maselj, N., Hodnik, N. & Katrašnik, T. Temperature dependent model of carbon supported platinum fuel cell catalyst degradation. *J. Power Sources* **514**, 230542 (2021).
9. Voronoi, G. Nouvelles applications des parametres continus a la theorie des formes quadratiques. Sur quelques proprietes des formes quadratiques positives parfaites. *Journal für die reine und angewandte Mathematik* **133**, 97–102 (1908).
10. Kregar, A., Tavčar, G., Kravos, A. & Katrašnik, T. Predictive system-level modeling framework for transient operation and cathode platinum degradation of high temperature proton exchange membrane fuel cells. *Appl. Energy* **263**, 114547 (2020).
11. Kregar, A., Kravos, A. & Katrašnik, T. Methodology for Evaluation of Contributions of Ostwald Ripening and Particle Agglomeration to Growth of Catalyst Particles in PEM Fuel Cells. *Fuel Cells* **20**, 487–498 (2020).
12. Teo, L. P. Casimir effect between two spheres at small separations. *Physical Review D - Particles, Fields, Gravitation and Cosmology* **85**, 045027 (2012).
13. Pandy, A. *et al.* A Carbon Corrosion Model to Evaluate the Effect of Steady State and Transient Operation of a Polymer Electrolyte Membrane Fuel Cell. *J. Electrochem. Soc.* **160**, F972–F979 (2013).
14. Bi, W., Gray, G. E. & Fuller, T. F. PEM fuel cell PtC dissolution and deposition in nafion electrolyte. *Electrochemical and Solid-State Letters* **10**, 101–104 (2007).
15. Darling, R. M. & Meyers, J. P. Kinetic Model of Platinum Dissolution in PEMFCs. *J. Electrochem. Soc.* **150**, A1523 (2003).
