# Supplementary material for: Large-scale identical-location electron microscopy enables quantifying nanoparticulate electrocatalyst degradation
Source: Nat Commun. 2026 Oct 10;17:11512. doi: 10.1038/s41467-026-78329-8 (PMC13745521; doi:10.1038/s41467-026-78329-8)
Supplement: Supplementary file 2 [file 41467_2026_78329_MOESM2_ESM.pdf]

nature portfolio

Peer Review File

Large-scale identical-location electron microscopy enables quantifying nanoparticulate electrocatalyst degradation

Corresponding Author: Dr Ana Rebeka Kamšek

This manuscript has been previously reviewed at another journal. This document only contains information relating to versions considered at Nature Communications.

This file contains all reviewer reports in order by version, followed by all author rebuttals in order by version.

Version 0:

Reviewer comments:

Reviewer #1

(Remarks to the Author)
The article "Large-scale identical-location electron microscopy enables quantifying nanoparticulate electrocatalyst degradation" attempts to present the case that by increasing the number of particles analyzed over previous studies opens the door to new insights into nanoparticle degradation mechanisms. The authors analyzed nearly 1000 particles in their study, and demonstrate a three-step event classification workflow for categorizing changes in particle morphology into different types of degradation mechanisms. They then go on to demonstrate how this image analysis workflow can be automated in order to be applied to even larger particle populations for which manual analysis would prove impractical. The article concludes with a brief modeling effort applied to a very small subset of the data.
The terms "large-scale" and "statistically meaningful" are used regularly throughout the paper without proper definition. How would the authors define a large-scale data set in terms of numbers of nanoparticles? What is the metric for defining when the data set becomes statistically meaningful?
The authors emphasize the ability of IL-STEM to image the same nanoparticles before and after electrochemical treatment, but never address the differences in environment between the modified floating electrode (MFE) used in this study to the actual fuel cell environment in an operating cell and how that might impact observed degradation mechanisms. Could the authors provide a comparison of these MFE-derived results to changes in median particle size and composition observed in a single cell test performed under the same degradation protocol? Indeed, the fact that the catalysts degrade more under the activation protocol than the deactivation protocol suggest the insights from this study may not be transferable to actual fuel cell operation in membrane electrode assemblies. This is particularly concerning as the authors chose a wider potential window than typically used in fuel cell accelerated stress tests to induce increased degradation but still saw very limited changes in the average particle size.
Ostwald ripening is typically identified as a primary degradation mechanism in modern MEA tests. This study revealed very limited Ostwald ripening events. Is this a discrepancy of the MFE vs MEA environment, or can the authors provide another explanation? Could the authors also explain what is meant by complete Ostwald ripening in Supp Table 3?
Can the authors explain why the modeling at the end of the paper was not applied to the full dataset? It seems an opportunity is missed here to take advantage of the increased sampling which is the key achievement of this study.
Can the authors clarify if the neural network-based tools for automated analysis were used for the analysis presented in Figure 3 and 4? It appears these tools were developed after the fact, and the comparison between the automated and manually measured datasets feels like it would be better suited for a separate article.

(Remarks on code availability)

Reviewer #2

(Remarks to the Author)
The authors report on an image analysis protocol used to track morphological changes in nanoparticle catalysts. The main issue with this study is the novelty of the results presented, relative to those reported in previous studies on similar catalyst

degradation. It would be great if the authors could clarify this in their manuscript. Additionally, a few other points are recommended for further consideration.
• It is unclear what is meant by a "theoretical model confirming that detached particles can dissolve completely". If detached particles no longer maintain an electrical connection, further dissolution would not be expected. Please elaborate.
• It is also unclear how the authors validate the distinction between detached and fully dissolved particles in experiments where the location is identical. The same applies to redeposited and reattached particles. It seems more appropriate to classify particles as either dissolved or detached than to separate these processes into two distinct mechanisms.
• The authors report that EDX measurements of different regions yield varying results in terms of the shift in the Pt/Co ratio, and attribute these effects to the sample's inherent heterogeneity. EDX maps of the same regions would also be helpful in order to better assess whether there is any correlation between the location of the particles on the carbon support and the observed changes in composition, and to evaluate whether pure Co contributes to the averaged results.
• Additionally, reference spectra of pure Pt and Co should be included in the XRD plot, and the diffraction peaks corresponding to pure Co should be labelled.
• Finally, the authors attribute the lower current values observed in the cyclic voltammograms to a decrease in active sites. However, the cyclic voltammetry measurements in the supplementary information are presented as absolute current values. While a lower absolute current could be consistent with fewer active sites, this alone is insufficient to support such a conclusion. To validate this claim, the data should be normalised to current density.

(Remarks on code availability)

Reviewer #3

(Remarks to the Author)
Recommendation: Minor Revision

This manuscript presents a robust and scalable methodology that combines identical-location STEM (IL-STEM) with machine-learning-assisted image analysis to quantify electrocatalyst degradation at the single-particle level. In particular, the work effectively addresses longstanding limitations of conventional TEM studies, namely limited statistical sampling and challenges in quantitative analysis. By systematically tracking and analyzing hundreds of nanoparticles, the authors move beyond the small, often anecdotal datasets that have traditionally constrained this field, enabling statistically meaningful insights into degradation mechanisms.
Importantly, as automated electron microscopy and high-throughput imaging continue to advance, approaches such as this are expected to play an increasingly important role in accelerating the discovery and optimization of durable catalyst materials. While the conceptual advance is, in my view, primarily methodological and somewhat incremental relative to prior IL-STEM studies, the study is well executed and establishes a versatile framework that should find broad application across a wide range of catalyst systems and degradation studies. I therefore recommend publication following minor revision.
More specific comments are:
1. Section c ("Theoretical modeling of Pt-Co particle evolution during activation") provides a useful framework for understanding the relationship between carbon support evolution and particle detachment. It would be helpful if the authors could briefly discuss whether carbon support corrosion can be quantified in relation to the various degradation pathways identified in this work, such as Ostwald ripening, particle detachment, dissolution, and particle coalescence. Such discussion would further highlight the interconnected nature of these degradation processes and broaden the mechanistic implications of the study.

2. The activation and accelerated degradation protocols employ substantially different potential windows, which may give rise to distinct degradation pathways and kinetics. It would be helpful for the authors to further discuss how the applied voltage window may influence the prevalence of the observed degradation mechanisms. Such discussion would help clarify the extent to which the reported trends may depend on the electrochemical protocol and would further broaden the applicability of the conclusions.

(Remarks on code availability)

Version 1:

Reviewer comments:

Reviewer #1

(Remarks to the Author)
The addition of the new text "we consider a randomly selected dataset of several hundred nanoparticles to be statistically meaningful" is an opinion and does not appropriately address the comment to present a quantitative metric upon which the claim "statistically meaningful" can be substantiated. The authors should present a statistical analysis showing the increase in the data set converges to a desired metric (such as a predefined standard deviation or relative margin of error). Possible examples from the literature include C. Wouters et al. Sci Rep. 2025 Apr 7;15:11803, H. Yu et al. in ACS Nano 2022, 16, 8, 12083–12094, or K.M. Aviles ACS Nanosci. Au 2025, 5, 3, 117–127

(Remarks on code availability)

Reviewer #2

(Remarks to the Author)
The authors' responses and amendments are appreciated. Thank you.

(Remarks on code availability)

Reviewer #3

(Remarks to the Author)
All raised concerns are addressed. I support a publication of this manuscript.

(Remarks on code availability)

Version 2:

Reviewer comments:

Reviewer #1

(Remarks to the Author)
The authors' have performed a convergence analysis to substantiate their claim to statistical significance, effectively addressing the prior reviewer comment.

(Remarks on code availability)

Open Access This Peer Review File is licensed under a Creative Commons Attribution 4.0 International License, which permits use, sharing, adaptation, distribution and reproduction in any medium or format, as long as you give appropriate credit to the original author(s) and the source, provide a link to the Creative Commons license, and indicate if changes were made.
In cases where reviewers are anonymous, credit should be given to 'Anonymous Referee' and the source.
The images or other third party material in this Peer Review File are included in the article’s Creative Commons license, unless indicated otherwise in a credit line to the material. If material is not included in the article’s Creative Commons

license and your intended use is not permitted by statutory regulation or exceeds the permitted use, you will need to obtain permission directly from the copyright holder.
To view a copy of this license, visit https://creativecommons.org/licenses/by/4.0/

Reviewer #1 (Remarks to the Author):

The article “Large-scale identical-location electron microscopy enables quantifying nanoparticulate electrocatalyst degradation” attempts to present the case that by increasing the number of particles analyzed over previous studies opens the door to new insights into nanoparticle degradation mechanisms. The authors analyzed nearly 1000 particles in their study, and demonstrate a three-step event classification workflow for categorizing changes in particle morphology into different types of degradation mechanisms. They then go on to demonstrate how this image analysis workflow can be automated in order to be applied to even larger particle populations for which manual analysis would prove impractical. The article concludes with a brief modeling effort applied to a very small subset of the data.

We thank the reviewer for taking the time to evaluate our manuscript and provide meaningful feedback on how to further improve the work, which we address here point by point.

1. The terms “large-scale” and “statistically meaningful” are used regularly throughout the paper without proper definition. How would the authors define a large-scale data set in terms of numbers of nanoparticles? What is the metric for defining when the data set becomes statistically meaningful?

We appreciate the opportunity to clarify these two terms. In high-magnification IL-STEM, which requires nanoscale resolution and precise particle tracking, analyzing hundreds of nanoparticles constitutes a significant improvement compared to previous studies that often either relied on reporting qualitative or averaged results, or featured a quantitative analysis for only a few nanoparticles (Godoy et al, Small Methods, 2025, e02201). Our dataset of several hundred nanoparticles therefore moves beyond a local investigation and justifies the term »large-scale«.

A dataset becomes statistically meaningful when it is large enough to capture population-level trends with confidence, and the results are not dominated by outliers. Following established practice in ex-situ transmission electron microscopy (ISO IS 21363), where particle size distributions derived from 300–500 nanoparticles are deemed sufficient (Grulke et al, Advanced Powder Technology, 2017, 28, 7, 1647-1659) to characterize a sample (particularly for a relatively narrow size distribution, as in our case), we consider our sample size as statistically meaningful. But more importantly, the sampling region is randomly selected. Quantitatively, the relative statistical uncertainty in the measured fraction of a given degradation event scales as approximately $1/\sqrt{N}$, yielding a margin of error of a few percent for a sample of several hundred nanoparticles, which is sufficient to distinguish population-level trends from random fluctuations.

To improve clarity, we have added the following sentence to the manuscript (Results section, after introducing the ground truth results):

"We consider a randomly selected dataset of several hundred nanoparticles to be statistically meaningful for this catalyst system, as this sample size is sufficient to capture population-level degradation trends within a particle ensemble with a relatively narrow size distribution."

2. The authors emphasize the ability of IL-STEM to image the same nanoparticles before and after electrochemical treatment, but never address the differences in environment between the modified floating electrode (MFE) used in this study to the actual fuel cell environment in an operating cell and how that might impact observed degradation mechanisms. Could the authors provide a comparison of these MFE-derived results to changes in median particle size

and composition observed in a single cell test performed under the same degradation protocol? Indeed, the fact that the catalysts degrade more under the activation protocol than the deactivation protocol suggest the insights from this study may not be transferable to actual fuel cell operation in membrane electrode assemblies. This is particularly concerning as the authors chose a wider potential window than typically used in fuel cell accelerated stress tests to induce increased degradation but still saw very limited changes in the average particle size.

We acknowledge the differences between catalyst testing in a laboratory setup such as the MFE and in an actual fuel cell environment, and recognize that different environments will have different impacts on specific degradation mechanisms.

While it is difficult to identify a study that uses the same cycling protocol (potential window, scan rate, number of cycles) in a fuel cell as we used in MFE, preventing a direct comparison, Yu et al. (ACS Appl. Mater. Interfaces 2022, 14, 20418-20429) directly compared degradation in an aqueous environment with a three-electrode cell to degradation in an MEA. They found that PtCo catalyst degradation in the three-electrode cell was limited relative to the MEA. Better agreement with MEA was achieved when they used an extended potential window and an electrolyte containing Pt ions. Under these modified conditions, the degraded particle size distribution and alloy composition were a better match. They also noted that these conditions increased the relative contribution of Ostwald ripening, which was more commonly observed in MEA testing.

Regarding the observation that our sample exhibited more changes after activation than degradation, we would like to emphasize that the activation protocol had a wider potential window and was applied to a pristine catalyst far from an equilibrium state. During the degradation protocol, the potential window was indeed wider than the one typically used in fuel cell accelerated stress tests, but the experiment was also carried out at a room temperature and in an environment that is not directly comparable to MEA. Observing more degradation during activation was, therefore, expected, and both datasets served as a model system for method development rather than a direct mimic of a fuel cell.

To discuss testing in MEA compared to other environments under the same degradation protocols, as well as to discuss the impact of the potential window, we have added the following discussion to the manuscript after presenting how the catalyst changed after MFE degradation:

"The influence of the potential window extends beyond general dissolution trends. Existing literature confirms that both primary degradation mechanisms, metal dissolution and carbon corrosion, depend strongly on the applied potential window. Higher upper potential limits, encountered for instance during start-up/shut-down conditions in fuel cells, significantly increase both carbon corrosion and platinum dissolution, the latter due to more pronounced nanoparticle surface oxidation. Conversely, a narrower potential window reduces degradation rates. The primary mechanisms also trigger secondary ones, leading to loss of electrochemically active surface area and catalytic activity over time. Nevertheless, even a narrow window will produce noticeable degradation under prolonged operation. Thus, the choice of electrochemical protocol importantly shapes the reported degradation trends.[52] Beyond the potential window, the testing environment itself matters: aqueous environments are not directly comparable to a membrane electrode assembly. One study found that extending the potential window and adding Pt ions to the electrolyte yielded particle size distributions and alloy compositions closer to those measured in a membrane electrode assembly.[54]"

3. Ostwald ripening is typically identified as a primary degradation mechanism in modern MEA tests. This study revealed very limited Ostwald ripening events. Is this a discrepancy of the MFE vs MEA environment, or can the authors provide another explanation? Could the authors also explain what is meant by complete Ostwald ripening in Supp Table 3?

The reviewer raises an important point about the differences between MEA and MFE environments, and experimental observations of Ostwald ripening.

Indeed, our study observed limited Ostwald ripening. The conditions during the MFE experiments were less severe than typical MEA tests, which are normally carried out at a higher temperature, with a thicker catalyst layer, and in a complex ionomer/water environment, while also resulting in different amounts of dissolved metal redeposited onto the catalyst vs. escaped out through diffusion. This alone can be expected to produce different degradation trends. In addition, the relatively open geometry of the electrochemical setup may facilitate diffusion of dissolved Pt species away from the catalyst layer, thereby reducing the probability of Pt redeposition and subsequent Ostwald ripening. This was also observed by Yu et al. (ACS Appl. Mater. Interfaces 2022, 14, 20418-20429), where additional Pt had to be dissolved in the electrolyte to simulate MEA Ostwald ripening degradation. Aside from that reason, Ostwald ripening could be limited due to a relatively narrow particle size distribution within our Pt-Co/C catalyst, resulting in a reduced driving force for this degradation phenomenon compared to a more polydisperse catalyst (Yano et al, Nano Energy 2016, 29, 323-333; Watanabe et al, J. Electroanal. Chem. 2018, 819, 359-364).

We added the following explanation of these two influences on Ostwald ripening to the first section of the Results, after mentioning previous studies on dissolution trends:

"The observed modest degree of Ostwald ripening for both cycling protocols could be attributed to both relatively mild conditions of MFE testing compared to membrane electrode assembly studies. In the MFE geometry, dissolved Pt species can diffuse away from the catalyst layer and become diluted in the larger bulk electrolyte volume, limiting local redeposition onto larger nanoparticles. This effect is likely reinforced by the narrow particle size distribution of our catalyst, which reduces the driving force for Ostwald ripening."

Lastly, we require a shrinking and a growing particle to be in relatively close spatial proximity within the same image to classify the event as Ostwald ripening, as explained in the manuscript. This means we cannot capture instances of Ostwald ripening occurring over a longer distance, or between two particles that are not present in the same imaged area, potentially leading to a smaller number of observed events.

Complete Ostwald ripening refers to a process where a smaller nanoparticle dissolves entirely, while a larger nanoparticle in the surrounding area grows. We separate these events from the rest of Ostwald ripening events, where a larger nanoparticle grows at the expense of the smaller one getting smaller, but both particles are still present in the "after" image.

We clarified this difference in the decision tree explanation:

"It does, however, distinguish between 'complete' Ostwald ripening, where the smaller particle dissolves entirely, and other Ostwald ripening events, where both particles remain but with reduced and increased sizes, respectively."

4. Can the authors explain why the modeling at the end of the paper was not applied to the full dataset? It seems an opportunity is missed here to take advantage of the increased sampling which is the key achievement of this study.

We thank the reviewer for this question. The modeling was not applied to the full dataset because its goal was mechanistic insight, not to provide another large dataset. The 11 particles were selected as representative examples from our STEM images, with initial states matching experimental data, as modeling hundreds of particles would be computationally prohibitive. A key insight, that a particle classified as "detached" may also be consistent with complete dissolution, adds interpretative value to the statistical findings without requiring a large sample.

To clarify this, we have added the following sentence to the modeling subsection:

"While applied to a limited subset of particles, the modeling is designed to provide mechanistic context for the statistical trends, not to replicate the full dataset."

5. Can the authors clarify if the neural network-based tools for automated analysis were used for the analysis presented in Figure 3 and 4? It appears these tools were developed after the fact, and the comparison between the automated and manually measured datasets feels like it would be better suited for a separate article.

We appreciate the opportunity to clarify this point. Figures 3 and 4 are based entirely on manual annotation and analysis; no neural network tools were used. These datasets constitute our ground truth. Figure 5 presents the machine-learning-assisted analysis, both examples of particle detection outputs and the corresponding results of degradation event analysis.

Regarding the reviewer's suggestion that the comparison between manual and automated analysis might be better suited for a separate article, we would like to convey that automation is central to the scalable IL-STEM approach we advocate. While we appreciate the notion that this part could, in principle, be its own paper, presenting large-scale IL-STEM without demonstrating that automated tools can achieve results sufficiently comparable to manual analysis would leave readers with an impractical proposition: more manual work, not less. Validating the automated pipeline against a manually obtained ground truth is essential to establish its credibility and to show that the methodology is ready for application to even larger datasets.

To further highlight the necessity of automation and connect the two parts of the manuscript, we have added the following sentence to the Results section, where we already discuss the limitations of a non-automated IL-STEM analysis:

“On the image analysis side, manual annotation of hundreds of nanoparticles is time-intensive and limits the practicality of this approach, motivating the development of automated analysis tools.”

Reviewer #2 (Remarks to the Author):

The authors report on an image analysis protocol used to track morphological changes in nanoparticle catalysts. The main issue with this study is the novelty of the results presented, relative to those reported in previous studies on similar catalyst degradation. It would be great if the authors could clarify this in their manuscript. Additionally, a few other points are recommended for further consideration.

We thank the reviewer for the effort they put into evaluating this manuscript and for raising the important question of novelty. To address this, we have rewritten the last part of the Abstract to clarify scalability as the paper's novelty as:

"In this work, we reveal particle size- and shape-dependent effects, showing that smaller and irregular nanoparticles are more prone to detachment, and provide a data-driven framework for probing electrocatalyst degradation at scale."

Beyond the mechanistic observations, we would also like to emphasize the methodological novelty of the work. The study establishes a scalable, progressively automated IL-STEM workflow that enables particle-resolved degradation analysis across much larger nanoparticle populations than typically addressed in identical-location studies. This framework is not limited to the present Pt-Co/C catalyst but can be adapted to other nanoparticulate electrocatalysts and electrochemical protocols, helping other researchers obtain statistically meaningful particle-level insights and identify new structure–stability relationships in future studies.

We address the remaining points in detail on the following pages.

1. It is unclear what is meant by a "theoretical model confirming that detached particles can dissolve completely". If detached particles no longer maintain an electrical connection, further dissolution would not be expected. Please elaborate.

The reviewer is correct: if a particle is physically detached from the support and no longer has electrical contact, further potential-induced electrochemical dissolution would stop.

What we intended to convey, and what the modeling shows, is that a particle that disappears from the image could be interpreted in two ways: (1) it detached and moved away, or (2) it dissolved completely while still attached.

Modelling supports the latter possibility by showing that complete dissolution is feasible for small particles under the applied potential cycles. In other words, a particle classified as "detached" based on its absence may in fact have dissolved entirely.

To avoid the confusion, we have added the following explanation (Results section, modeling subsection):

"This insight helps address an inherent ambiguity in IL-STEM: a particle that disappears from the image could have either physically detached or dissolved completely while remaining on the support. The simulations confirm that complete dissolution is a viable pathway for small particles under the studied conditions."

2. It is also unclear how the authors validate the distinction between detached and fully dissolved particles in experiments where the location is identical. The same applies to redeposited and reattached particles. It seems more appropriate to classify particles as either dissolved or detached than to separate these processes into two distinct mechanisms.

Similarly to the previous answer, we agree that the distinction between these possible particle fates can be made clearer also when discussing the experimental results.

A particle which disappears from an IL-STEM image could, in principle, be either physically detached or fully dissolved, and images alone cannot confirm one or the other. In our decision tree, we classify "detachment" when a particle is present in the "before" image but absent in the "after" image with no visible residue, and "dissolution" when a particle shrinks but remains present. This operational distinction is practical for tracking, but we acknowledge that a particle

classified as "detached" might instead have dissolved completely (leaving no trace), and a particle classified as "dissolved" is unambiguous only for size reduction.

To reflect this inherent limitation, we have added the following sentence to the Results section where the decision tree is presented:

“Another inherent limitation is that for detachment events, a particle that disappears from an image could, in principle, be either detached or fully dissolved. For practical reasons, disappearing particles are conservatively classified as detached.”

3. The authors report that EDX measurements of different regions yield varying results in terms of the shift in the Pt/Co ratio, and attribute these effects to the sample's inherent heterogeneity. EDX maps of the same regions would also be helpful in order to better assess whether there is any correlation between the location of the particles on the carbon support and the observed changes in composition, and to evaluate whether pure Co contributes to the averaged results.

We agree with this reviewer’s suggestion that identical-location EDX maps would also be helpful to assess the observed changes.

We have performed IL-EDX on several catalyst regions at higher magnification in the as-purchased state and after potential cycling activation, constructed Pt and Co elemental maps, and noted their respective atomic percentages. The Pt and Co elemental maps, along with the HAADF-STEM images of the same regions, can be found in Supplementary Fig. 3, and the atomic percentages are in Supplementary Table 2.

The results reveal that different catalyst regions exhibit notably different ratios between Pt and Co at both lower and higher magnification. One of the imaged areas included several sites with apparent pure Co in the initial state, that was not noticed after activation. Regarding the suggestion to assess whether there is any correlation between the location of the particles on the carbon support and the observed changes in composition, this remains challenging because of the low contrast of the support and difficult systematic annotation. The variations in relative intensity in TEM images of Pt/C catalyst layers including the ionomer were also shown in a recent study by Pedram et al (J. Power Sources, 2026, 240009), where even their detailed image analysis relied on qualitative and semi-quantitative insights when appropriate. In this paper, we therefore limit ourselves to qualitative evidence of local carbon support changes without direct quantitative links to changes in composition. Nonetheless, we can use this data to confirm that these observed Co sites contributed to the average atomic percentages before activation, in contrast to the other regions of interest, as inquired by the reviewer.

The main takeaways from these new experiments were summarized in the following comment in the Results section of the manuscript: “Identical-location EDX for four regions of interest at higher magnification, performed before and after potential cycling activation, confirmed notably different ratios between Pt and Co, as evidenced by the elemental maps in Supplementary Fig. 3 and atomic percentages in Supplementary Table 2. Co-rich sites, observed in the initial state, influence the determined atomic percentages, and do not persist after activation.”

Additionally, a comment on the composition of the initial state of the sample from the beginning of the Results section was rewritten and expanded to:

“For the present study, we have performed energy-dispersive X-ray spectrometry (EDX) on four catalyst regions, spanning hundreds of nanometers in size. HAADF-STEM images of the investigated areas are in Supplementary Fig. 2, and the determined atomic percentages are

in Supplementary Table 1. At these lower magnifications, the Pt:Co atomic ratios ranged from 44.7:55.3 to 54.4:45.6. Then, EDX mapping was performed for four catalyst areas at higher magnification, as summarized in Supplementary Fig. 3 and Supplementary Table 2. The Pt:Co atomic ratios were noticeably more diverse, ranging from 48.5:51.5 for an area that exhibited Co-rich sites to 80.4:19.6, underlining the sample's heterogeneity in chemical composition."

We added the following details to the method description:

"Identical location EDX maps were acquired using a resolution of 128x128 pixels with a 0.1 ms dwell time and limiting them to 250 sweeps each in areas of ~38 nm^2 and ~48 nm^2. The combination of spot size and condenser aperture gave a STEM probe current of 48.9 pA. These parameters were chosen as a compromise to avoid large modifications in the material due to long beam exposure but at the same time retrieve enough X-rays counts for reliable maps."

4. Additionally, reference spectra of pure Pt and Co should be included in the XRD plot, and the diffraction peaks corresponding to pure Co should be labelled.

We have included reference diffraction patterns for pure Pt and Co Fm-3m phases in the XRD plot in Figure 1b and labeled diffraction maxima, associated with Co, with Miller indices.

The revised caption for Figure 1b now reads: "**b**, X-ray diffraction. Background-subtracted XRD pattern with Miller indices for the Fm-3m Pt-Co and Co crystal phases. Pt and Co reference diffraction patterns are displayed below the Pt-Co/C experimental data."

5. Finally, the authors attribute the lower current values observed in the cyclic voltammograms to a decrease in active sites. However, the cyclic voltammetry measurements in the supplementary information are presented as absolute current values. While a lower absolute current could be consistent with fewer active sites, this alone is insufficient to support such a conclusion. To validate this claim, the data should be normalised to current density.

We thank the reviewer for this important comment. We agree that attributing the lower currents to a decrease in the number of active sites is too specific based only on the absolute current response. Therefore, we have revised the manuscript to avoid overinterpretation.

To address this concern, we additionally analyzed the data, and here we include an example of two CVs where the currents were normalized to the surface area, obtained by the H_{upd} anodic charge after activation and after degradation. These normalized voltammograms show some changes in the electrochemical response, indicating that the Pt surface undergoes qualitative electrochemical modifications.

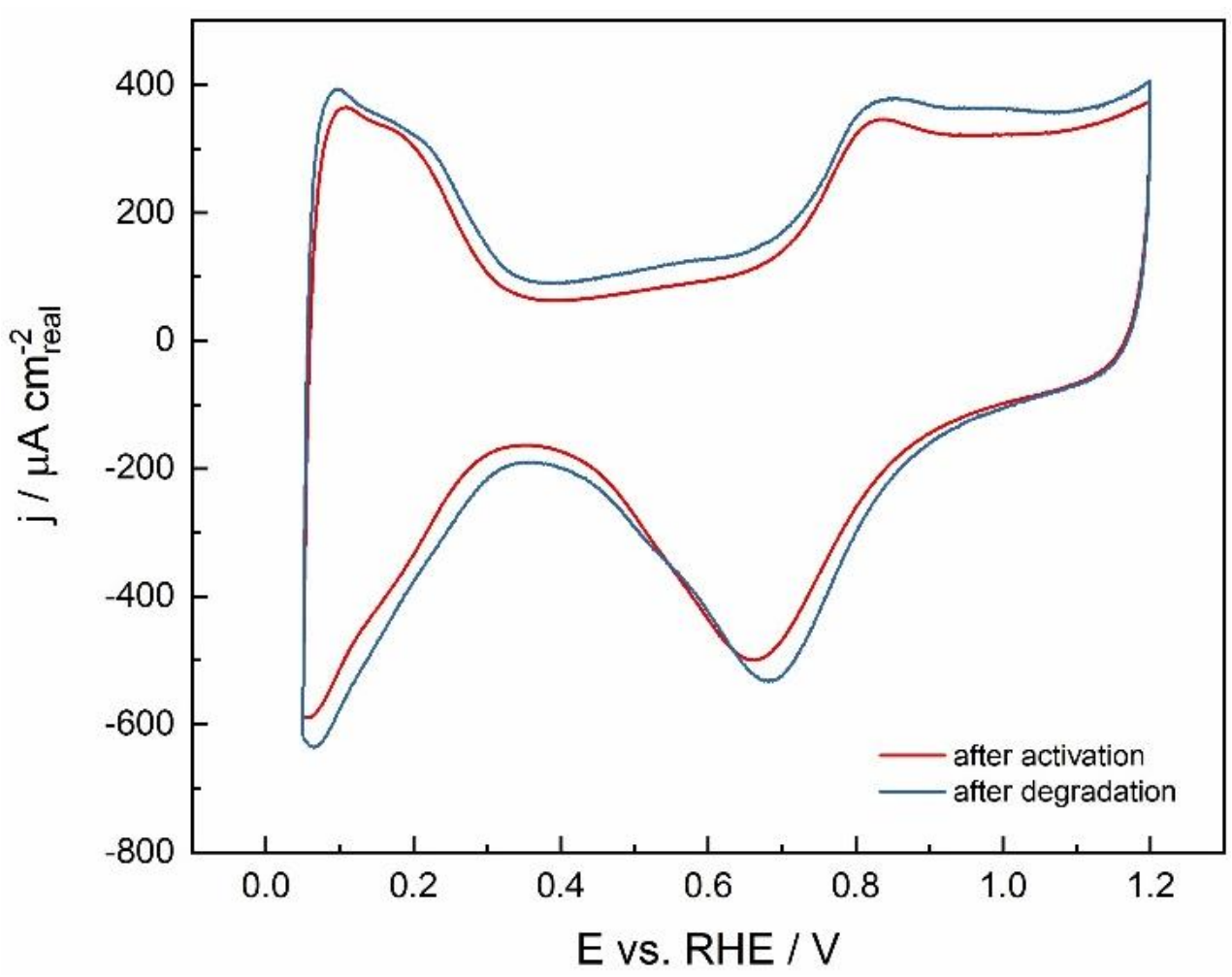

More specifically, because the CVs are normalized to the HUPD-derived Pt surface area, the Pt-related HUPD contribution is the same by construction. Any remaining difference in the relative capacitive current, therefore, reflects changes in the balance between Pt surface and carbon-support charging. In our case, the CV after degradation shows a larger carbon-support capacitive contribution relative to the normalized Pt surface area, as seen by higher current in most of the CV. This behavior is consistent with a lowering of Pt active sites, while the electrochemical surface of the carbon remains approximately unchanged. This interpretation is further supported by the absolute CVs (Supplementary Fig. 4c), where the double-layer current does not change (lines are one over another), indicating that the carbon surface contribution did not change. Therefore, the CVs we show in this rebuttal letter can be taken as qualitative evidence for a loss of Pt active sites after degradation.

In the revised manuscript, the claim connecting a lower absolute current to a lower number of active sites was rewritten and now reads:

"During degradation, only minor changes occurred: the final voltammogram exhibited slightly lower geometric-area-normalized current densities, which is qualitatively consistent with the loss of Pt surface area."

The reported lower absolute currents during a single cycling protocol remain consistent with IL-STEM results. The geometric-area-normalized current densities are now displayed in Supplementary Figures 4 and 5, showing this overall decrease in signal in terms of a current density instead of raw currents. To both figure captions, the following sentence was added: "Currents were normalized to the geometric area of the electrode (0.031425 cm^2)."

Reviewer #3 (Remarks to the Author):

Recommendation: Minor Revision

This manuscript presents a robust and scalable methodology that combines identical-location STEM (IL-STEM) with machine-learning-assisted image analysis to quantify electrocatalyst degradation at the single-particle level. In particular, the work effectively addresses longstanding limitations of conventional TEM studies, namely limited statistical sampling and challenges in quantitative analysis. By systematically tracking and analyzing hundreds of nanoparticles, the authors move beyond the small, often anecdotal datasets that have traditionally constrained this field, enabling statistically meaningful insights into degradation

mechanisms.
Importantly, as automated electron microscopy and high-throughput imaging continue to advance, approaches such as this are expected to play an increasingly important role in accelerating the discovery and optimization of durable catalyst materials. While the conceptual advance is, in my view, primarily methodological and somewhat incremental relative to prior IL-STEM studies, the study is well executed and establishes a versatile framework that should find broad application across a wide range of catalyst systems and degradation studies. I therefore recommend publication following minor revision.

We sincerely thank this reviewer for their kind evaluation of our manuscript and for recognizing the transferability of the presented approach.

More specific comments are:

1. Section c ("Theoretical modeling of Pt-Co particle evolution during activation") provides a useful framework for understanding the relationship between carbon support evolution and particle detachment. It would be helpful if the authors could briefly discuss whether carbon support corrosion can be quantified in relation to the various degradation pathways identified in this work, such as Ostwald ripening, particle detachment, dissolution, and particle coalescence. Such discussion would further highlight the interconnected nature of these degradation processes and broaden the mechanistic implications of the study.

We agree that linking carbon support corrosion (the location of such sites, and the magnitude of support changes) quantitatively to nanoparticle-specific degradation would provide a deeper mechanistic understanding and connect well with theoretical modeling.

However, quantifying carbon corrosion from IL-STEM images is challenging. Carbon has a weaker signal than Pt-Co nanoparticles, is often superimposed with particles, and shows subtle contrast changes that are difficult to distinguish from the background when the support is very thin. Especially in HAADF-STEM images, carbon is often barely visible. Annotating carbon features reliably for potential automated segmentation is therefore not straightforward.

In this study, we limit ourselves to qualitative identification of sites with apparent carbon support changes based on IL-BF-STEM images (Supplementary Fig. 14). In this revision, we reconsider the placement of arrows pointing to presumed carbon corrosion sites, and the revised Supplementary Fig. 14 now highlights sites we believe are more representative of changes to carbon with its characteristic fringes. A quantitative correlation between carbon corrosion and individual nanoparticle degradation events remains out of scope for this study, though it is a valuable direction for future work.

We have added the following discussion to the modeling part of the Results section, after introducing Supplementary Fig. 14:

“However, a direct quantitative correlation between support changes and specific nanoparticle degradation pathways is challenging from IL-STEM images due to the weaker signal and complex contrast of carbon. For the same reason, automated analysis of carbon support changes in these images remains difficult.”

2. The activation and accelerated degradation protocols employ substantially different potential windows, which may give rise to distinct degradation pathways and kinetics. It would be helpful for the authors to further discuss how the applied voltage window may influence the prevalence of the observed degradation mechanisms. Such discussion would help clarify the extent to

which the reported trends may depend on the electrochemical protocol and would further broaden the applicability of the conclusions.

We appreciate this suggestion to include a discussion on how the applied potential/voltage window may influence degradation and have added the following paragraph to the first part of the Results section, after explaining the dissolution trends of Pt and Co during potential cycling:

"The influence of the potential window extends beyond general dissolution trends. Existing literature confirms that both primary degradation mechanisms, metal dissolution and carbon corrosion, depend strongly on the applied potential window. Higher upper potential limits, encountered for instance during start-up/shut-down conditions in fuel cells, significantly increase both carbon corrosion and platinum dissolution, the latter due to more pronounced nanoparticle surface oxidation. Conversely, a narrower potential window reduces degradation rates. The primary mechanisms also trigger secondary ones, leading to loss of electrochemically active surface area and catalytic activity over time. Nevertheless, even a narrow window will produce noticeable degradation under prolonged operation. Thus, the choice of electrochemical protocol importantly shapes the reported degradation trends.[52]"

Other changes to the manuscript

A sentence explaining the image analysis workflow in the Abstract was changed to include "machine-learning-assisted detection" rather than "machine-learning-assisted segmentation" to correctly reflect the nature of the algorithm.

In the Methods section, Milli-Q water was once incorrectly stated as having 18.2 Ω cm, now changed to 18.2 MΩ cm.

In the caption of the Supplementary Figure 6 showing three-step IL-HAADF-STEM of Pt-Co/C, the activation and degradation protocols incorrectly included the rotation, which was removed.

When introducing the neural-network-based particle detection in the Results section, the following sentence was added directing the reader to more technical details: "The technical details about this step are available in Supplementary Note 1."

We have made the following changes to the paper according to the journal editorial guidelines:

- Two Results subheadings were shortened to a maximum of 60 characters, and subheading numbering was removed.
- The heading "Main" was exchanged with "Introduction", same for "Discussion" instead of "Conclusion". References in the SI are now titled "Supplementary References."
- Redundant commas were removed from figure captions describing individual figure panels.
- Formatting of the Supplementary Tables 11 and 12 has been changed to match the other tables.
- In the Data availability and Code availability statements, the DOI link to the Zenodo repository with the raw data and code was changed to the one that represents all versions of the repository.
- Units, variables, and subscripts/superscripts are written with Roman, italic, or bold characters, as appropriate per the guidelines. Inverse unit dimensions are now represented with negative integers. Aside from the text, these adjustments were also made in Fig. 6c-e and Supplementary Figs. 4, 5.
- Reference formatting was corrected.
- Abbreviations are defined upon their first use.
- Supplementary Information now begins with the article title.
- Unnecessary bold characters in the text were changed to normal.
- Items from the Supplementary Information are cited in the manuscript in the correct order (the three-step IL-STEM figure was moved to a more appropriate place in the Supplementary Information), and one vague reference to the document was exchange with a reference to actual items (Detailed mathematical formulations and parameters are provided in Supplementary Note 2 and Supplementary Table 11.).

Reviewer #1 (Remarks to the Author):

The addition of the new text "we consider a randomly selected dataset of several hundred nanoparticles to be statistically meaningful" is an opinion and does not appropriately address the comment to present a quantitative metric upon which the claim "statistically meaningful" can be substantiated. The authors should present a statistical analysis showing the increase in the data set converges to a desired metric (such as a predefined standard deviation or relative margin of error). Possible examples from the literature include C. Wouters et al. Sci Rep. 2025 Apr 7;15:11803, H. Yu et al. in ACS Nano 2022, 16, 8, 12083–12094, or K.M. Aviles ACS Nanosci. Au 2025, 5, 3, 117–127.

The reviewer cites relevant literature on particle size distributions, obtained with (S)TEM. Indeed, a statistical analysis showing the effect of dataset size on evaluating a key metric, in our case particle diameter or circularity, provides a more quantitative reason for claiming statistical meaningfulness than citing general guidelines.

To address this, we report a statistical analysis investigating the effect of subset size on the mean equivalent sphere diameter and circularity for approximately 700 Pt-Co nanoparticles from the degradation dataset in their initial state. The workflow follows examples from the suggested literature, namely from Wouters et al and Yu et al.

We drew random subsets of increasing size from 25 to 600 particles without replacement with 100 iterations per subset size, and calculated the mean particle diameter and circularity. The spread (standard deviation) of the 100 subset means at a given subset size is the standard error of the mean and an estimate of how much our reported mean would fluctuate had we annotated only that many particles. We express this spread in absolute and relative terms for particle diameter and circularity.

The analysis shows the relative error for the mean circularity remains below 1% across the entire range of tested subset sizes, while the same value for diameter falls below 2% for subset sizes greater than 150 particles and below 1% for subset sizes greater than 400 particles. The analysis is further described in Supplementary Note 1, and the results are summarized in Supplementary Fig. 6. (Consequently, the remaining Supplementary Notes and Figures were renumbered in the SI and the manuscript.)

These values confirm that a randomly selected dataset of several hundred nanoparticles from our specific Pt-Co/C electrocatalyst is sufficient to capture population-level trends with confidence, supporting the "statistically meaningful" claim. This is in line with the guidelines for dataset size from various sources that generally agree that analyzing several hundreds of nanoparticles is sufficient for a reliable result, including the examples cited by the reviewer:

- In Aviles et al (ACS Nanosci. Au, 2025, 5, 3, 117-127), the authors write: "A standard rule of thumb in the field is that attaining statistically meaningful estimates of particle properties often requires measurement of at least 200 particles", citing a protocol by the National Institute of Standards and Technology and Nanotechnology Characterization Laboratory.
- In Wouters et al (Sci Rep., 2025, 7, 15, 11803), they cite an OECD source that suggests measuring at least 300 particles in the case of a narrow size distribution, and two ISO standards that suggest measuring about 500 particles. Their own results confirm that the minimum particle count depends on how wide the distribution is, and that a significantly larger dataset size yields only negligible improvement.

In the manuscript, the statement “We consider a randomly selected dataset of several hundred nanoparticles to be statistically meaningful for this catalyst system, as this sample size is sufficient to capture population-level degradation trends within a particle ensemble with a relatively narrow size distribution.” was changed to:

“To substantiate that our sample size is adequate for this catalyst system, we performed a convergence analysis in which the mean particle diameter and circularity were calculated for randomly drawn subsets of increasing size (Supplementary Note 1, Supplementary Fig. 6). The relative standard error of the mean for circularity remains below 1% across all subset sizes tested, while that of particle diameter falls below 2% beyond 150 particles and below 1% beyond 400 particles, confirming that a randomly selected dataset of several hundred nanoparticles is sufficient to capture population-level degradation trends within this particle ensemble.”

The code used to generate the plots will be added to the existing Zenodo repository accompanying this manuscript.

Reviewer #2 (Remarks to the Author):

The authors' responses and amendments are appreciated. Thank you.

We appreciate the decision to review our manuscript and thank you for all feedback.

Reviewer #3 (Remarks to the Author):

All raised concerns are addressed. I support a publication of this manuscript.

Thank you for the feedback and for recognizing value in our manuscript.
